# Supplementary material for: Glucagon-like peptide 1 aggregates into low-molecular-weight oligomers off-pathway to fibrillation
Source: Biophys J. 2023 May 2;122(12):2475–88. doi: 10.1016/j.bpj.2023.04.027 (PMC10323027; doi:10.1016/j.bpj.2023.04.027)
Supplement: Document S1. Figures S1–S19 and Tables S1–S7 [file mmc1.pdf]

**Biophysical Journal, Volume *122***

## Supplemental information

# Glucagon-like peptide 1 aggregates into low-molecular-weight oligomers off-pathway to fibrillation

**Eva Přáda Brichtová, Monika Krupová, Petr Bouř, Viv Lindo, Ana Gomes dos Santos, and Sophie E. Jackson**

Supplemental information

# Glucagon-like peptide 1 aggregates into low molecular weight oligomers off-pathway to fibrillation

Eva Přáda Brichtová,[1] Monika Krupová,[2,3] Petr Bouř,[2] Viv Lindo,[4] Ana Gomes dos Santos[4] & Sophie E. Jackson[1,*]

[1]Yusuf Hamied Department of Chemistry, University of Cambridge, Cambridge, CB2 1EW, United Kingdom

[2]Institute of Organic Chemistry and Biochemistry, Academy of Sciences, Flemingovo náměstí 2, Prague 6, 166 10, Czech Republic

[3]Hylleraas Centre for Quantum Molecular Sciences, Department of Chemistry, UiT The Arctic University of Norway, Tromsø, N-9037, Norway

[4]AstraZeneca, Aaron Klug Building, Granta Park, Cambridge, CB21 6GH, United Kingdom

*Correspondence: Sophie E. Jackson. sej13@cam.ac.uk

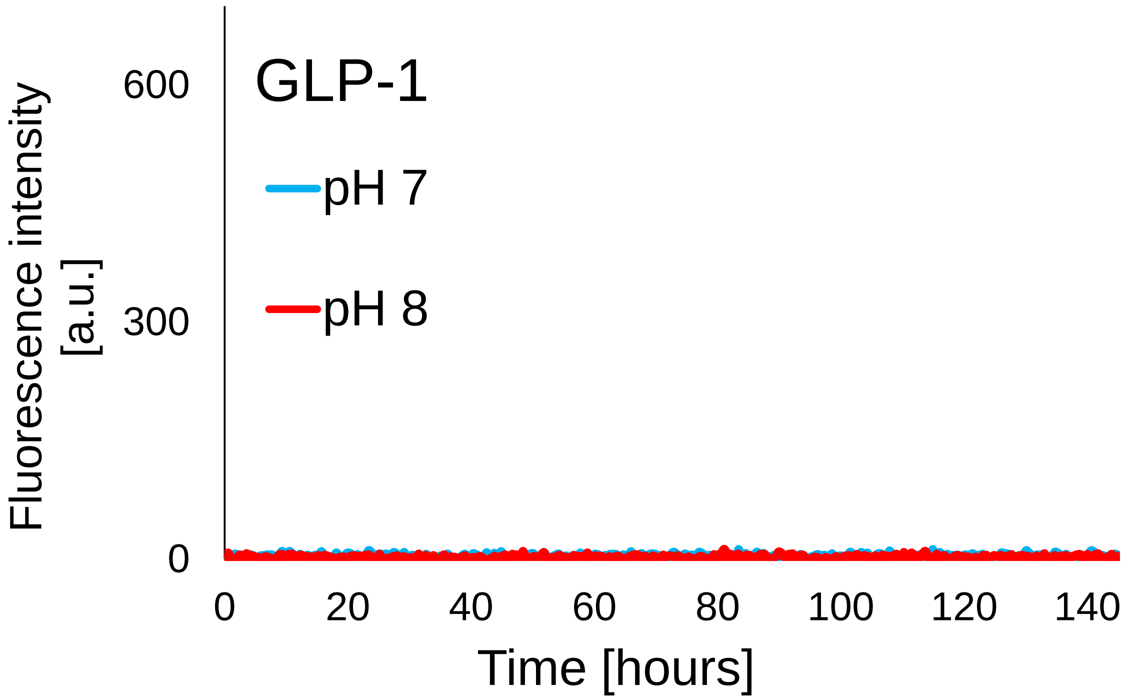


**Fig. S1: Fibrillation of GLP-1 at pH 7 and 8 monitored by a ThT assay.** GLP-1 at 43 µM concentration was incubated at pH 7 (10 mM sodium phosphate, shown in blue) and pH 8 (10 mM sodium phosphate, shown in red) at 37 °C with agitation over 6 days.

## Size exclusion chromatography analysis of low-molecular weight oligomers of GLP-1 and GLP-1-Am

**GLP-1 at pH 7, Figure 2A**

| Time [days] | Content [%] | | |
|---|---|---|---|
| | monomer | oligomers | other |
| 0 | 99 | 1 | 0 |
| 3 | 85 | 1 | 14 |
| 5 | 82 | 3 | 15 |
| 7 | 59 | 7 | 34 |
| 10 | 2 | 41 | 57 |

**GLP-1 at pH 8, Figure 2B**

| Time [days] | Content [%] | | |
|---|---|---|---|
| | monomer | oligomers | other |
| 0 | 98 | 2 | 0 |
| 5 | 81 | 15 | 4 |

**GLP-1-Am at pH 8, Figure 2C**

| Time [days] | Content [%] | | |
|---|---|---|---|
| | monomer | oligomers | other |
| 0 | 100 | 0 | 0 |
| 2 | 61 | 2 | 37 |
| 3 | 59 | 5 | 36 |
| 6 | 56 | 14 | 30 |
| 12 | 39 | 25 | 36 |
| 16 | 0 | 38 | 62 |

**GLP-1-Am at pH 7, Figure 2D**

| Time [days] | Content [%] | | |
|---|---|---|---|
| | monomer | oligomers | other |
| 0 | 100 | 0 | 0 |
| 3 | 20 | 2 | 78 |

**Table S1: A quantitative estimate of relative amounts of GLP-1 and GLP-1-Am monomer and oligomers present at different time points determined using SEC.** Values in this Table are obtained from the analysis of chromatograms shown in Fig. 2. A peak integration was performed in the Unicorn software of the ÄKTA instrument.

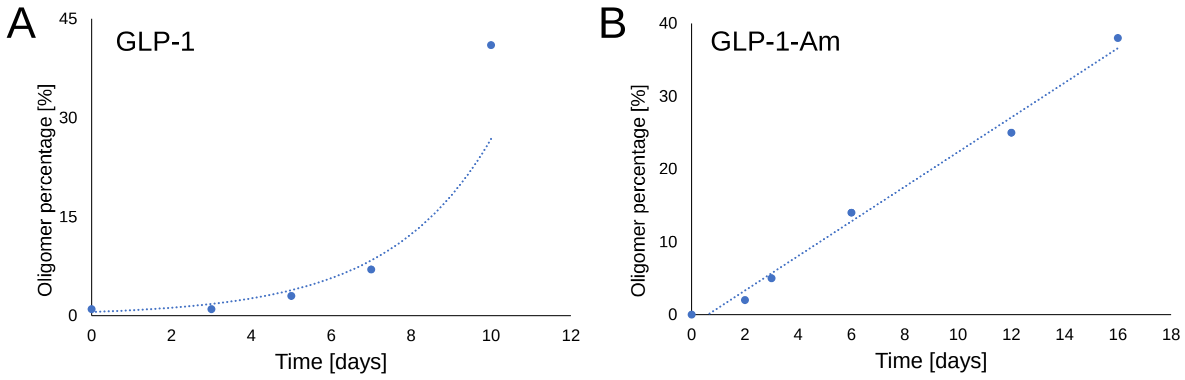


**Fig. S2: An increase of relative amounts of GLP-1 and GLP-1-Am oligomers as monitored by SEC.** The plots are based on the relative percentage of GLP-1 and GLP-1-Am oligomers at different time points given in **Table S1**. GLP-1 at 85 µM peptide concentration was incubated at 37 °C with agitation in 10 mM phosphate at pH 7 over 10 days (**A**). GLP-1-Am at 85 µM concentration was incubated at 37 °C with agitation in 25 mM phosphate at pH 7 over 16 days (**B**). The blue dotted line is the exponential (**A**) and linear regression (**B**) fit for the data points.

GLP-1 and GLP-1-Am low-molecular weight oligomer formation in different buffers monitored using SEC: ionic strength dependence

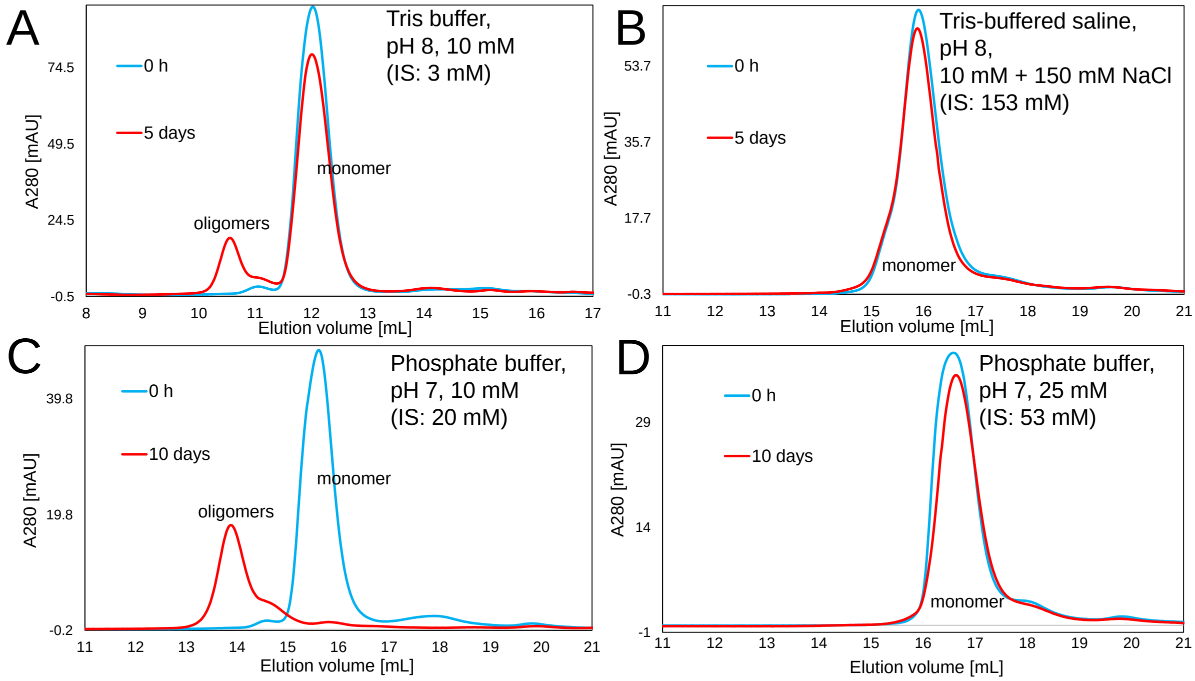


**Fig. S3: Formation of low-molecular weight oligomers of GLP-1 in low and high ionic strength (IS) buffers monitored using SEC.** (**A**) 150 µM GLP-1 in 10 mM Tris buffer at pH 8 (3 mM ionic strength) at time zero and after 5-day incubation (at 37 °C with shaking) were analysed using a Superdex 75 10/300 size exclusion column. (**B**) 150 µM GLP-1 in Tris-buffered saline (10 mM Tris +150 mM NaCl) at pH 8 (153 mM ionic strength) at time zero and after 5-day incubation (at 37 °C with shaking) were analysed using a Superdex 75 10/300 size exclusion column. (**C**) 85 µM GLP-1 in 10 mM sodium phosphate buffer at pH 7 (20 mM ionic strength) at time zero and after 10-day incubation (at 37 °C with shaking) were analysed using a Superose 12 10/300 size exclusion column. (**D**) 85 µM GLP-1 in 25 mM sodium phosphate buffer at pH 7 (53 mM ionic strength) at time zero and after 10-day incubation (at 37 °C with shaking) were analysed using a Superose 12 10/300 size exclusion column.

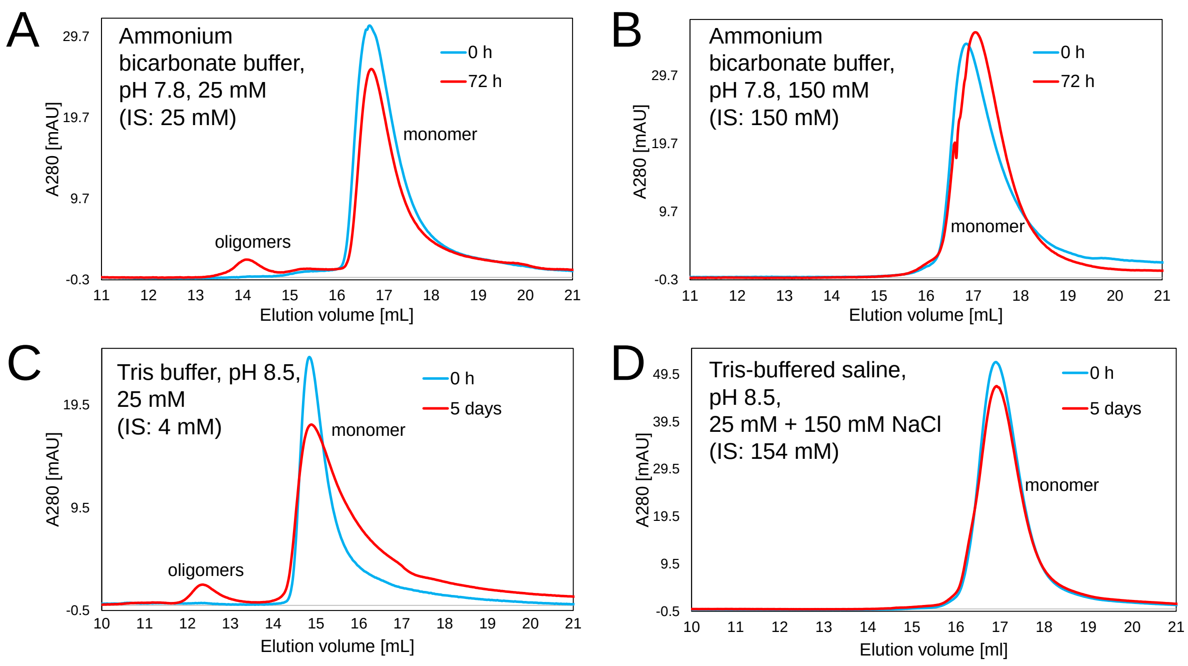


**Fig. S4: Formation of low-molecular weight oligomers of GLP-1-Am in low and high ionic strength (IS) buffers.** (**A**) 100 µM GLP-1-Am in 25 mM ammonium bicarbonate at pH 7.8 (ionic strength 25 mM) was analysed at time zero and after 72-hour incubation at 37 °C with agitation. (**B**) 100 µM GLP-1-Am in 150 mM ammonium bicarbonate at pH 7.8 (ionic strength 150 mM) was analysed at time zero and after 72-hour incubation at 37 °C with agitation. (**C**) 120 µM GLP-1-Am in 25 mM Tris buffer at pH 8.5 (ionic strength 4 mM) was analysed at time zero and after 5-day incubation at 37 °C with agitation. (**D**) 120 µM GLP-1-Am in Tris-buffered saline at pH 8.5 (ionic strength 154 mM, 25 mM Tris and 150 mM NaCl) was analysed at time zero and after 5-day incubation at 37 °C with agitation. All samples were analysed using a Superdex 75 10/300 size exclusion column.

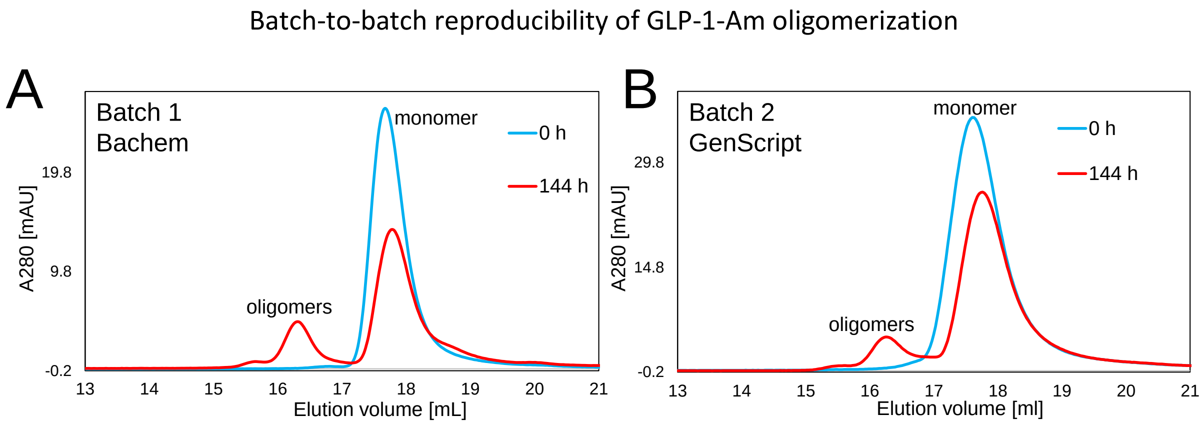


**Fig. S5: Batch-to-batch reproducibility of GLP-1-Am oligomerization.** 85 µM GLP-1-Am from Bachem (**A**) or GenScript (**B**) was incubated in 25 mM sodium phosphate buffer, pH 8 at 37 °C with agitation. Size-exclusion chromatograms of samples of both peptide batches were analysed on a Superose 12 10/300 column at time zero and after 144 h of incubation.

## Estimation of the size of low-molecular weight oligomers of GLP-1 and GLP-1-Am using size-exclusion chromatography

### Calibration of Superose 12 10/300 in 25 mM sodium phosphate buffer at pH 8.

| Protein standard | MW [Da] | Elution volume [mL] |
|---|---|---|
| Aldolase | 158,000 | 11.4 |
| Conalbumin | 75,000 | 12.2 |
| Ovalbumin | 44,000 | 12.6 |
| carbonic anhydrase | 29,000 | 13.9 |
| Trypsinogen | 24,000 | 14.5 |
| ribonuclease A | 13,700 | 15.1 |
| chymotrypsin inhibitor 2 | 9,265 | 15.9 |
| GLP-1-Am monomer | 3,355 | 17.7 |

**Table S2: Protein calibration standards and their elution volumes on a Superose 12 10/300 in 25 mM sodium phosphate buffer at pH 8.**

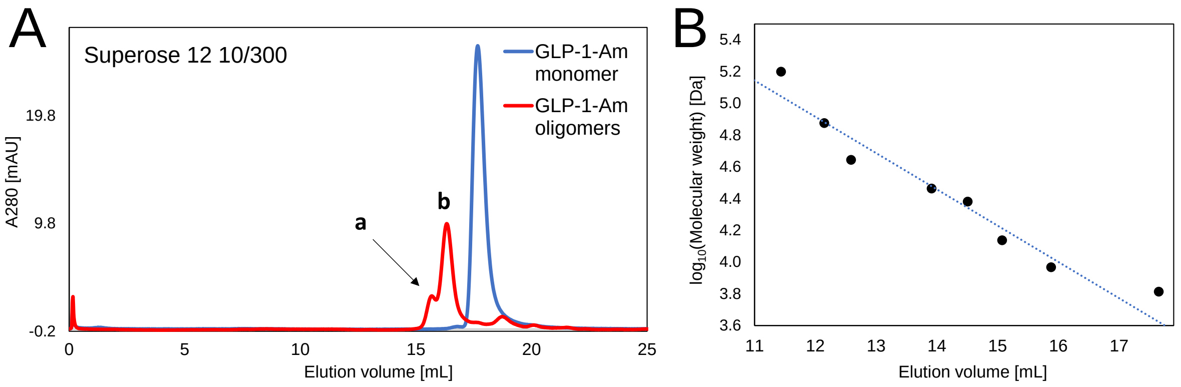


**Fig. S6: Elution profiles of the GLP-1-Am monomer and oligomers on a Superose 12 10/300 in 25 mM sodium phosphate buffer at pH 8 and column calibration curve.** The GLP-1-Am sample was analysed prior to incubation/shaking to induce aggregation (0 h, blue line) and after 16 days (red line) of incubation at 37 °C with continuous shaking at 180 rpm. Elution conditions: 25 mM sodium phosphate buffer at pH 8, flow rate 0.75 mL min$^{-1}$ (**A**). Calibration performed in 25 mM sodium phosphate buffer at pH 8 on a set of protein standards (Table S2) at room temperature. Elution was performed with a flow rate 0.75 mL min$^{-1}$ and 100 µL of approximately 100 µM of each protein was injected using a 200 µL injection loop. The protein standards elution volumes were plotted against the logarithm of their molecular weight and the correlation is described by a linear regression fit (blue dotted line) and its square of the correlation coefficient, where $V_e$ is the protein elution volume measured from the centre of a peak (**B**), see below.

$$log_{10}(\mathrm{MW/Da}) = -0.2285 \cdot V_e + 7.6579$$

$$R^2 = 0.9565$$

The elution volumes of the GLP-1-Am oligomers (**Fig. S6**) were 15.5 mL for oligomer **a** and 16.2 mL for oligomer **b**. Using the equation above, the size of oligomer **a** was calculated as 13 kDa and 9 kDa for oligomer **b** at pH 8.

## Calibration of Superdex 75 10/300 in 25 mM sodium phosphate buffer at pH 8.

| Protein standard | MW [Da] | Elution volume [mL] |
|---|---:|---:|
| ovalbumin | 44,000 | 9.8 |
| carbonic anhydrase | 29,000 | 11.4 |
| trypsinogen | 24,000 | 12.1 |
| ribonuclease A | 13,700 | 13.3 |
| chymotrypsin inhibitor 2 | 9,265 | 14.4 |
| GLP-1-Am monomer | 3,355 | 17.1 |

**Table S3: Protein standards and their elution volumes on a Superdex 75 10/300 in 25 mM sodium phosphate buffer at pH 8.**

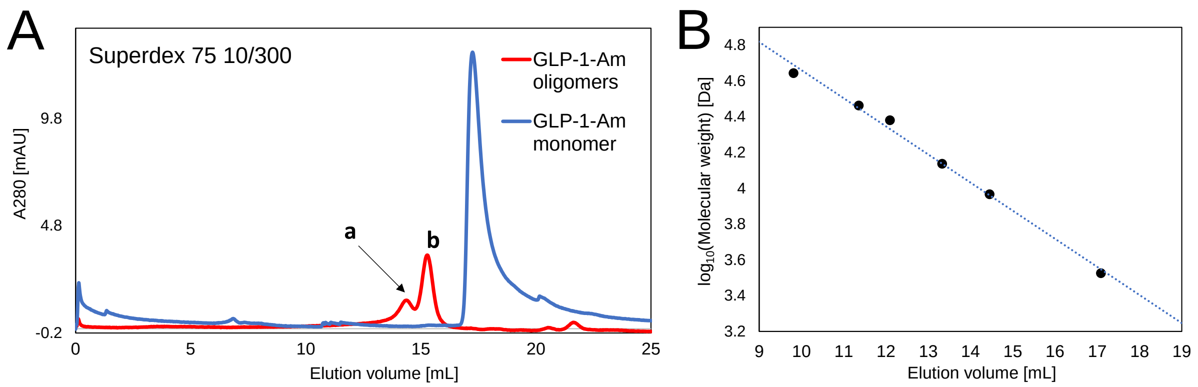


**Fig. S7: Elution profiles of GLP-1-Am monomer and oligomers on a Superdex 75 10/300 in 25 mM sodium phosphate buffer at pH 8 and column calibration curve.** GLP-1-Am samples were analysed prior to the incubation/shaking (0 h, blue line) and after 7 days (red line) of incubation at 37 °C with continuous shaking at 180 rpm. Elution conditions: 25 mM sodium phosphate buffer at pH 8, flow rate 0.75 mL min$^{-1}$ (**A**). Calibration performed in 25 mM sodium phosphate buffer at pH 8 on a set of protein standards (Table S3) at room temperature. Elution was performed at a flow rate of 0.75 mL·min$^{-1}$ and 100 µL of approximately 100 µM of each protein was injected using a 200 µL injection loop. The blue dotted line is the linear regression fit (**B**), see below.

$$log_{10}(\mathrm{MW/Da}) = -0.1571 \cdot V_e + 6.232$$

$$R^2 = 0.9935$$

The elution volumes of the GLP-1-Am oligomers (**Fig. S7**) were 14.4 mL for oligomer **a** and 15.3 mL for oligomer **b**. Using the equation above, the size of oligomer **a** was calculated as 9.3 kDa and 6.7 kDa for oligomer **b**.

Results of both SEC columns indicate that eluted species are low molecular weight oligomers of a size range from 6.7 kDa to 13 kDa at pH 8. These values correspond to a range of species from peptide dimers (6710 Da) to peptide tetramers (13 420 Da).

## Calibration of Superose 12 10/300 in 10 mM sodium phosphate buffer at pH 7.

| Protein standard | MW [Da] | Elution volume [mL] |
|---|---|---|
| Aldolase | 158,000 | 11.4 |
| Conalbumin | 75,000 | 11.7 |
| Ovalbumin | 44,000 | 10.7 |
| carbonic anhydrase | 29,000 | 13.1 |
| chymotrypsin inhibitor 2 | 9,265 | 15.8 |
| GLP-1 monomer | 3,355 | 15.6 |

**Table S4: Protein standards and their elution volumes on a Superose 12 10/300 in 10 mM sodium phosphate buffer at pH 7.**

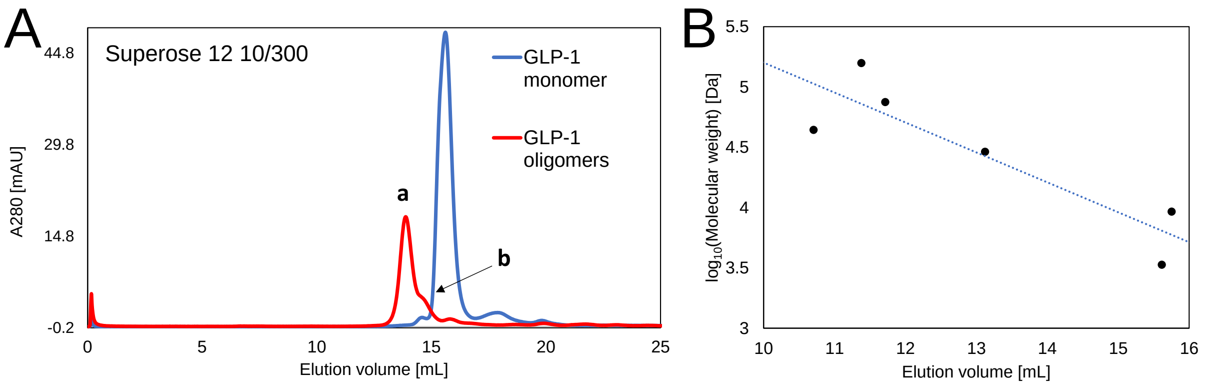


**Fig. S8: Elution profiles of GLP-1 monomer and oligomers on a Superose 12 10/300 in 10 mM sodium phosphate buffer at pH 7 and column calibration curve.** GLP-1 samples were analysed prior to the incubation/shaking (0 h, blue line) and after 10 days (red line) of incubation at 37 °C with continuous shaking at 180 rpm. Elution conditions: 10 mM sodium phosphate buffer at pH 7, flow rate 0.75 mL min$^{-1}$ (**A**). Calibration performed in 10 mM sodium phosphate buffer at pH 7 on a set of protein standards (Table S4) at room temperature. Elution was performed at a flow rate of 0.75 mL·min$^{-1}$ and 100 µL of approximately 100 µM of each protein was injected using a 200 µL injection loop. The blue dotted line is the linear regression fit (**B**), see below.

$$log_{10}(\mathrm{MW/Da}) = -0.2482 \cdot V_e + 7.684$$

$$R^2 = 0.7909$$

The elution volumes of the GLP-1-Am oligomers (**Fig. S8**) were 13.9 mL for oligomer **a** and 14.6 mL for oligomer **b**. Using the equation above, the size of oligomer **a** was calculated as 17.1 kDa and 11.5 kDa for oligomer **b** at pH 7.

## Sedimentation velocity analysis of low-molecular weight oligomers of GLP-1 and GLP-1-Am

| **GLP-1 fresh samples** | | | | **GLP-1-Am fresh samples** | | |
|---|---|---|---|---|---|---|
| $s_{20,w}$ [S] | MW [kDa] | Content [%] | | $s_{20,w}$ [S] | MW [kDa] | Content [%] |
| 0.15 | N/A* (monomer) | 42 | | 0.15 | N/A* (monomer) | 46 |
| 0.75 | 7.39 (dimer) | 45 | | 0.91 | 6.09 (dimer) | 46 |
| 1.67 | 23.9 (7-mer) | 10 | | 1.97 | 18.0 (5-mer) | 8 |
| | | | | | | |
| **GLP-1 7-day incubation** | | | | **GLP-1-Am 7-day incubation** | | |
| $s_{20,w}$ [S] | MW [kDa] | Content [%] | | $s_{20,w}$ [S] | MW [kDa] | Content [%] |
| 0.15 | N/A* (monomer) | 20 | | 0.15 | N/A* (monomer) | 48 |
| 0.60 | 4.73 (dimer) | 58 | | 0.77 | 7.49 (dimer) | 34 |
| 1.52 | 16.7 (5-mer) | 16 | | 1.24 | 14.5 (4-mer) | 13 |
| 2.73 | 43.6 (13-mer) | 6 | | 2.01 | 27.7 (8-mer) | 4 |

**Table S5: Quantitative estimation of mass and relative content of species detected in sedimentation velocity experiments.** The data correspond to the sedimentation profiles shown in Fig. 3. The molecular weight and relative content of detected species was calculated in a Sedfit program. * It was not possible to calculate the mass of GLP-1/GLP-1-Am monomer in a Sedfit program due to its limited sedimentation. The molecular weight of the GLP-1/GLP-1-Am monomer is approximately 3.35 kDa.

## Secondary structure content of monomeric GLP-1-Am, low-molecular weight oligomers and fibrils of GLP-1-Am determined from far-UV CD spectra

| GLP-1-Am | α-helix (%) | β-sheet (%) | turns (%) | disordered (%) |
|---|---|---|---|---|
| monomer | 31 | 15 | 23 | 31 |
| oligomers | 25 | 6 | 25 | 44 |
| fibrils | 5 | 39 | 15 | 41 |

**Table S6: Secondary structure content of monomeric GLP-1-Am, low molecular weight oligomers and fibrils of GLP-1-Am in 25 mM sodium phosphate buffer at pH 8 estimated from far UV CD spectra.** Far-UV CD spectra of GLP-1-Am monomer, low-molecular weight oligomers, and fibrils shown in Figure 4A were analysed using DichroWeb, Contin-LL method, dataset 3 (1–3).

## Fibrillation of GLP-1-Am at pH/pD 3 characterised by FT-IR, VCD and TEM

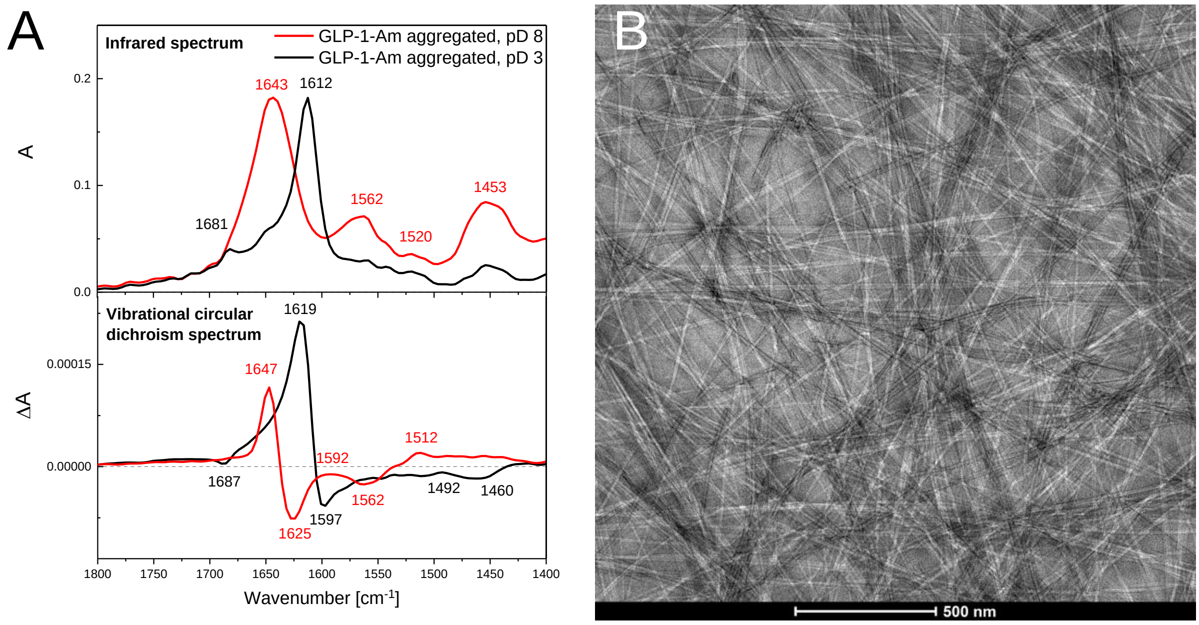


**Fig. S9: Aggregation of GLP-1-Am at pD/pH 3.** (**A**) FT-IR and VCD spectra of aggregated GLP-1-Am at pD 3 and pD 8. Samples of 1.2 mM GLP-1-Am in 25 mM deuterated sodium phosphate buffer at pD 3 and pD 8 were incubated for 8 days at 37 °C with 180 rpm agitation. The FT-IR and VCD spectra have been normalized to the amide I absorption. (**B**) GLP-1 samples at 85 µM concentration were incubated in 25 mM citrate at pH 3 at 37 °C with agitation for 6 days prior to the imaging. Aged samples were applied onto a carbon coated copper grid and negatively stained using 2 % uranyl acetate.

## Characterization of low-molecular weight oligomers of GLP-1 using far-UV CD and intrinsic Trp fluorescence

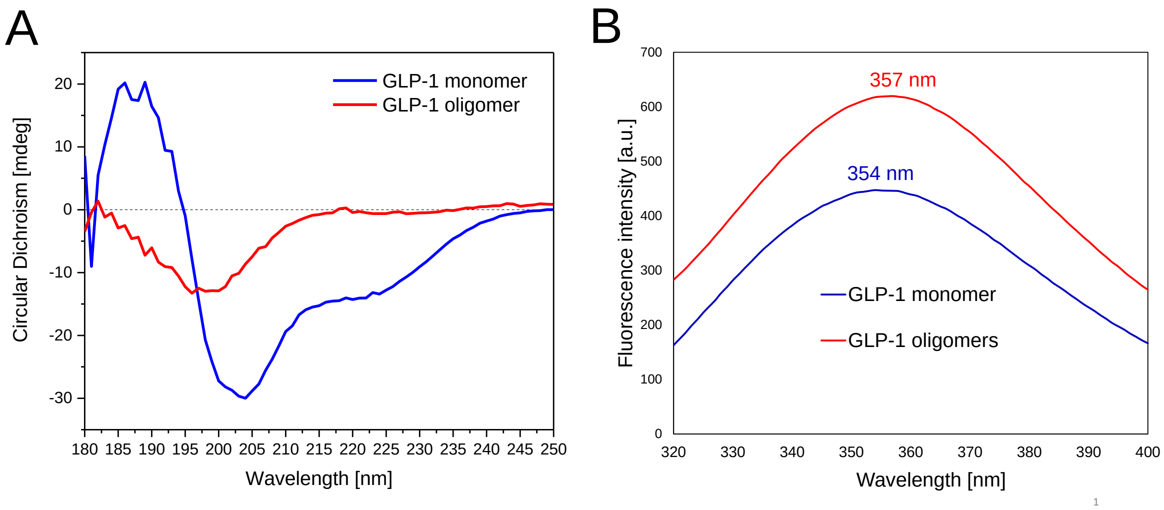


**Fig. S10: Far-UV CD spectra (A) and intrinsic tryptophan fluorescence emission spectra (B) of monomeric GLP-1 and low-molecular weight oligomers of GLP-1.** Spectra of the monomeric GLP-1 were measured immediately after peptide dissolution in 10 mM sodium phosphate buffer at pH 7. Spectra of GLP-1 oligomers were measured after 10-day incubation at 37 °C with agitation when all monomer was depleted as shown by SEC.

## SEC elution profiles and absorption spectra of GLP-1 monomer and low molecular-weight oligomers

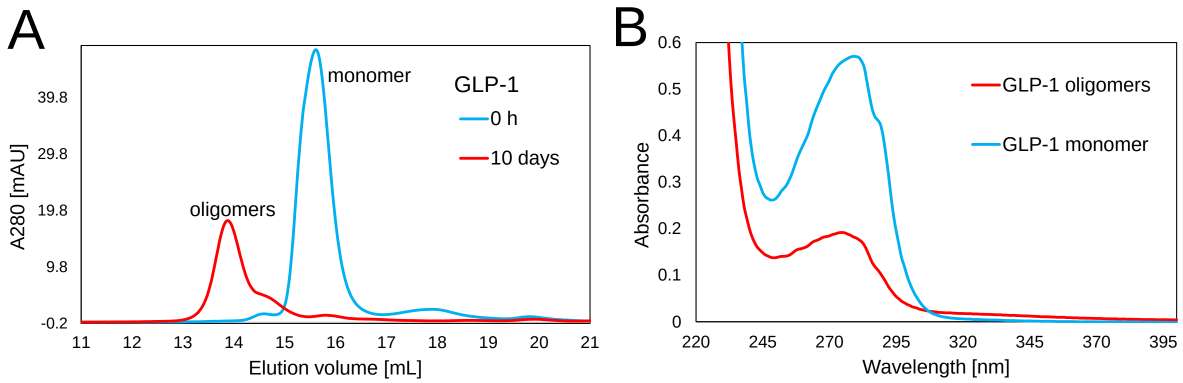


**Fig. S11: SEC elution volumes and absorption spectra of GLP-1 monomer and low molecular weight oligomers.** (**A**) GLP-1 was incubated in 10 mM sodium phosphate buffer at pH 7, at 37 °C with agitation. This sample was analysed using SEC (Superose 12 10/300) at different time points. Figure A shows the elution profile corresponding to the monomer of a freshly prepared GLP-1 sample along with the elution profile of oligomers of GLP-1 which were formed after 10 days of sample incubation. (**B**) UV absorption spectra of monomer and oligomer as described in (**A**). The oligomeric GLP-1 shows detectable light scattering as apparent from an increased absorption at around 320 nm. The concentration of oligomers is lower than the initial concentration of the peptide due to the competing aggregation/fibrillation processes that resulted in the formation of large insoluble aggregates which were removed by filtration prior to the analysis. Considering the small size of these oligomers, they show a surprisingly high level of light scattering as observed in the UV-Vis absorption spectra, which may suggest their further coagulation in the solution.

**Calcein-release assay with low-molecular weight oligomers of GLP-1-Am**

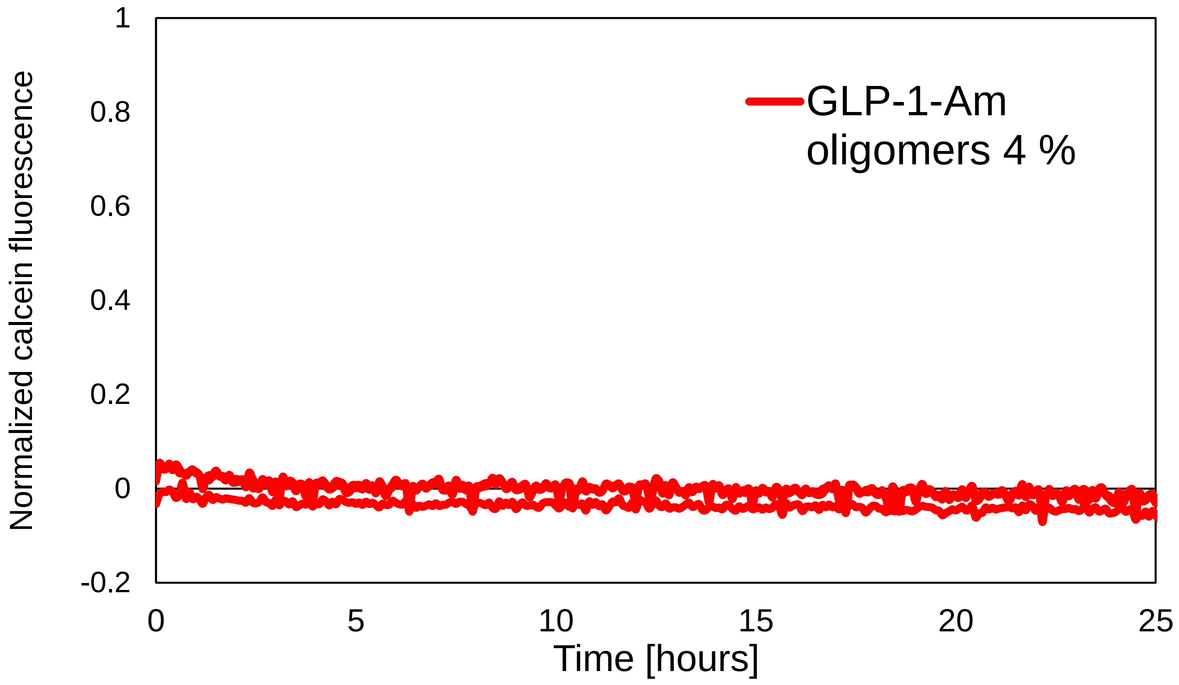


**Fig. S12: Calcein-release assay with 4 % of low-molecular weight oligomers of GLP-1-Am.** The assay was performed with 50 µM 1,2-dioleoyl-sn-glycero-3-phospho-L-serine (DOPS) vesicles at pH 8 in 37 °C over 25 hours.

**Calcein-release assay.** The assay was performed with 50 µM of calcein-encapsulated 1,2-dioleoyl-sn-glycero-3-phospho-L-serine (DOPS) vesicles of an average diameter of 125 nm (calcein concentration inside vesicles >70 mM). The assay buffer was 20 mM Hepes, 120 mM NaCl, 0.8 mM EDTA, pH 8.0. Low-molecular weight oligomers of GLP-1-Am were isolated using SEC from aged sample of 200 µM peptide incubated in 25 mM sodium phosphate at pH 8 for 7 days. Isolated oligomers were concentrated using Amicon Ultra Centrifugal Filter Unit with 3 kDa MWCO. The fluorescence emission signal at 520 nm was recorded every 5 minutes upon 484 nm excitation. Samples were incubated at 37 °C with agitation during the assay. The sample was measured in a triplicate. Fluorescence kinetic measurements were carried out using a microplate reader FLUOstar Omega (BMG Labtech). Samples calcein-encapsulated vesicle with 4 % (w/w) of GLP-1-Am oligomers were pipetted into a 96-well half-area plate (Corning 3694) and sealed with transparent tape to prevent samples from evaporation. The total volume of each sample in a well was 100 µL. Fluorescence was measured at a gain of 800 with 8 flashes per well. At the end of the assay 5 % (v/v) Triton X100 to each well which causes instant disruption of vesicles. The fluorescence signal was then normalized, taking the fluorescence value after the addition of 5 % (v/v) Triton X100 (complete disruption of vesicles) as 1.

## Assessment of GLP-1-Am oligomer stability using SEC

Re-injection stability

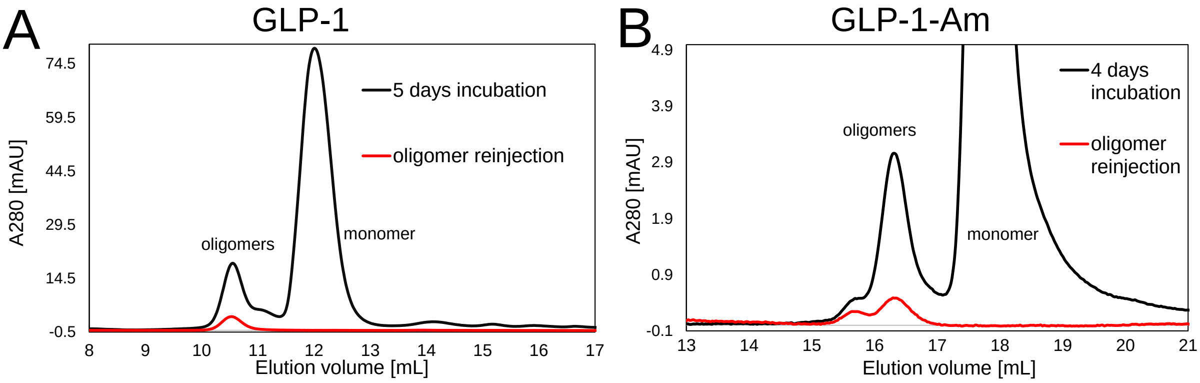


**Fig. S13: Stability of low-molecular weight oligomers of GLP-1 and GLP-1-Am upon reinjection onto a size-exclusion column.** (**A**) 150 µM GLP-1 was analysed after 5-day incubation at 37 °C with agitation in 10 mM Tris buffer at pH 8, oligomeric fractions were isolated and immediately reinjected onto the Superdex 75 10/300 size-exclusion column. (**B**) 85 µM GLP-1-Am was analysed after 4-day incubation at 37 °C with agitation in 25 mM sodium phosphate buffer at pH 8, oligomeric fractions were isolated and immediately reinjected onto the Superose 12 10/300 size-exclusion column.

Stability with respect to sonication, 20 % isopropanol, 40 % acetonitrile, or heating

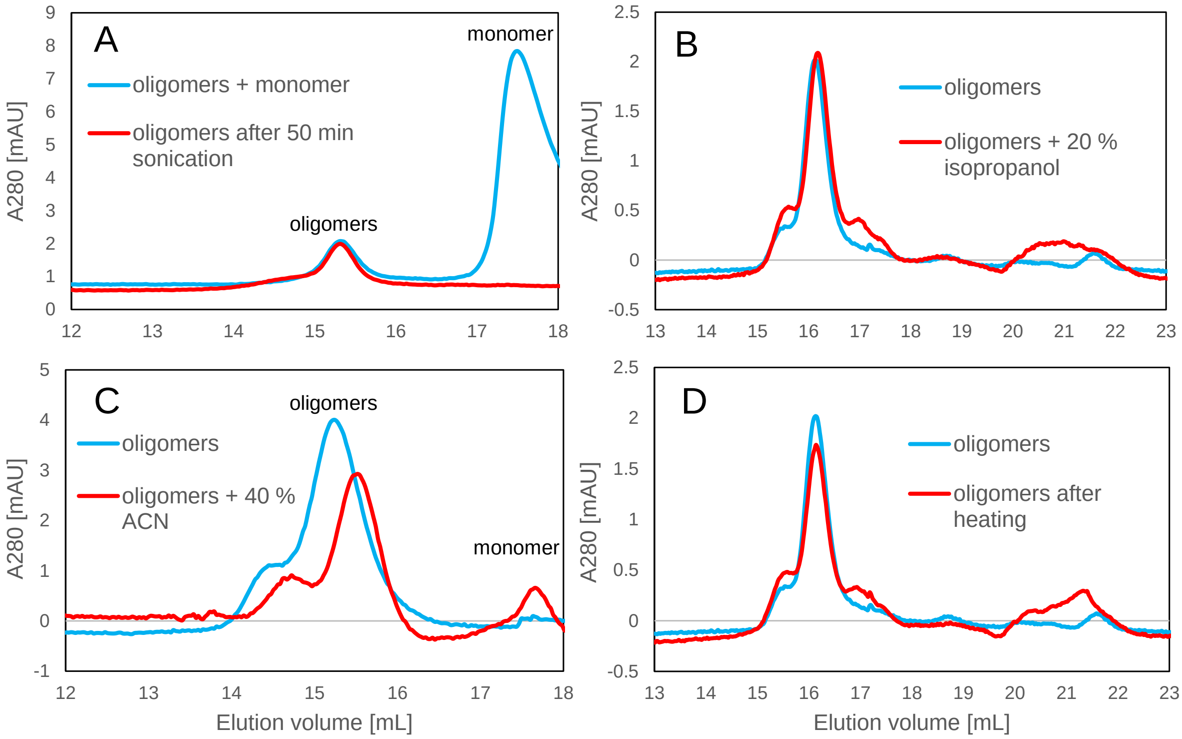


**Fig. S14: Assessment of GLP-1-Am oligomer stability using SEC.** SEC elution curves before (blue line) and after (red line) a stability test. Samples A and C were analysed on a Superdex 75 10/300 column, and B and D on a Superose 12 10/300 SEC column. After incubation (in 25 mM sodium phosphate buffer at pH 8 with continuous shaking at 180 rpm, for 3–9 days), oligomeric peaks were first separated by SEC (for B, C, D only) and, after the particular stability test, were reinjected onto a SEC column in 25 mM sodium phosphate buffer at pH 8. The samples were: A mixture of the monomer and oligomers sonicated for 50 min (**A**). Isolated oligomers incubated in 20% isopropanol for 1 hour (**B**), incubated in 40% acetonitrile (ACN) for 2 hours (**C**), or heated to 95 °C for 10 min (**D**).

**Liquid chromatography-mass spectrometry (LC-MS) analysis of GLP-1-Am oligomers**

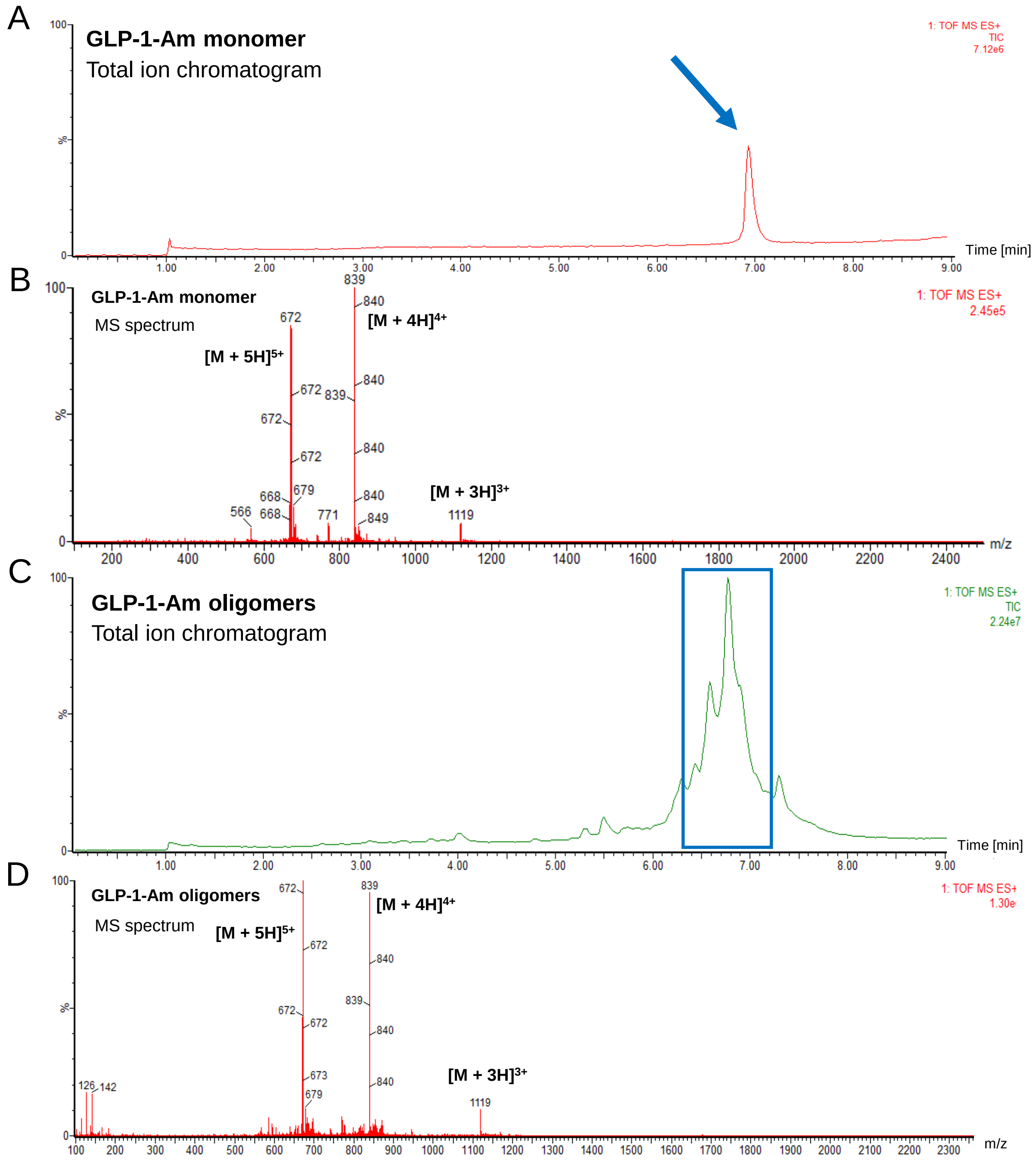


**Fig. S15: LC-MS analysis of monomeric and oligomeric GLP-1-Am.** Total ion chromatogram of monomeric GLP-1-Am – the blue arrow indicates the peptide peak on the total ion chromatogram (**A**), corresponding mass spectrum is shown in (**B**). LC-MS analysis of GLP-1-Am oligomers (**C, D**). The total ion chromatogram shows multiple overlapping peaks (**C**). All the peaks in the area marked with the blue rectangle correspond to the MS spectrum in (**D**) which is consistent with the spectrum of the GLP-1-Am monomer.

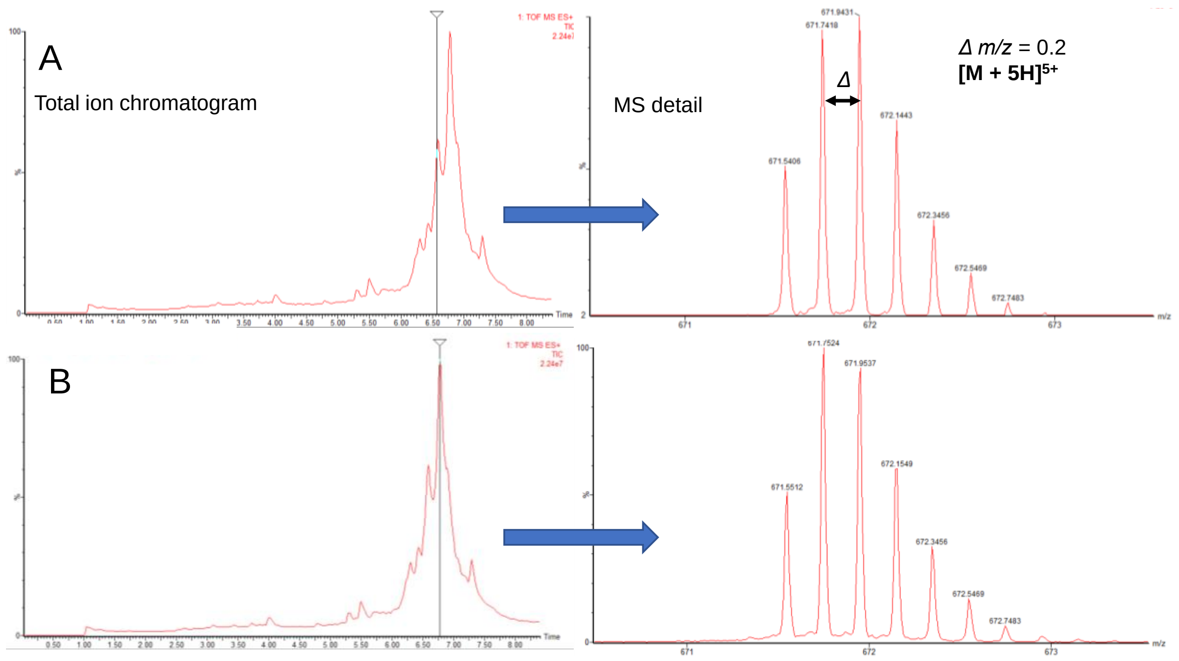


**Fig. S16: LC-MS analysis of GLP-1-Am oligomers – isotopic distribution.** The isotopic distribution of the peak with *m/z* around 672 was analysed at two different time points in the total ion chromatogram (**A** and **B**). Both isotopic distributions correspond exactly to the five times charged state of the GLP-1-Am monomer.

## LC-MS: Liquid chromatography gradient and elution conditions

| | | |
|---|---|---|
| **LC method** | | |
| **Flow rate** | 0.2 mL min$^{-1}$ | |
| **Run time** | 11.55 min | |
| **Sample injection** | 6 µL | |
| **Solvent system** | | |
| **A** | $H_2O$ + 0.1% formic acid | |
| **B** | 75% acetonitrile, 25% $H_2O$, 0.075% formic acid | |
| **Gradient** | | |
| **Time [min]** | **A (%)** | **B (%)** |
| 0.00 | 95 | 5 |
| 2.00 | 80 | 20 |
| 9.00 | 50 | 50 |
| 9.21 | 0 | 100 |
| 11.55 | 95 | 5 |

**Table S7: Waters' Xevo G2-S LC method.**

## Size exclusion chromatography and sedimentation velocity of freshly prepared samples of GLP-1-Am and βAsp_GLP-1-Am

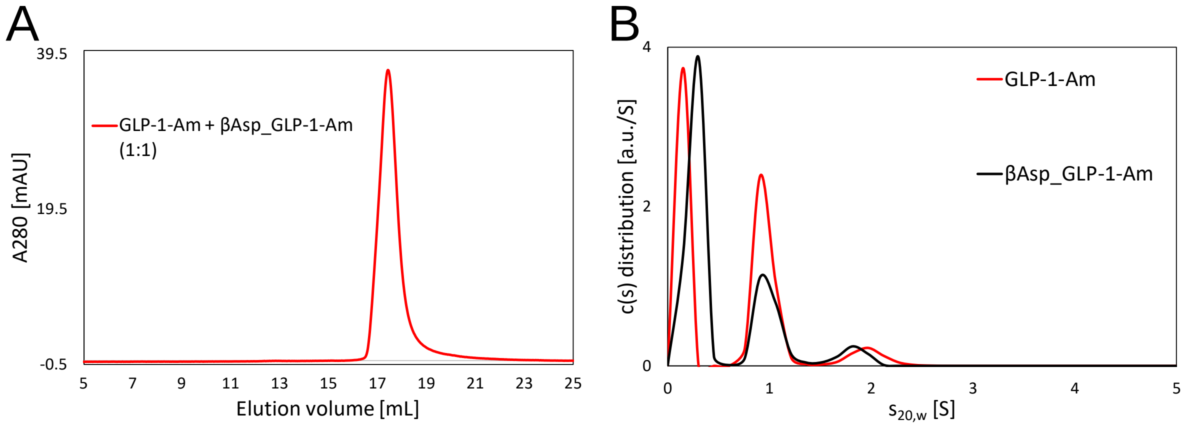


**Fig. S17: Size-exclusion chromatography and sedimentation velocity of freshly prepared GLP-1-Am and βAsp_GLP-1-Am. (A)** Size-exclusion chromatography of a freshly prepared mixed sample of GLP-1-Am and βAsp_GLP-1-Am in a 1:1 ratio at a total concentration of 85 µM in 25 mM phosphate, pH 8. Size-exclusion chromatography was performed using a Superose12 10/300 column. **(B)** Sedimentation velocity of freshly prepared samples of GLP-1-Am and βAsp_GLP-1-Am at 85 µM in 25 mM phosphate pH 8. After 2-hour temperature equilibration to 20 °C, the experiment was performed at 50 000 rpm using a Beckman Optima XL-I Analytical Ultracentrifuge. The interference sedimentation curves were collected as 300 scans (approx. 24 h run) and fitted to a continuous c(*s*) distribution model implemented in a Sedfit program (4). The sedimentation coefficient was corrected for the standard state of water at 20 °C ($s_{20,w}$).

**Isoelectric focusing gel electrophoresis of aged and fresh sample of GLP-1-Am, βAsp_GLP-1-Am and GLP-1**

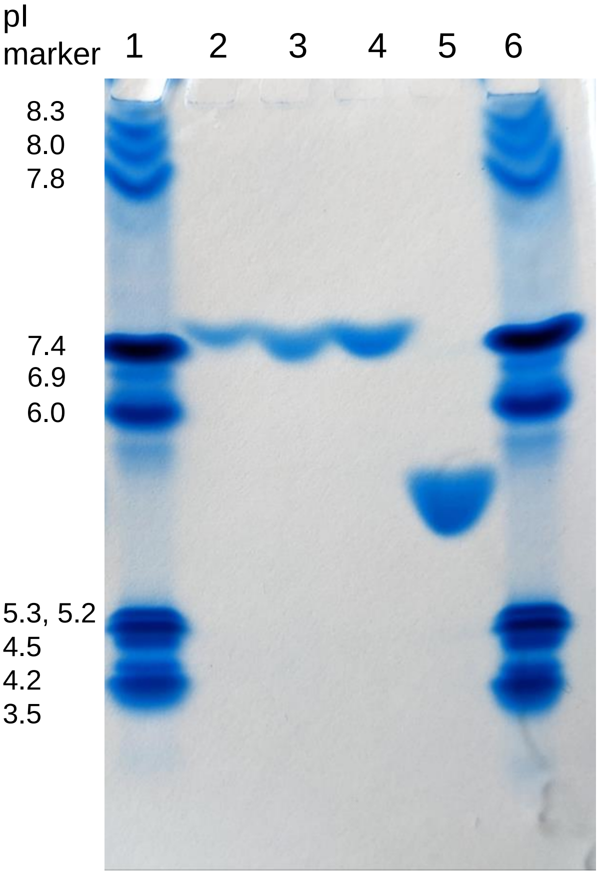


**Fig. S18: Isoelectric focusing gel electrophoresis of fresh and aged GLP-1-Am, βAsp_GLP-1-Am and GLP-1.** Lanes 1 and 6: pI protein standard markers, lane 2: GLP-1-Am incubated at 85 µM peptide concentration in 25 mM phosphate, pH 8, at 37 °C with agitation for 8 days, lane 3: GLP-1-Am (fresh sample), lane 4: βAsp_GLP-1-Am (fresh sample), lane 5: GLP-1 (fresh sample). Values of the isoelectric point (pI) standards are given on the left side of the gel image. pI value of samples in lanes 2, 3, 4 is at around 7.4; pI of a sample in lane 5 is somewhere in a range of 5.5–5.8 as determined from the gel.

**Isoelectric focusing electrophoresis.** The isoelectric point (pI) of aged and fresh samples of GLP-1-Am, βAsp_GLP-1-Am and GLP-1 was determined by isoelectric focusing (IEF) gel Novex™ pH 3-10 run in buffers from Novex™ pH 3–10 IEF Buffer Kit at a constant voltage of 200 V for 1 hour. Peptide samples were prepared in a sample buffer (Novex™ pH 3-10 IEF Buffer Kit) and immediately loaded onto the gel. A protein marker for isoelectric focusing SERVA Liquid mix 3–10 was run in parallel for pI reference. Gels were stained with InstantBlue (Expedeon).

## Effect of low-molecular weight oligomers on fibrillation kinetics:

## Thioflavin T assay of GLP-1-Am with the addition of pre-formed oligomers

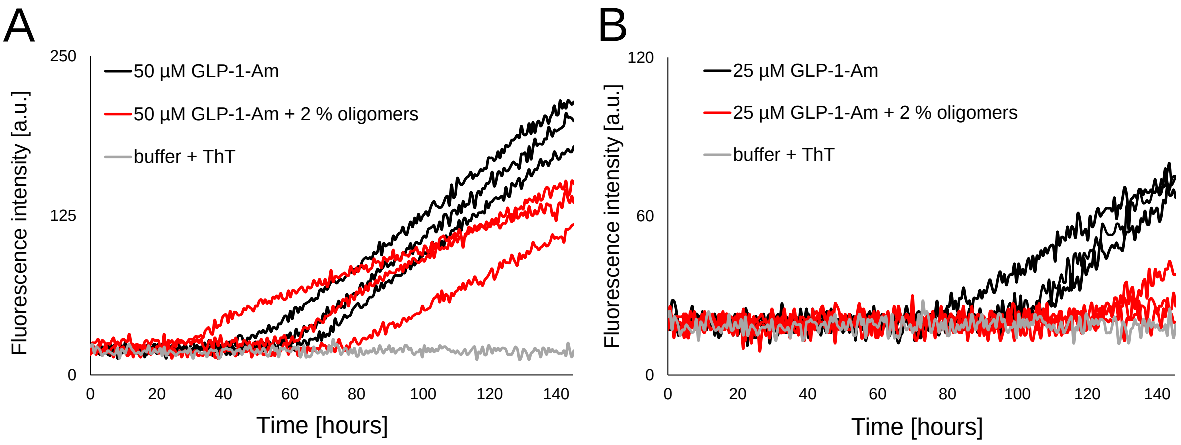


**Fig. S19: Thioflavin T assay of GLP-1-Am with the addition of pre-formed oligomers.** Samples of 50 (**A**) and 25 µM (**B**) GLP-1-Am in 25 mM phosphate at pH 8 with and without the addition of 2 % (w/w) of pre-formed purified GLP-1-Am oligomers were incubated with 50 µM thioflavin T at 37 °C with agitation for 6 days. All samples were prepared in a triplicate. GLP-1-Am oligomers were pre-incubated in 25 mM phosphate pH 8 for 5 days and then isolated using SEC.

## References:


1. Whitmore, L., and B.A. Wallace. 2008. Protein secondary structure analyses from circular dichroism spectroscopy: Methods and reference databases. *Biopolymers*. 89:392–400, doi: 10.1002/bip.20853.

2. Whitmore, L., and B.A. Wallace. 2004. DICHROWEB, an online server for protein secondary structure analyses from circular dichroism spectroscopic data. *Nucleic Acids Res.* 32:668–673, doi: 10.1093/nar/gkh371.

3. Sreerama, N., and R.W. Woody. 2000. Estimation of Protein Secondary Structure from Circular Dichroism Spectra: Comparison of CONTIN, SELCON, and CDSSTR Methods with an Expanded Reference Set. *Anal. Biochem.* 287:252–260, doi: 10.1006/abio.2000.4880.

4. Schuck, P. 2000. Size-distribution analysis of macromolecules by sedimentation velocity ultracentrifugation and Lamm equation modeling. *Biophys. J.* 78:1606–1619, doi: 10.1016/S0006-3495(00)76713-0.
