# Supplementary material for: Glucagon-like peptide 1 aggregates into low-molecular-weight oligomers off-pathway to fibrillation
Source: Biophys J. 2023 May 2;122(12):2475–88. doi: 10.1016/j.bpj.2023.04.027 (PMC10323027; doi:10.1016/j.bpj.2023.04.027)
Supplement: Document S2. Article plus supporting material [file mmc2.pdf]

Biophysical Journal
Article

Glucagon-like peptide 1 aggregates into low-molecular-weight oligomers off-pathway to fibrillation

Eva Přáda Brichtová,[1] Monika Krupová,[2,3] Petr Bouř,[2] Viv Lindo,[4] Ana Gomes dos Santos,[4] and Sophie E. Jackson[1,*]
[1]Yusuf Hamied Department of Chemistry, University of Cambridge, Cambridge, United Kingdom; [2]Institute of Organic Chemistry and Biochemistry, Academy of Sciences, Prague 6, Czech Republic; [3]Hylleraas Centre for Quantum Molecular Sciences, Department of Chemistry, UiT The Arctic University of Norway, Tromsø, Norway; and [4]AstraZeneca, Cambridge, United Kingdom

ABSTRACT The physical stability of peptide-based drugs is of great interest to the pharmaceutical industry. Glucagon-like peptide 1 (GLP-1) is a 31-amino acid peptide hormone, the analogs of which are frequently used in the treatment of type 2 diabetes. We investigated the physical stability of GLP-1 and its C-terminal amide derivative, GLP-1-Am, both of which aggregate into amyloid fibrils. While off-pathway oligomers have been proposed to explain the unusual aggregation kinetics observed previously for GLP-1 under specific conditions, these oligomers have not been studied in any detail. Such states are important as they may represent potential sources of cytotoxicity and immunogenicity. Here, we identified and isolated stable, low-molecular-weight oligomers of GLP-1 and GLP-1-Am, using size-exclusion chromatography. Under the conditions studied, isolated oligomers were shown to be resistant to fibrillation or dissociation. These oligomers contain between two and five polypeptide chains and they have a highly disordered structure as indicated by a variety of spectroscopic techniques. They are highly stable with respect to time, temperature, or agitation despite their noncovalent character, which was established using liquid chromatography-mass spectrometry and sodium dodecyl sulfate-polyacrylamide gel electrophoresis. These results provide evidence of stable, low-molecular-weight oligomers that are formed by an off-pathway mechanism which competes with amyloid fibril formation.

SIGNIFICANCE Protein or peptide aggregation into amyloid fibrils is a widespread phenomenon that plays a critical role not only in many neurodegenerative diseases but also in the stability of protein- and peptide-based biopharmaceuticals. The aggregation proceeds via multiple oligomeric states, which may be either directly on-pathway to amyloid formation or they may represent off-pathway aggregates incapable of direct conversion into fibrils. Here, we report the formation of stable, noncovalent low-molecular-weight oligomers during the aggregation of the therapeutic peptide glucagon-like peptide 1. The formation of these oligomers competes with the fibrillation process and the oligomers are shown to be structurally distinct from peptide monomers or fibrils. Our findings provide a greater insight into the processes that are off-pathway to protein and peptide amyloid formation.

INTRODUCTION

Glucagon-like peptide 1 (GLP-1) is a 31-residue peptide hormone responsible for regulation of blood glucose levels and other physiological functions (1,2). GLP-1 is of a great interest to the pharmaceutical industry as a therapeutic agent for the treatment of type 2 diabetes. Several GLP-1 analogs are already commercially available (3,4). However, previous studies have reported the self-association of GLP-1 into amyloid fibrils over a wide range of conditions (5–7), which hinders its pharmaceutical use.

The formation of amyloid fibrils is a multistep process: first, the monomeric peptide or protein self-associates into oligomeric structures; these can then form a critical nucleus which can further elongate, usually rapidly, to form amyloid fibrils. Classical fibrillation of peptides exhibits sigmoidal kinetics with three distinct phases—a lag phase (during which primary nucleation takes place), a growth phase (which consists primarily of elongation and secondary nucleation processes), and a final plateau phase (when monomer is depleted or equilibrium is reached) (8–11). Monomeric species dominate in the lag phase and fibrils

Submitted September 2, 2022, and accepted for publication April 27, 2023.
*Correspondence: sej13@cam.ac.uk
Editor: Samrat Mukhopadhyay.
https://doi.org/10.1016/j.bpj.2023.04.027
© 2023 Biophysical Society.
This is an open access article under the CC BY license (http://creativecommons.org/licenses/by/4.0/).

in the plateau phase, while during the growth phase their concentrations vary (11). In this classical description of a nucleation-propagation mechanism, both the half-life and the lag time decrease with increasing peptide or protein concentration (12,13).

Oligomeric species occurring in the fibrillation process are of a crucial importance not only from a mechanistic point of view but also because they are nowadays believed to be the main species responsible for the toxicity and pathology of protein misfolding-related diseases such as Alzheimer's, Huntington's, and Parkinson's diseases (12,14–17). The size, shape, and morphology of oligomers are very diverse, as are their half-lives and stabilities (15,16,18–20). Nowadays, the terminology that is widely used to describe oligomers is based on the kinetic role of the oligomers in the fibrillation process. Using this terminology, oligomers are referred to as on-pathway if they are capable of direct transformation into amyloid fibrils, or off-pathway if they are produced by a side reaction that does not lead directly to amyloid fibril formation (21,22). Nevertheless, this definition is rather narrow and may not always precisely reflect the very diverse nature of these species (19). Oligomeric species populated during the aggregation of peptides and proteins are usually of a transient nature, which complicates their detection and structural characterization (11,12,23). However, examples of more stable oligomeric species have also been reported (14,18,23–27). A long-lived oligomer resistant to fibrillation has been reported in amyloid-β peptide aggregation (28). Amyloid-β peptide has also been shown to irreversibly assemble into dimers and tetramers (29). Highly stable unstructured oligomers have been observed with α-synuclein, amyloid-β peptide, and islet amyloid polypeptide after inhibiting the fibrillation by the addition of small molecules (30,31). A stable helical dimer has been identified in NMR studies of GLP-1 in a mixture of H_2O and trifluoroethanol (32).

The intrinsic physical instability of GLP-1 remains a challenge in drug processing and formulation. This peptide hormone is also one of the few aggregation-prone peptides/proteins that exhibit unusual fibrillation kinetics where, under specific conditions, the lag time increases with increasing peptide concentration (7). This behavior has been also reported for human calcitonin (33), ribosomal protein S6 (34), immunoglobulin light chain (35), and liraglutide (36). The unusual dependence of lag time on the peptide concentration has been attributed to various off-pathway, self-assembly processes that affect the rates of fibrillation. Despite the increasing evidence for off-pathway oligomers, there is still a need to elucidate their structural properties, stabilities, and the mechanisms by which such off-pathway species form and affect the fibrillation kinetics.

In this study, a variety of spectroscopic methods were used to probe and monitor aggregation, as well as characterize the oligomeric species observed, including thioflavin T (ThT) assays. ThT is known for its capability of binding to the cross-β sheet structure of amyloid fibrils resulting in an increase in its fluorescence at around 480 nm, and has been widely used in fibrillation assays (37). Using size-exclusion chromatography (SEC), we report the observation of stable, low-molecular-weight oligomers of GLP-1 and its C-terminally amidated analog, GLP-1-Am. These oligomers are shown to be highly stable with respect to a number of different stresses and are likely to be off-pathway in the fibrillation process. As shown by multiple spectroscopic methods, these off-pathway oligomers have a highly disordered structure. Since the formation of low-molecular-weight oligomers was observed for both GLP-1 and GLP-1-Am, the C-terminally amidated analog was included in the study to solidify the results shown. Moreover, C-terminal amidated variants of GLP-1 and their derivatives are also produced and tested in the pharmaceutical industry and therefore are of direct relevance. However, the presented study is not a direct comparison of the behavior of GLP-1 versus GLP-1-Am, but a characterization of the low-molecular-weight oligomers that contribute to the unusual aggregation kinetics observed for both variants.

MATERIALS AND METHODS

GLP-1 and GLP-1-Am

GLP-1(7–37), H-HAEGTFTSDVSSYLEGQAAKEFIAWLVKGRG-OH, with a molecular weight of 3355 Da, was purchased from GenScript (Genscript, USA) in the form of an acetate salt with 98.1% purity. The residual content of the TFA salt was 0.94% as determined by GenScript. The peptide was produced by GenScript using solid-phase peptide synthesis and purified using HPLC.

GLP-1-Am(7–37), H-HAEGTFTSDVSSYLEGQAAKEFIAWLVKGRG-NH_2, has a molecular weight of 3355 Da. Two different batches were used in the experiments. Both batches were produced by solid-phase peptide synthesis and purified using HPLC. Batch 1 was purchased from Bachem (Switzerland) in the form of an acetate salt with 96.7% purity. The residual content of the trifluoroacetic acid TFA counterion was not specified. Batch 2 was purchased from GenScript in the form of an acetate salt with 99.2% purity. The residual content of the TFA salt was 0.02% as determined by GenScript.

βAsp_GLP-1-Am(7–37), H-HAEGTFTS{Beta-Asp}VSSYLEGQAAKEFIAWLVKGRG-NH_2, with a molecular weight of 3355 Da, was purchased from GenScript in the form of an acetate salt with 92.5% purity. The peptide was produced by GenScript using solid-phase peptide synthesis and purified using HPLC.

Peptides obtained from GenScript and Bachem were used without further purification.

GLP-1 and GLP-1-Am sample preparation and aggregation

Peptide powder was dissolved in the appropriate buffer. After peptide dissolution, the sample was filtered through a 0.22-μm filter (PES membranes, Millex, Germany). The concentration of the peptide was determined spectrophotometrically on a Cary 60 UV-vis spectrophotometer (Agilent Technologies, USA) using the Beer-Lambert law and a theoretical extinction coefficient of 6990 M^{-1} cm^{-1} at 280 nm. Samples were incubated in 1.5-mL plastic microcentrifuge tubes (STARLAB, Germany), sealed, and wrapped in aluminum foil to protect from sunlight. Incubation/aggregation was performed in an incubator shaker (Innova 43, Eppendorf Innova, Germany) at 37°C with 180 rpm agitation.

Kinetics of aggregation—ThT binding assays

Fluorescence kinetic measurements were carried out using a FLUOstar Omega microplate reader (BMG Labtech, Germany). Peptide samples at a given concentration were incubated at 37°C with 50 μM ThT. Peptide samples with ThT were pipetted into a 96-well half-area plate (Corning 3881, USA) and sealed with tape (Costar Thermowell) to prevent samples from evaporation. The total volume of each sample in a well was 100 μL. Bottom reading of the plate was performed every 30 min with 5 min of shaking before each reading (orbital shaker mode at 600 rpm). ThT binding to fibrils was monitored by recording the fluorescence emission at 482 nm using an excitation filter at 448 nm. Fluorescence was measured at a gain of 500 with 8 flashes per well.

Size-exclusion chromatography

Analytical size-exclusion chromatography (SEC) was performed on an ÄKTA FPLC system (GE Healthcare, USA), using a Superose 12 10/300 or a Superdex 75 10/300 column (both GE Healthcare). Samples were loaded using 100- or 200-μL loops. Before loading, the samples were filtered through a 0.22 μm filter. All samples were eluted at a flow rate of 0.75 mL min^{-1} at room temperature and UV absorbance detection at 280 nm through a 0.5-cm flow cell was used. Globular protein standards (Tables S2–S4) were used for column calibration under the same elution conditions as the experiments for the GLP-1-Am or GLP-1 oligomers—25 mM sodium phosphate buffer (pH 8) and 10 mM sodium phosphate buffer (pH 7), respectively. The elution volume of each protein standard was plotted against the logarithm of its molecular weight. A linear regression of this plot was used to determine the molecular weight of the observed GLP-1 and GLP-1-Am oligomers. A peak integration was performed in the Unicorn software of the ÄKTA instrument.

Sedimentation velocity

Sedimentation velocity experiments were performed using a Beckman Optima XL-I Analytical Ultracentrifuge (Beckman Coulter, USA) equipped with an An-60 Ti rotor. Samples of 85 μM peptide concentration were either freshly prepared before the measurement or incubated for 7 days at 37°C with 180 rpm agitation. After 2-h temperature equilibration of samples in the centrifuge to 20°C, the experiment was performed with centrifugation at 50,000 rpm. The interference sedimentation curves were collected as 300 scans (approximately 24 h run time) and fitted to a continuous c(s) distribution model implemented in the Sedfit program. The sedimentation coefficient was corrected for the standard state of water at 20°C ($s_{20,w}$). The molecular weight and relative content of detected species was calculated using the Sedfit program.

Transmission electron microscopy

GLP-1-Am (43 μM) in 25 mM sodium phosphate buffer was imaged after 6 days of incubation at 37°C with agitation. This sample was diluted fivefold directly before imaging, then 2 μL of the sample was deposited onto a freshly glow discharged copper grid 300 mesh with a carbon coating (Agar Scientific, United Kingdom), which was glow discharged using a Quorum Technologies (United Kingdom) GloQube system before the sample application. After 1 min the excess of the sample was removed from the copper grid using filter paper, then the sample was negatively stained with 2 μL of 2% (w/w) uranyl acetate solution for 15–30 s and the excess of the stain was again removed using filter paper. Samples were imaged using a Thermo Scientific (USA) Talos F200X G2 transmission electron microscope with an acceleration voltage of 200 kV.

Circular dichroism spectroscopy

Circular dichroism spectra were measured on a Chirascan CD spectrometer (Applied Photophysics, United Kingdom). Far-UV circular dichroism (CD) spectra over the range 180–250 nm were measured in a 1-mm pathlength cuvette and the measurement was performed with a 1-nm step size and with a 1-nm spectral bandwidth. Near-UV CD spectra over the range 250–350 nm were measured with 0.5-nm step size and 1-nm spectral bandwidth and in a 2-mm pathlength cuvette. The final spectrum was obtained as an average of three identical scans and the spectrum of the pure buffer was subtracted. All measurements were performed at room temperature. CD intensity is presented in millidegree units due to difficulties in the precise determination of the concentration of fibrils and oligomers, which was approximately 10 μM for oligomers and 50 μM for fibrils. The concentration of monomeric GLP-1 and GLP-1-Am was 85 μM.

Infrared absorption spectroscopy and vibrational circular dichroism

Infrared (IR) and vibrational circular dichroism (VCD) spectra were measured on a ChiralIR instrument (BioTools, USA) using BaF_2 windows, 50 μm optical pathlength, 8 cm^{-1} resolution, and 10 blocks of 2048 scans. Samples of aggregated GLP-1-Am were measured at a concentration of 4 mg mL^{-1} (1.2 mM) in deuterated 25 mM phosphate buffer at pD 3 or 8. All measurements were performed at room temperature and deuterated phosphate buffer spectra (of corresponding pD) were subtracted as a baseline. Both IR and VCD spectra were normalized to equal the amide I absorption.

Intrinsic tryptophan fluorescence

Intrinsic tryptophan fluorescence spectra were measured on a Cary Eclipse Fluorescence Spectrophotometer (Agilent Technologies, USA). Spectra were obtained using an excitation wavelength of 280 nm and emission spectra were recorded between 300 and 400 nm with a step of 1 nm. Emission and excitation band passes of 10 nm, and a voltage on the photomultiplier tube of 550 V, were used. Samples were measured in a 120-μL quartz cuvette (Hellma Analytics, Germany).

Denaturing protein gel electrophoresis — SDS-PAGE

The noncovalent character of GLP-1-Am oligomers was assessed by sodium dodecyl sulfate-polyacrylamide gel electrophoresis (SDS-PAGE) using a precast 4–12% Bis-Tris gel (Life Technologies, Invitrogen, USA) run in NuPAGE MES SDS running buffer (Life Technologies) for 35 min at a constant voltage of 200 V. Peptide samples were prepared in NuPAGE LDS sample buffer (Life Technologies), heated for 5 min to 95°C, and immediately loaded onto the gel. Protein and peptide standards, Mark 12 Unstained Standard and PageRuler Unstained Protein Ladder (both Thermo Fisher Scientific, USA), were run in parallel for size reference. Gels were stained with InstantBlue (Expedeon, United Kingdom).

Liquid chromatography-mass spectrometry

Liquid chromatography-mass spectrometry (LC-MS) analysis was performed on Waters' Xevo G2-S bench top QTOF (Waters Corporation, USA) using electrospray ionization in the positive mode. The peptide samples at around 10 μM concentration in 25 mM Tris buffer (pH 8.5) were run according to the LC method indicated in Table S7 with total ion detection.

RESULTS

GLP-1 and its C-terminally amidated analog both form amyloid-like fibrils at near neutral pH

As was shown in previous studies, GLP-1 forms amyloid fibrils over a wide range of conditions and the fibrillation rate

depends on pH (5–7). Here, the fibrillation propensity of GLP-1 and its C-terminally amidated variant, GLP-1-Am, was studied close to physiological pH values. Using ThT assays, the fibrillation of GLP-1-Am was monitored at pH 7 and 8 (Fig. 1 *A*). The fibrillation under these conditions was slow with lag times of ≥50 h and did not reach completion even after 6 days of incubation at 37°C with agitation. The change in fluorescence intensity (i.e., the slope of the ThT curve after the lag phase) may indicate a lower apparent growth rate at pH 7 compared with pH 8. However, the differences in the fluorescence intensity can also be caused by differences in fibril morphology or electrostatic interactions between the ThT dye and the fibril surface (37). Therefore, in this case, it is not possible to relate the ThT intensity directly to the mass of fibrils and therefore the actual growth rate of the fibrils formed without also using a complementary technique, e.g., liquid chromatography, that can measure the concentration of remaining monomer and oligomeric species in solution. The rate of fibrillation of GLP-1-Am at pH 8 (Fig. 1 *B*) did not show the classical dependence on peptide concentration observed for systems that follow a simple nucleation-propagation mechanism. At the end of the ThT assays, the GLP-1-Am samples at pH 7 and 8 were imaged by transmission electron microscopy (TEM) (Fig. 1, *C* and *D*). The presence of amyloid fibrils was observed in both samples; however, the fibrils did not form a dense network but rather isolated clusters (Fig. 1, *C* and *D*). The aggregation of GLP-1 at pH 7.5, 7.7, and 8.2 has been reported previously (7), but here the aggregation was also measured at pH 7 and 8 in 10 mM phosphate by ThT assay at 37°C over 6 days and no increase of ThT fluorescence was detected (Fig. S1).

GLP-1 and GLP-1-Am form stable, low-molecular-weight oligomers

The aggregation of GLP-1 and GLP-1-Am was also studied using SEC. GLP-1 and GLP-1-Am samples at concentrations of 43, 85, or 150 μM were incubated in an aqueous buffer at pH 7.0 or 8.0 at 37°C with agitation. At various time points, aliquots were taken from the aggregating reaction, filtered using a 0.22-μm pore size membrane filter and applied to a Superose 12 10/300 or a Superdex 75 10/300 size-exclusion column to determine the distribution of monomer and oligomers at each time point. The filtration step removed any amyloid fibrils formed, leaving only soluble monomer and oligomers in solution. For both GLP-1 and GLP-1-Am, oligomers with a larger hydrodynamic radius than the monomer were observed to form slowly during the incubation period and did not deplete even when there was substantial aggregation into amyloid fibrils (Fig. 2). For both peptides, the formation of the oligomeric species was accompanied by monomer depletion. However, it should be noted that, under some conditions, monomer depletion is also caused by fibrillation of the peptide and not just oligomer formation. The intensity of the peaks corresponding to oligomers increased during the incubation period and did not decrease even at time points where significant amyloid fibril formation had occurred, suggesting that these oligomers are not capable of further

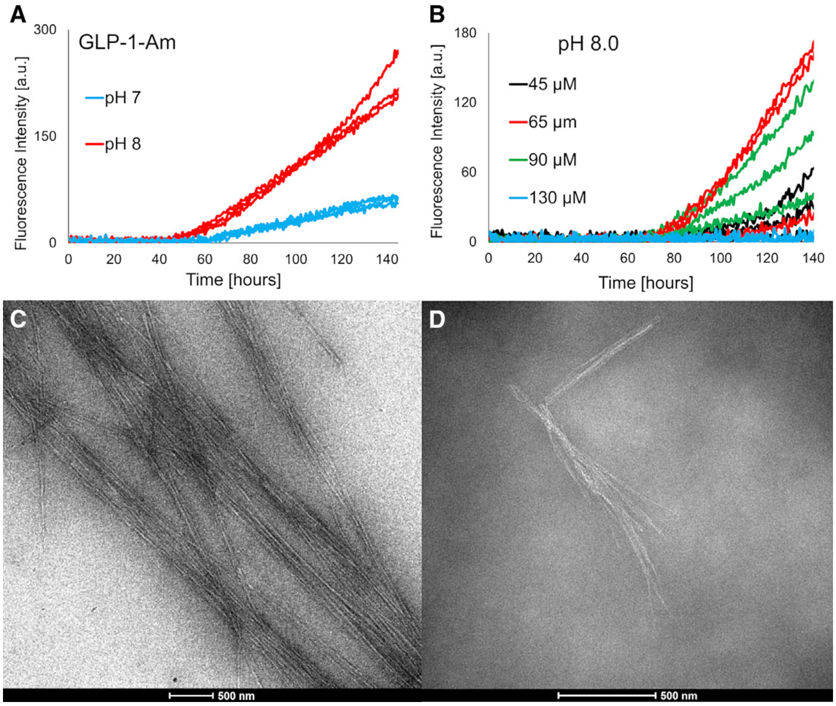

FIGURE 1 Fibrillation of GLP-1-Am at pH 7 and 8. The fibrillation of GLP-1-Am was monitored using a ThT assay (*A* and *B*). All conditions were performed in triplicate. (*A*) GLP-1-Am at 85 μM concentration was incubated at pH 7 (25 mM sodium phosphate, shown in *blue*), and pH 8 (25 mM sodium phosphate, shown in *red*), at 37°C with agitation over 6 days. (*B*) Fibrillation of GLP-1-Am in 25 mM phosphate at pH 8 at different peptide concentrations ranging from 45 to 130 μM was monitored over 6 days at 37°C. (*C* and *D*) Transmission electron microscopy images of GLP-1-Am fibrils at pH 7 and 8. GLP-1-Am samples at 85 μM concentration were imaged at the end point of the ThT assay (after a 6-day incubation at 37°C with agitation) in 25 mM phosphate at pH 7 (*C*) or 25 mM phosphate at pH 8 (*D*), scale bar 500 nm. To see this figure in color, go online.

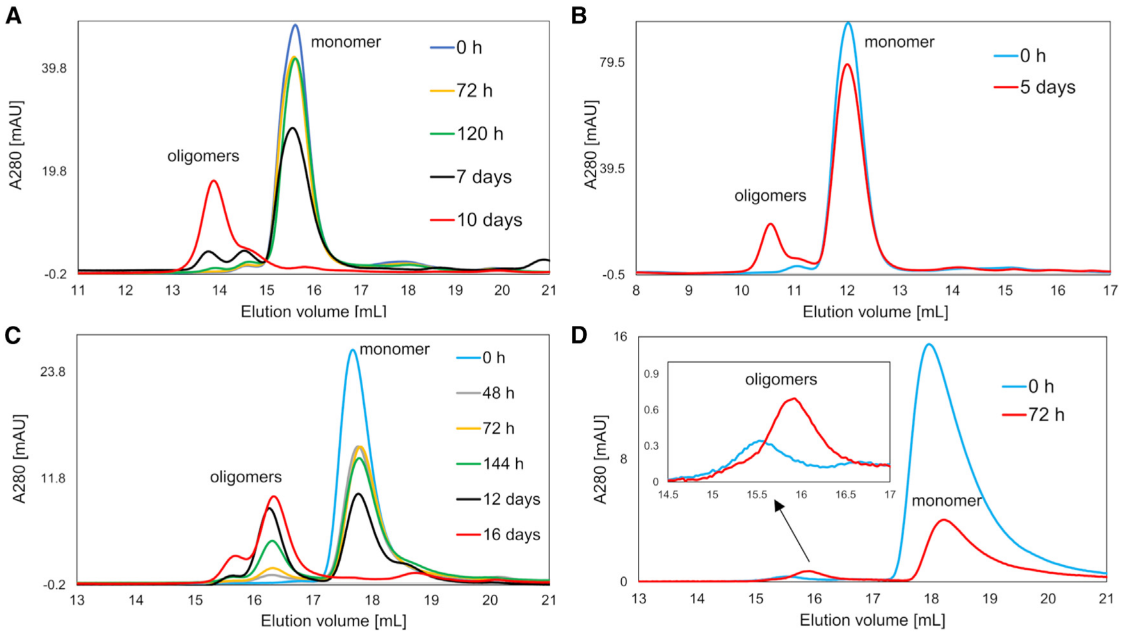

FIGURE 2 Formation of low-molecular-weight oligomers of GLP-1 and GLP-1-Am observed using size-exclusion chromatography (SEC). (*A*) SEC of a sample of 85 μM GLP-1 incubated in 10 mM sodium phosphate buffer at pH 7 at 37°C with 180 rpm agitation over 10 days. (*B*) SEC of a sample of 150 μM GLP-1 in 10 mM Tris buffer at pH 8 at time zero and after a 5-day incubation at 37°C with 180 rpm agitation. (*C*) SEC of a sample of 85 μM GLP-1-Am incubated in 25 mM sodium phosphate buffer at pH 8 at 37°C with 180 rpm agitation over 16 days. (*D*) SEC of a sample of 43 μM GLP-1-Am in 25 mM sodium phosphate buffer, pH 7, at time zero and after a 72-h incubation at 37°C with 180 rpm agitation. The samples were analyzed at different time points using Superose 12 10/300 (in *A*, *C*, and *D*) or Superdex 75 10/300 (in *B*) size-exclusion columns, where the elution buffer was the same as the incubation buffer for each sample. The difference in elution volumes of monomeric GLP-1 and GLP-1-Am is most likely due to their slightly different conformations and differences at the ionic strength and pH of elution buffers used in the experiments. To see this figure in color, go online.

aggregation/fibrillation or dissociation back into the monomeric state. After 10 days of incubation with shaking at 37°C for GLP-1 at pH 7 (Fig. 2 *A*) and 16 days of incubation and shaking at 37°C for GLP-1-Am at pH 8 (Fig. 2 *C*), the low-molecular-weight oligomers represented around 40% of the initial sample concentration, Table S1. A more detailed quantitative analysis of the relative amounts of oligomers formed during the incubation is given in Table S1 and Fig. S2.

The formation of these low-molecular-weight oligomers of GLP-1 and GLP-1-Am was shown to be reproducible in multiple buffers in the pH range 7–8.5, including sodium phosphate, Tris, or ammonium bicarbonate buffer (Figs. S3 and S4). Oligomerization of GLP-1-Am was reproducible for peptide batches supplied by different manufacturers (Fig. S5). For GLP-1, only one batch was used. Both GLP-1 and GLP-1-Am also show a higher tendency to form these oligomers in lower ionic strength buffers, i.e., 10 mM sodium phosphate buffer, or 10 and 25 mM Tris buffer, than at the buffers with higher ionic strengths (>100 mM) (Figs. S3 and S4).

The size of the observed oligomeric species was estimated using calibration curves of the two SEC columns, Superose 12 10/300 and Superdex 75 10/300 (see Figs. S6–S8). Based on the calibration curves, the size of the oligomers for GLP-1-Am was determined to be in the range from 6.7 to 13 kDa, approximately dimers to tetramers. For GLP-1, the size of oligomers was estimated to be in the range of trimers to pentamers.

In addition, the changes in oligomer distribution of GLP-1 and GLP-1-Am over time were studied using velocity sedimentation experiments (Beckman Optima XL-I Analytical Ultracentrifuge). Samples of 85 μM GLP-1 and GLP-1-Am were either freshly prepared before the measurement or incubated for 7 days at 37°C with 180 rpm agitation. The experiment was performed with centrifugation at 50,000 rpm and sedimentation curves were collected over approximately 24 h for each sample. The data were fitted to a continuous c(*s*) distribution model in a Sedfit program (38). In addition to a peak corresponding to the nonsedimented monomer with a sedimentation coefficient close to 0, both peptides formed low-molecular-weight oligomers, with a high content of dimer (over 40% in fresh samples) and lower amounts of larger oligomers (from 4-mer to 13-mer) (Fig. 3). The mass and relative content of the detected oligomeric species for both fresh and aged samples as determined by the Sedfit program are given in Table S5. In contrast to the SEC, during the sedimentation velocity experiment, the samples do not undergo dilution and do not interact with any column matrix. Therefore, oligomeric species that do not have sufficient stability to be detected by SEC (mainly oligomers formed in freshly prepared

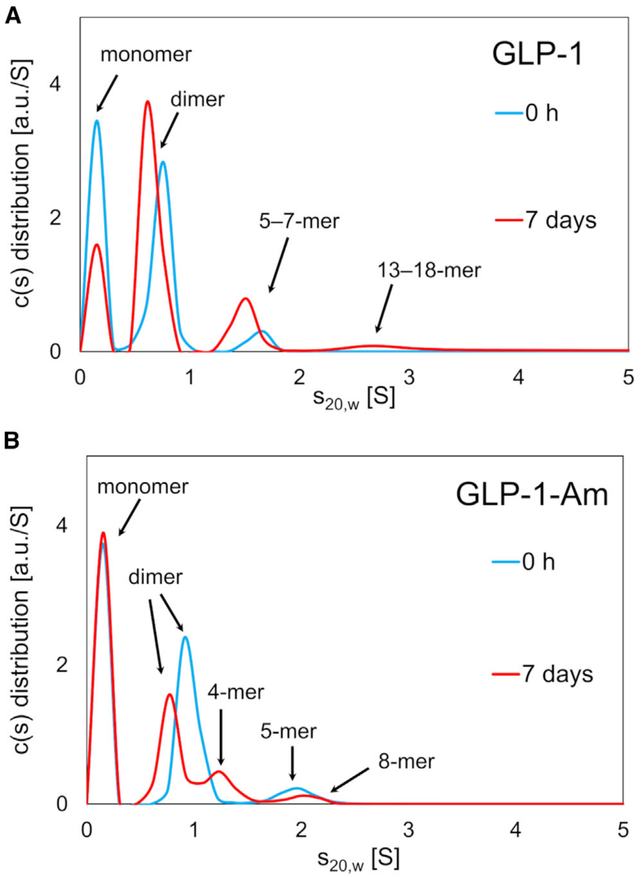

FIGURE 3 Formation of low-molecular-weight oligomers of GLP-1 and GLP-1-Am observed by sedimentation velocity. GLP-1 (*A*) and GLP-1-Am (*B*) at 85 μM were prepared in 25 mM phosphate at pH 8 before the experiment or incubated at 37°C with 180 rpm agitation for 7 days. After a 2-h temperature equilibration to 20°C, the experiment was performed with centrifugation at 50,000 rpm using Beckman Optima XL-I Analytical Ultracentrifuge. The interference sedimentation curves were collected as 300 scans (approximately 24 h run) and fitted to a continuous c(*s*) distribution model implemented in the Sedfit program. The sedimentation coefficient was corrected for the standard state of water at 20°C ($s_{20,w}$). The size of oligomeric species was calculated using the Sedfit program. To see this figure in color, go online.

samples) can be detected in sedimentation velocity experiments. Changes in oligomer distribution over time were apparent for both GLP-1 (Fig. 3 *A*) and GLP-1-Am (Fig. 3 *B*). GLP-1 showed changes in the ratio of monomeric and oligomeric peaks and GLP-1-Am formed new oligomeric species over time. Discrepancies in the amounts of oligomers detected by SEC (Table S1) and sedimentation velocity (Table S5) are likely to be due to the fact that less-stable, transient oligomers can be detected in sedimentation velocity experiments but not SEC; e.g., two types of dimers may be present in the sample—a less-stable, transient dimer, which is detected only by sedimentation velocity, and a stable dimer, which can be detected in both sedimentation velocity and SEC experiments. It should also be noted that the two techniques are performed over different timescales—the SEC (approximately 30 min) and sedimentation velocity (approximately 24 h)—and this may also contribute to the differences observed.

Structural characterization of the low-molecular-weight oligomers

GLP-1-Am samples were characterized before and during incubation at pH 8 at 37°C with continuous agitation using a number of biophysical techniques (Fig. 4). Spectra of non-aggregated (i.e., largely monomeric) peptides were recorded immediately after dissolving the lyophilized peptide powder. Low-molecular-weight oligomers were either separated using SEC or obtained after filtering out the fibrils from the aggregated sample using a 0.22-μm membrane, and their spectra were recorded. Finally, a fibrillar sample was obtained by centrifugation, and the resultant pellet was resuspended in buffer and its spectra then acquired.

For monomeric GLP-1-Am, the far-UV CD spectrum showed a contribution from α-helical and β structure as well as disordered regions (Fig. 4 *A*). After a 9-day incubation, significant changes appeared in the spectrum caused by the formation of low-molecular-weight oligomers and amyloid fibrils (Fig. 4 *A*). The far-UV CD spectra of isolated oligomeric and fibrillar species (Fig. 4 *A*, *bottom*) formed by GLP-1-Am showed a prevalence of disordered regions for the low-molecular-weight oligomers and a high percentage of β sheet content for the amyloid fibrils (Table S6).

Fourier-transform IR absorption (FT-IR) and VCD spectroscopy were also used to characterize the GLP-1-Am sample after aggregation. IR spectra of peptides and proteins are dominated by the amide I signal (mainly carbonyl C=O stretch) and they are routinely used for secondary-structure determination. In VCD, which is defined as the difference in the absorbance of left- and right-circularly polarized IR radiation, the stereoselectivity of CD complements the structural specificity of IR. Moreover, VCD is nowadays increasingly employed in aggregation studies due to the signal enhancement and splitting often observed with amyloid fibrils. It mainly occurs in the amide I region and the enhancement is induced by a highly regular cross-β sheet structure (39–43). VCD can, therefore, be used for monitoring the fibrillation process and structural characterization. Based on the sign pattern of the enhanced spectra, the different morphologies of fibrils or the overall organization of the macro-assembly can be distinguished (44–48). In this study, both FT-IR and VCD spectra were simultaneously recorded for the sample in a deuterated buffer of a corresponding pD. Fig. 4 *B* shows the spectra of samples of GLP-1-Am aggregated/fibrillated under basic conditions after 8 days of incubation in 25 mM deuterated sodium phosphate buffer at pD 8. FT-IR and VCD spectra of the sample incubated under acidic conditions in 25 mM phosphate at pD 3, where fibrils are formed much faster, are shown

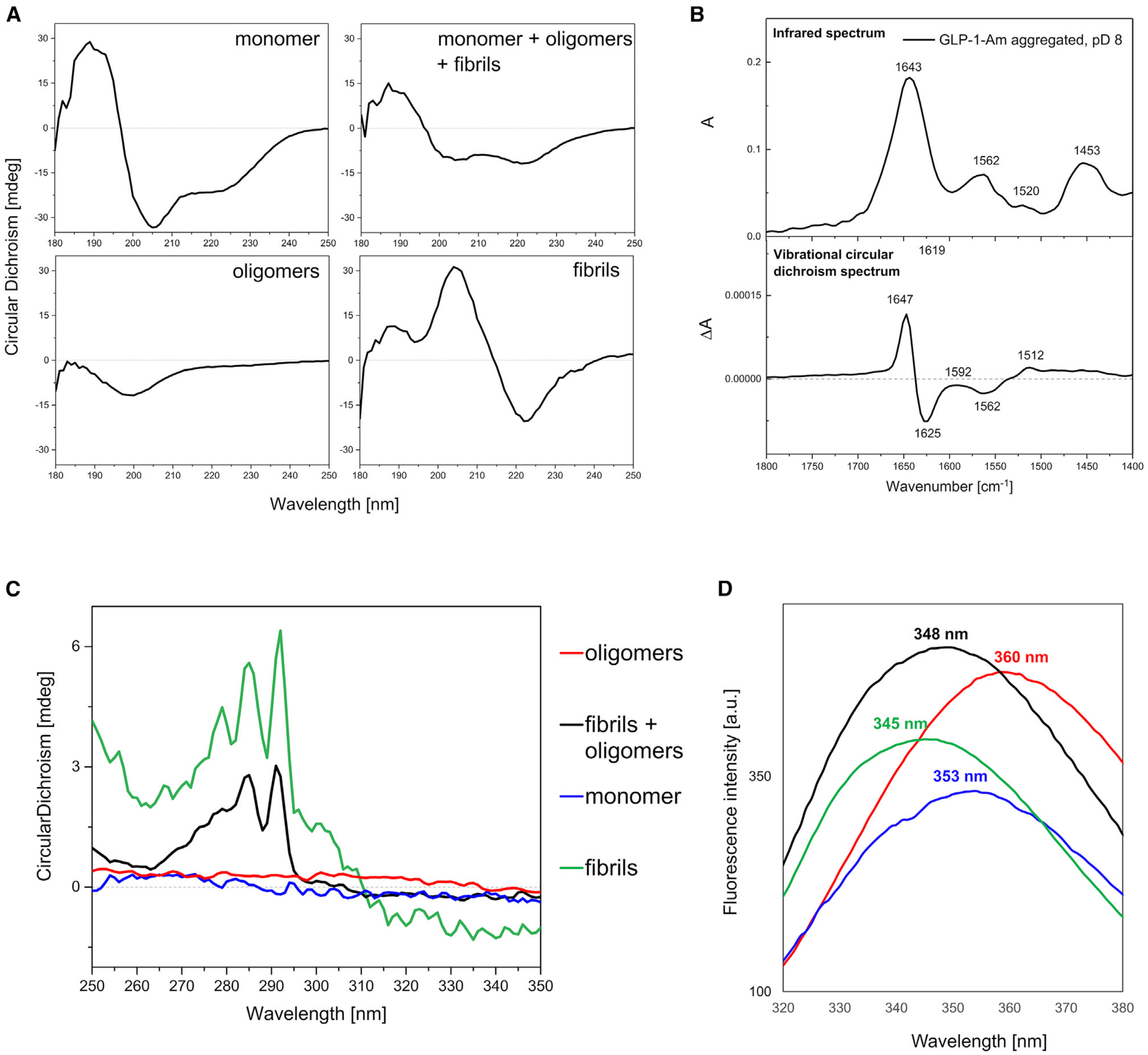

FIGURE 4 Structural characterization of the isolated low-molecular-weight oligomers and fibrils of GLP-1-Am. (*A*) Far-UV CD spectra of different species present in the aggregating mixture of 85 μM GLP-1-Am in 25 mM sodium phosphate buffer at pH 8. The top left panel shows the spectrum of the largely monomeric solution recorded directly after preparation of a fresh sample of GLP-1-Am from lyophilized powder. The top right spectrum was recorded after 9 days of incubation at 37°C with agitation. The spectrum of low-molecular-weight oligomers (*bottom left*) was recorded after their separation from the aggregating mixture using SEC and the spectrum of fibrils (*bottom right*) was recorded after their separation from the mixture using centrifugation. (*B*) FT-IR and VCD spectra of aggregated GLP-1-Am at pD 8. The sample of 1.2 mM GLP-1-Am in 25 mM deuterated sodium phosphate buffer at pD 8 was incubated for 8 days at 37°C with 180 rpm agitation. (*C*) Near-UV CD spectra of the species present in the aggregating mixture of GLP-1-Am (85 μM) after a 9-day incubation at pH 8 in 25 mM sodium phosphate buffer. Samples for the near-UV CD were prepared by the same procedure as the samples for far-UV CD. (*D*) Fluorescence emission spectra showing the maxima (λ_{max}) of the intrinsic tryptophan fluorescence of GLP-1-Am monomer, oligomers, and fibrils. All samples were measured in 25 mM sodium phosphate buffer at pH 8. Samples for the intrinsic tryptophan fluorescence emission measurement were prepared by the same procedure as those for CD. To see this figure in color, go online.

in Fig. S9 *A* for comparison. In the FT-IR spectrum at pD 8, the broad amide I band with a maximum at 1643 cm^{-1} corresponds to a disordered structure. In contrast, an enhanced VCD signal was observed ($\Delta A/A \approx 10^{-3}$, at approximately 1647 cm^{-1}), indicating the formation of amyloid fibrils. These, seemingly contradictory results, can be explained by the presence of multiple species in the sample—amyloid fibrils and low-molecular-weight oligomers. Contributions from both are visible in the resulting IR spectrum, while the VCD spectrum is dominated by the fibrils as the signal from the low-molecular-weight oligomers is expected to be weak ($\Delta A/A \sim 10^{-4}$ to 10^{-5}, in the amide I maximum). The low-molecular-weight oligomers are likely to be in excess of the

fibrils under these conditions since their disordered conformation dominates the FT-IR spectrum. A more characteristic "amyloid-like fibril" FT-IR (amide I signal split, 1612/1681 cm^{-1}) and VCD spectrum (even higher intensity between approximately 1590 and 1687 cm^{-1}) was measured at pD 3 (Fig. S9 *A*). Thus, low pD favors a higher content of β structured amyloid fibrils (and fewer or no low-molecular-weight oligomers) compared with pD 8. The presence of a dense network of amyloid fibrils was also confirmed at pD/pH 3, which was also confirmed using TEM (Fig. S9 *B*).

Near-UV CD spectra and intrinsic tryptophan fluorescence spectra of GLP-1-Am were acquired to gain more information on the local environment of the single tryptophan in GLP-1-Am, Trp25 (Fig. 4 *C* and *D*). Samples of largely monomeric peptide, low-molecular-weight oligomers and fibrils were prepared in the same way as described for the far-UV CD measurements. From the near-UV CD spectrum, it is apparent that, in the amyloid fibril structure, the Trp25 side chain is fixed in its local environment as indicated by the chiral absorption maximum at around 290 nm. In the monomer and low-molecular-weight oligomers, the Trp25 residue side chain is exposed to the solvent and less rigid. Therefore, it loses its CD signal in the near-UV CD region (Fig. 4 *C*). These observations are supported by the intrinsic tryptophan fluorescence emission spectra, which show fluorescence emission maxima (λ_{max}) at 345 and 360 nm for the fibrils and the low-molecular-weight oligomers, respectively (Fig. 4 *D*). The lower wavelength of λ_{max} in fibrils indicates a greater extent of Trp25 burial, i.e., Trp25 is at least partially buried in the fibril structure; however, in the low-molecular-weight oligomers the side chain is exposed to the aqueous solvent. Interestingly, Trp25 in the monomer has a slightly lower λ_{max} than in the low-molecular-weight oligomers (353 vs. 360 nm), suggesting that the Trp side chain is more solvated in the oligomers than the monomer, consistent with the observations from the far-UV CD spectrum that the peptide is largely disordered under these conditions (Fig. 4 *A*).

A similar characterization and analysis was performed on the low-molecular-weight oligomers of GLP-1, without the FT-IR and VCD experiments. The far-UV CD and intrinsic tryptophan fluorescence spectra of the low-molecular-weight oligomers of GLP-1 are shown in Fig. S10 and indicate extensive disordered structure, similar to the oligomers of GLP-1-Am. In addition, the low-molecular-weight oligomers of both GLP-1 and GLP-1-Am (in contrast to the monomeric peptide) exhibit light scattering revealed by an increased absorbance over the range 310–350 nm in the UV-vis absorption spectra, a further indication of aggregation (Fig. S11) (49).

Membrane disruption experiments

The membrane disruption potential of low-molecular-weight oligomers of GLP-1-Am was tested using a calcein-release assay from DOPS vesicles at pH 8 (Fig. S12). The assay was performed with 4% (w/w) of preformed isolated GLP-1-Am oligomer and no release of calcein from vesicles was detected over the timescale of the assay. This observation suggests that these oligomers are not capable of disruption of the liposome membrane.

Stability of low-molecular-weight oligomers and their effect on fibrillation kinetics

The stability of the low-molecular-weight oligomers of GLP-1 and GLP-1-Am was investigated using a number of different approaches. First, they were shown to be stable after their separation by SEC and subsequent reinjection onto the SEC column (Fig. S13). Surprisingly, they were also stable with respect to agitation, temperature changes, or upon addition of a low percentage of isopropanol (Fig. S14). The stability of these unstructured oligomers, in addition to their slow rate of formation, suggests that they might be stabilized by covalent links. Two methods were used to investigate this hypothesis. The covalent/noncovalent character of GLP-1-Am oligomers was assessed using SDS-PAGE and LC-MS (Fig. 5). The SDS-PAGE of the low-molecular-weight oligomers showed only a single band corresponding to the molecular weight of the peptide monomer (Fig. 5 *A*). The LC-MS analysis, using electrospray ionization in the positive mode, showed the MS spectrum of GLP-1-Am oligomers to be identical to the spectrum of the GLP-1-Am monomer (Figs. S15 and S16). Moreover, the isotopic distribution of the peaks in the oligomeric GLP-1-Am spectrum was consistent with the isotopic distribution of the peaks in the MS spectrum of monomeric GLP-1-Am. Therefore, neither the SDS-PAGE nor the LC-MS analysis of GLP-1-Am oligomers identified any covalent links between peptide chains leading to oligomer formation that might have occurred during the prolonged incubation at 37°C.

Due to the slow formation and high stability of the observed oligomers, SEC and sedimentation velocity experiments were also performed with GLP-1-Am containing a β-aspartic acid at position 9 to exclude the possibility that the observed species originate from isomerization of aspartic acid in the peptide. The isomerization of Asp9 did not have any effect on the elution volume of the monomer and only a minor effect was observed in the sedimentation velocity experiment compared with a freshly prepared sample of GLP-1-Am (Fig. S17). Therefore, the observed oligomer species are not likely to result from isomerization of Asp9. Other chemical modifications that may be induced by prolonged incubation at 37°C are deamidation and/or isomerization of Gln17. Although glutamine deamidation occurs with extremely slow rates (half-lives ranging from 600 to 20,000 days) (50–52), the possibility of deamidation in the aged sample of GLP-1-Am was tested using an isoelectric focusing gel. As shown in Fig. S18, 85 μM

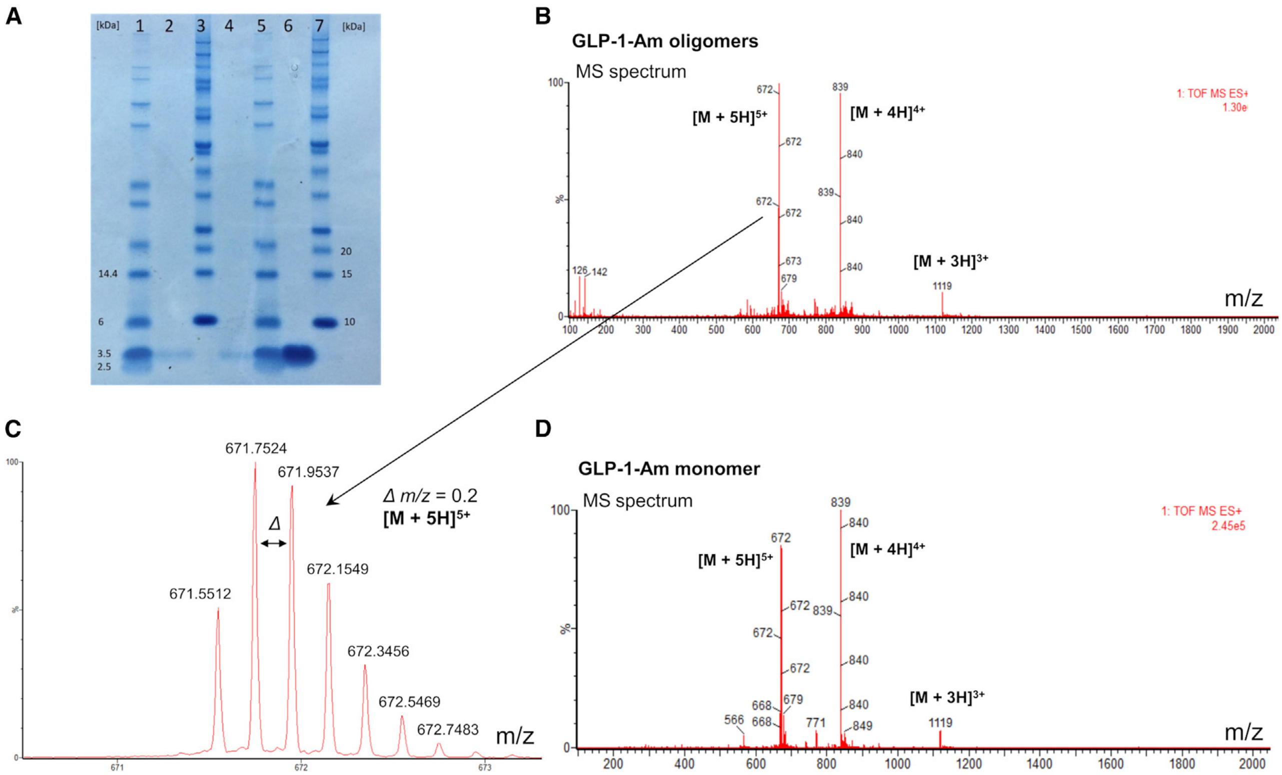

FIGURE 5 Denaturing gel electrophoresis (SDS-PAGE) and LC-MS showing the noncovalent character of the isolated low-molecular-weight oligomers of GLP-1-Am. (*A*) SDS-PAGE analysis of GLP-1-Am oligomers formed at pH 8 in 25 mM sodium phosphate buffer. The oligomers were isolated (by SEC) from a 130 μM GLP-1-Am mixture incubated in 25 mM sodium phosphate buffer at pH 7.5 for 5 days at 37°C with continuous shaking at 180 rpm. Lanes 2 and 4: GLP-1-Am oligomers; lane 6: fresh (monomeric) GLP-1-Am sample. Lanes 1, 3, 5, and 7: proteins reference ladders. (*B* and *C*) Mass spectrum (using electrospray ionization in the positive mode) of oligomeric GLP-1-Am isolated using SEC and a detail of the isotopic distribution of peak with $m/z = 672$. (*D*) Mass spectrum of monomeric GLP-1-Am using a freshly prepared sample. To see this figure in color, go online.

GLP-1-Am incubated in 25 mM phosphate at pH 8 for 8 days shows a single band corresponding to the pI of a freshly prepared sample of GLP-1-Am. Therefore, the observed oligomeric species are not likely to be formed as a result of deamidation.

To assess the effect of these low-molecular-weight oligomers on the fibrillation kinetics, an additional ThT assay was performed. In this case, preformed purified oligomers were added at the start of the reaction using two different concentrations of monomeric GLP-1-Am (Fig. S19). The addition of preformed oligomers was shown to have a slight inhibitory effect on the fibrillation process, affecting the apparent growth phase and/or the lag time of the reaction (Fig. S19). These results suggest that the oligomers may inhibit/slow down secondary nucleation processes and/or fibril elongation.

DISCUSSION

GLP-1 is an important therapeutic peptide drug and its derivatives are widely used in the treatment of type 2 diabetes (3,4,53–57). Nevertheless, due to its intrinsic physical instability in vitro, its manufacturing and formulation are very challenging. As reported previously, GLP-1 forms amyloid fibrils under a wide range of conditions with differing fibrillation rates, which depend critically on pH and peptide concentration (7,58). Under some conditions, there is evidence that GLP-1 not only forms on-pathway oligomers, which can convert directly into amyloid fibril, but that it can also populate oligomeric species that are off-pathway products, and which thus reduce the rate of formation of amyloid fibrils (7). Here, we show that a C-terminally amidated variant, GLP-1-Am, also fibrillates under similar conditions to GLP-1 and also populates off-pathway oligomers, as shown by the fact that the lag time associated with its aggregation kinetics does not vary with peptide concentration at pH 8 (Fig. 1 *B*). The main focus of this investigation was to isolate and characterize the putative off-pathway oligomers for GLP-1 and GLP-1-Am, to understand their structures, why they form, and how they affect the self-assembly process. SEC was used to detect and isolate oligomeric species, and a variety of spectroscopic methods (UV-vis, far-UV and near-UV CD, FT-IR, VCD, and intrinsic tryptophan fluorescence) were employed to structurally characterize the oligomers and SEC employed to assess their stability. The noncovalent

character of the oligomers in question was demonstrated using LC-MS and SDS-PAGE.

GLP-1 and GLP-1-Am form oligomeric species that compete with fibrillation

Here, we report the formation of low-molecular-weight oligomers of both GLP-1 and GLP-1-Am that are observed in the pH range 7–8.5. Using SEC, distinct well-resolved oligomeric peaks were detected after a prolonged incubation at 37°C in different aqueous buffers (Figs. 2, S3, and S4). The formation of low-molecular-weight oligomers was reproducible for both GLP-1 and GLP-1-Am. In addition, for GLP-1-Am these results were reproduced for two different peptide batches produced by different manufacturers (Fig. S5), and for GLP-1 only one batch of the peptide was used. The formation of these low-molecular-weight oligomers during incubation at 37°C was also confirmed by sedimentation velocity experiments (Fig. 3). These results give us confidence that the formation of low-molecular-weight oligomers of GLP-1 and GLP-1-Am is highly reproducible under the conditions described. The increase in the intensity of the oligomeric peaks occurs over days showing that their rate of formation is rather slow, indicating that this oligomerization process is not self-propagated/catalyzed, in contrast to fibrillation (59). Importantly, these oligomers remained in solution even after depletion of the peptide monomer under conditions where a considerable amount of peptide was converted into a fibrillar species (Fig. 2). This suggests that the oligomers are not easily capable of further aggregation into amyloid fibrils or of dissociation back into monomers. Most likely, they represent a stable form of aggregate and, therefore, their formation competes with fibrillation.

GLP-1 and GLP-1-Am form oligomers ranging from dimers to pentamers

Using a set of protein calibration standards on two size-exclusion columns, Superose 12 10/300 and Superdex 75 10/300, the size of the low-molecular-weight oligomers of GLP-1-Am was estimated to be in the range of dimers to tetramers, while the GLP-1 oligomers were estimated to be in the size range from trimers to pentamers (Fig. 2). For small peptides, such as GLP-1, it is beyond the scope of this technique to determine the size more precisely because the calibration of the size-exclusion column is based on the separation of globular proteins according to their hydrodynamic radii. This could cause inaccuracies in the size estimates since GLP-1 and GLP-1-Am monomers and oligomers are unlikely to be fully globular (spherical). The presence of GLP-1 and GLP-1-Am oligomers from a size range of dimers to pentamers (with a small amount of larger species) was also confirmed in sedimentation velocity experiments for aged samples (Fig. 3).

Oligomers of GLP-1 and GLP-1-Am have highly disordered structures

As shown by multiple spectroscopic techniques, the low-molecular-weight oligomers of both GLP-1 and GLP-1-Am show a high level of disordered structure. Far-UV CD spectra reveal that largely monomeric solutions of GLP-1 and GLP-1-Am both have regions of α-helical and β structure, whereas the low-molecular-weight oligomeric species show significantly less of these secondary structure motifs, and a prevalence of disordered regions. Near-UV CD, VCD, and intrinsic tryptophan fluorescence emission spectra support these observations and indicate that the local environment of Trp25 in the oligomers is highly exposed to the solvent and freely rotating. Highly disordered oligomers have also been reported in the aggregation of Aβ40 and Aβ42 (60), transthyretin (61), and α-synuclein (62,63). Some studies on the cytotoxicity of α-synuclein (63) and Aβ40 (64) oligomers suggested that higher structural complexity and a high content of β sheet is needed to disrupt the cell membrane. Indeed, the highly disordered low-molecular-weight oligomers described in this study did not show any membrane disruption potential when assessed using calcein-release assay with DOPS vesicles (Fig. S12). However, as reported elsewhere (14,65), the secondary structure of an oligomer cannot always be linked with cytotoxicity without experimental assessment, since the presence of hydrophobic patches on the surface of an oligomer may induce cytotoxicity.

The low-molecular-weight oligomers of GLP-1 and GLP-1-Am are stable with respect to many stressors

The fact that the low-molecular-weight oligomers of both GLP-1 and GLP-1-Am were detected as fully resolved peaks in SEC experiments highlights their stability, as they do not dissociate during the 10-fold dilution of the sample as it runs through the SEC column. Note that detection of peptide or protein oligomers using SEC is not always possible without oligomers first being cross-linked to enhance their stability. However, there are other examples of noncovalent and noncross-linked oligomers of self-assembly-prone peptides and proteins that have been detected and isolated using SEC, most notably α-synuclein (62), transthyretin (61), SOD1 (66), prion protein (67), and amyloid-β peptide (28,29,60). Perhaps more unusual is the high stability of the low-molecular-weight oligomers of GLP-1 and GLP-1-Am with respect to time, agitation, heating, or even low percentages of organic solvents (Fig. S14). These results, along with the timescales on which these oligomers are formed (1–15 days), suggest that the stability might be a direct result of a covalent modification of the peptide resulting in essentially cross-linked species. GLP-1 does not contain any cysteines, therefore disulfide

bridges cannot form, but potential dimerization might have occurred through tyrosine or tryptophan side chains, although this is less likely. Nevertheless, LC-MS did not detect any chemical modification of this type within the oligomerized peptide. Also, the SDS-PAGE experiment showed that the band from the oligomeric sample was identical to the band of the monomeric peptide. It is, therefore, highly likely that, despite the unusual stability and disordered conformation of the low-molecular-weight oligomers of GLP-1 and GLP-1-Am, they are noncovalent in character. Noncovalent oligomers of a similar stability with respect to time and dilution have been observed previously for the amyloid-β peptide (28), SOD1 (66), cystatin (68,69), and β2-microglobulin where Cu^{2+} ions drive oligomer formation but are not essential for its stability (25).

Given these results, it is interesting to speculate on the noncovalent forces that maintain the peptides in their oligomeric state. Although the hydrophobic effect is frequently of importance in self-assembly reactions, there is no burial of the hydrophobic side chain of Trp25 in the oligomers of GLP-1 and GLP-1-Am, suggesting that it may not play a dominant role here (of course, we cannot exclude the burial of other hydrophobic sides chains in the peptide). In contrast, there is some evidence that electrostatic interactions play a role, as demonstrated by the fact that the oligomers form preferentially in low-ionic-strength buffers. The fact that very disordered polypeptide chains can form complexes with high stability has been shown recently in the case of the C-terminal tails of histone H1 (70,71). These results further establish that fixed structure is not essential for maintaining a complex—in the case of H1 a hetero-oligomer with the nuclear chaperone prothymosin-α, in the case of GLP-1 and GLP-1-Am, described here, homo-oligomers.

Low-molecular-weight oligomers of GLP-1 and GLP-1-Am are likely off-pathway states

We have shown that the isolated oligomers of GLP-1 and GLP-1-Am are very resistant to further association into fibrils, suggesting that they are not intermediates en route to fibrils, i.e., on-pathway oligomers, but they are rather off-pathway species formed by a process in competition with fibrillation. Moreover, as shown in the ThT assay with preformed low-molecular-weight oligomers of GLP-1-Am, the addition of oligomers slightly shows down the fibrillation kinetics (Fig. S19). A similar inhibitory effect on amyloid formation has recently been reported for D76N-β2-microglobulin dimers (72). This, in part, helps to rationalize the unusual dependence that has been observed between aggregation lag times and peptide concentration for GLP-1, where under specific conditions the lag times increase with increasing GLP-1 concentration (7). For such unusual kinetics, a mechanism containing a unimolecular step has been proposed that can explain the inverse concentration-fibrillation trend (33). In this mechanism, the original "fibrillation-inactive" monomer has to convert into a "fibrillation-active" monomer, which can further form on-pathway oligomers and amyloid fibrils (7,33). Fibrillation-inactive monomers have also been proposed to inhibit/slow the fibrillation process by interacting with on-pathway oligomers (33). We hypothesize that the observed stable low-molecular-weight oligomers of GLP-1 and GLP-1-Am are either formed by fibrillation-inactive monomer self-assembly or by the conversion of on-pathway oligomers upon interaction with the fibrillation-inactive monomer or by a combination of these two mechanisms (Fig. 6 *A*). As was suggested by the ThT assay with preformed isolated oligomers (Fig. S19), the low-molecular-weight oligomers may also partly inhibit the secondary

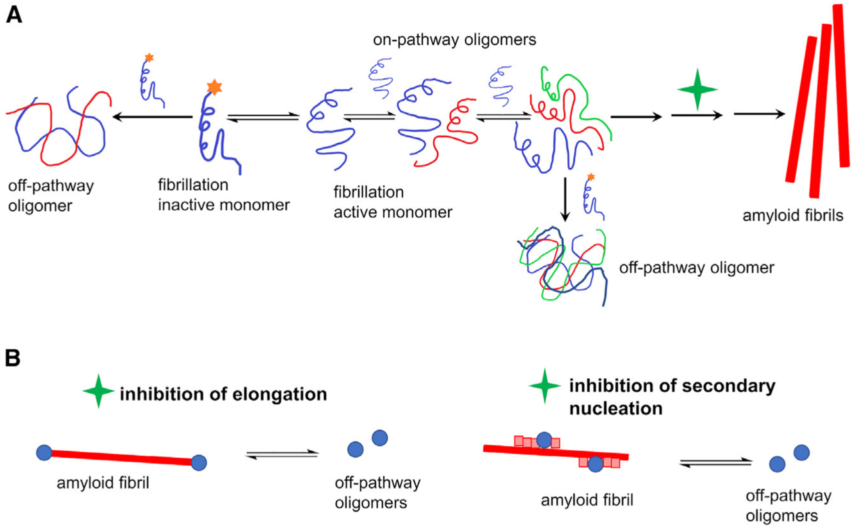

FIGURE 6 Possible mechanisms for the formation of off-pathway low-molecular-weight oligomers and their inhibition of secondary nucleation processes and fibrillation. "Fibrillation-inactive" monomers (indicated by a star) either self-assemble into an off-pathway oligomer or convert into the "fibrillation-active" monomer in a unimolecular step and/or the off-pathway oligomer is formed upon interaction of the on-pathway oligomer with the fibrillation-inactive monomer (*A*). The green star in scheme (*A*) refers to the processes illustrated in (*B*). Off-pathway oligomers may also reversibly inhibit elongation and/or secondary nucleation processes and slow the overall kinetics of amyloid formation (*B*). To see this figure in color, go online.

fibrillation processes and therefore slow the formation of amyloid fibrils (Fig. 6 *B*).

Our observations are also partly consistent with the peptide micelle formation hypothesis (73), since the surfactant-like behavior of a peptide and micelle formation above a certain concentration threshold can also lead to inhibition of fibrillation (74,75).

CONCLUSIONS

In this study, stable low-molecular-weight oligomers of the therapeutic peptide hormone GLP-1 and its C-terminally amidated analog, GLP-1-Am, were detected and characterized. GLP-1 and GLP-1-Am, which are prone to amyloid fibril formation over a wide range of conditions, both form these low-molecular-weight oligomers at pH values between 7 and 8. These oligomers are not easily capable of further self-assembly into larger oligomers or amyloid fibrils or dissociation back into monomers. Moreover, the addition of preformed oligomers to an aggregation reaction of monomer slightly slows down the fibrillation kinetics. Therefore, they are likely products of an alternative self-assembly pathway that competes with fibril formation. The size of the oligomers was estimated to be from dimer to pentamer, and they were also shown to have a highly disordered structure. Surprisingly, these oligomers are stable with respect to time, temperature, and even a low percentage of isopropanol. However, neither SDS-PAGE nor LC-MS showed any covalent modification of the monomeric peptide within the oligomer. This uncommon observation of stable oligomeric species which are off-pathway to the fibrillation process further illustrates the diversity and complexity of the aggregation landscape of peptides in which on-pathway oligomers, protofibrils, and fibrils are not the only stable detectable species. It also sheds some light on the unusual dependence of GLP-1 aggregation kinetics on peptide concentration, which has been observed previously under some conditions (7). We believe that the observation and characterization of these species is of great importance since GLP-1 and its analogs are used as therapeutic drugs. Therefore, a careful analysis of all (even non-covalent) species formed during drug manufacturing and storage is essential.

SUPPORTING MATERIAL

Supporting material can be found online at https://doi.org/10.1016/j.bpj.2023.04.027.

AUTHOR CONTRIBUTIONS

E.P.B. and S.E.J. designed the study. E.P.B. performed the experiments and data analysis. M.K. and P.B. contributed with the FT-IR and VCD analysis. E.P.B. wrote the manuscript. All authors reviewed and edited the manuscript. All authors read and approved the final manuscript.

ACKNOWLEDGMENTS

E.P.B. was supported by a PhD studentship from Peterhouse, Cambridge. Bachem batch of GLP-1-Am was kindly provided by AstraZeneca UK; support also came from the Grant Agency of the Czech Republic (to P.B., 22-33060S). We would also like to thank Katie Gibson for her help with the LC-MS experiment, Dr. Heather Greer for her assistance with TEM, Dr. Katherine Stott for assistance with analytical ultracentrifugation, and Greta Musteikyte for providing the protocol and liposomes for calcein-release assay.

DECLARATION OF INTERESTS

V.L. and A.G.S. are employees of AstraZeneca.

REFERENCES

1. Drucker, D. J. 2006. The biology of incretin hormones. *Cell Metab.* 3:153–165. https://doi.org/10.1016/j.cmet.2006.01.004.
2. Holst, J. J. 2007. The physiology of glucagon-like peptide 1. *Physiol. Rev.* 87:1409–1439. https://doi.org/10.1152/physrev.00034.2006.
3. Yu, M., M. M. Benjamin, …, A. Schwendeman. 2018. Battle of GLP-1 delivery technologies. *Adv. Drug Deliv. Rev.* 130:113–130. https://doi.org/10.1016/j.addr.2018.07.009.
4. Fosgerau, K., and T. Hoffmann. 2015. Peptide therapeutics: current status and future directions. *Drug Discov. Today.* 20:122–128. https://doi.org/10.1016/j.drudis.2014.10.003.
5. Poon, S., N. R. Birkett, …, J. Zurdo. 2009. Amyloidogenicity and aggregate cytotoxicity of human glucagon-like peptide-1 (hGLP-1). *Protein Pept. Lett.* 16:1548–1556. https://doi.org/10.2174/092986609789839232.
6. Jha, N. N., A. Anoop, …, S. K. Maji. 2013. Characterization of amyloid formation by glucagon-like peptides: role of basic residues in heparin-mediated aggregation. *Biochemistry.* 52:8800–8810. https://doi.org/10.1021/bi401398k.
7. Zapadka, K. L., F. J. Becher, …, S. E. Jackson. 2016. A pH-induced switch in human glucagon-like peptide-1 aggregation kinetics. *J. Am. Chem. Soc.* 138:16259–16265. https://doi.org/10.1021/jacs.6b05025.
8. Ferrone, F. 1999. Analysis of protein aggregation kinetics. *Methods Enzymol.* 309:256–274. https://doi.org/10.1016/S0076-6879(99)09019-9.
9. Knowles, T. P. J., C. A. Waudby, …, C. M. Dobson. 2009. An analytical solution to the kinetics of breakable filament assembly. *Science.* 326:1533–1537. https://doi.org/10.1126/science.1178250.
10. Morris, A. M., M. A. Watzky, and R. G. Finke. 2009. Protein aggregation kinetics, mechanism, and curve-fitting: a review of the literature. *Biochim. Biophys. Acta.* 1794:375–397. https://doi.org/10.1016/j.bbapap.2008.10.016.
11. Arosio, P., T. P. J. Knowles, and S. Linse. 2015. On the lag phase in amyloid fibril formation. *Phys. Chem. Chem. Phys.* 17:7606–7618. https://doi.org/10.1039/C4CP05563B.
12. Chiti, F., and C. M. Dobson. 2017. Protein misfolding, amyloid formation, and human disease: a summary of progress over the last decade. *Annu. Rev. Biochem.* 86:27–68. https://doi.org/10.1146/annurev-biochem-061516-045115.
13. Cohen, S. I. A., S. Linse, …, T. P. J. Knowles. 2013. Proliferation of amyloid- 42 aggregates occurs through a secondary nucleation mechanism. *Proc. Natl. Acad. Sci. USA.* 110:9758–9763. https://doi.org/10.1073/pnas.1218402110.
14. Vivoli Vega, M., R. Cascella, …, F. Chiti. 2019. The toxicity of misfolded protein oligomers is independent of their secondary structure. *ACS Chem. Biol.* 14:1593–1600. https://doi.org/10.1021/acschembio.9b00324.
15. Mannini, B., E. Mulvihill, …, F. Chiti. 2014. Toxicity of protein oligomers is rationalized by a function combining size and surface

hydrophobicity. *ACS Chem. Biol.* 9:2309–2317. https://doi.org/10.1021/cb500505m.

16. Campioni, S., B. Mannini, …, F. Chiti. 2010. A causative link between the structure of aberrant protein oligomers and their toxicity. *Nat. Chem. Biol.* 6:140–147. https://doi.org/10.1038/nchembio.283.
17. Ahmed, M., J. Davis, …, S. O. Smith. 2010. Structural conversion of neurotoxic amyloid-B 1-42 oligomers to fibrils. *Nat. Struct. Mol. Biol.* 17:561–567. https://doi.org/10.1038/nsmb.1799.
18. Dear, A. J., T. C. T. Michaels, …, T. P. J. Knowles. 2020. Kinetic diversity of amyloid oligomers. *Proc. Natl. Acad. Sci. USA.* 117:12087–12094. https://doi.org/10.1073/pnas.1922267117.
19. Dear, A. J., G. Meisl, …, T. P. J. Knowles. 2020. Identification of on- and off-pathway oligomers in amyloid fibril formation. *Chem. Sci.* 11:6236–6247. https://doi.org/10.1039/C9SC06501F.
20. Eichner, T., and S. E. Radford. 2011. A diversity of assembly mechanisms of a generic amyloid fold. *Mol. Cell.* 43:8–18. https://doi.org/10.1016/j.molcel.2011.05.012.
21. Yoo, J. M., Y. Lin, …, Y. H. Lee. 2022. Polymorphism in alpha-synuclein oligomers and its implications in toxicity under disease conditions. *Front. Mol. Biosci.* 9:959425–959510. https://doi.org/10.3389/fmolb.2022.959425.
22. Niyangoda, C., J. Barton, …, M. Muschol. 2021. Origin, toxicity and characteristics of two amyloid oligomer polymorphs. *RSC Chem. Biol.* 2:1631–1642. https://doi.org/10.1039/d1cb00081k.
23. Kjaergaard, M., A. J. Dear, …, D. Klenerman. 2018. Oligomer diversity during the aggregation of the repeat region of tau. *ACS Chem. Neurosci.* 9:3060–3071. https://doi.org/10.1021/acschemneuro.8b00250.
24. Paslawski, W., M. Andreasen, …, D. E. Otzen. 2014. High stability and cooperative unfolding of α-synuclein oligomers. *Biochemistry.* 53:6252–6263. https://doi.org/10.1021/bi5007833.
25. Calabrese, M. F., and A. D. Miranker. 2007. Formation of a stable oligomer of β-2 microglobulin requires only transient encounter with Cu(II). *J. Mol. Biol.* 367:1–7. https://doi.org/10.1016/j.jmb.2006.12.034.
26. Karamanos, T. K., M. P. Jackson, …, S. E. Radford. 2019. Structural mapping of oligomeric intermediates in an amyloid assembly pathway. *Elife.* 8:1–32. https://doi.org/10.7554/eLife.46574.001.
27. Miti, T., M. Mulaj, …, M. Muschol. 2015. Stable, metastable, and kinetically trapped amyloid aggregate phases. *Biomacromolecules.* 16:326–335. https://doi.org/10.1021/bm501521r.
28. Nick, M., Y. Wu, …, W. F. DeGrado. 2018. A long-lived Aβ oligomer resistant to fibrillization. *Biopolymers.* 109:e23096. https://doi.org/10.1002/bip.23096.
29. Kuo, Y.-M., S. Webster, …, A. E. Roher. 1998. Irreversible dimerization/tetramerization and post-translational modifications inhibit proteolytic degradation of Aβ peptides of Alzheimer's disease. *Biochim. Biophys. Acta.* 1406:291–298. https://doi.org/10.1016/S0925-4439(98)00014-3.
30. Ehrnhoefer, D. E., J. Bieschke, …, E. E. Wanker. 2008. EGCG redirects amyloidogenic polypeptides into unstructured, off-pathway oligomers. *Nat. Struct. Mol. Biol.* 15:558–566. https://doi.org/10.1038/nsmb.1437.
31. Nedumpully-Govindan, P., A. Kakinen, …, F. Ding. 2016. Stabilizing off-pathway oligomers by polyphenol nanoassemblies for IAPP aggregation inhibition. *Sci. Rep.* 6:19463. https://doi.org/10.1038/srep19463.
32. Chang, X., D. Keller, …, J. J. Led. 2002. NMR studies of the aggregation of glucagon-like peptide-1: formation of a symmetric helical dimer. *FEBS Lett.* 515:165–170. https://doi.org/10.1016/S0014-5793(02)02466-3.
33. Kamgar-Parsi, K., L. Hong, …, A. Ramamoorthy. 2017. Growth-incompetent monomers of human calcitonin lead to a noncanonical direct relationship between peptide concentration and aggregation lag time. *J. Biol. Chem.* 292:14963–14976. https://doi.org/10.1074/jbc.M117.791236.
34. Deva, T., N. Lorenzen, …, D. E. Otzen. 2013. Off-pathway aggregation can inhibit fibrillation at high protein concentrations. *Biochim. Biophys. Acta.* 1834:677–687. https://doi.org/10.1016/j.bbapap.2012.12.020.
35. Souillac, P. O., V. N. Uversky, and A. L. Fink. 2003. Structural transformations of oligomeric intermediates in the fibrillation of the immunoglobulin light chain LEN. *Biochemistry.* 42:8094–8104. https://doi.org/10.1021/bi034652m.
36. Bothe, J. R., A. Andrews, …, S. Kashi. 2019. Peptide oligomerization memory effects and their impact on the physical stability of the GLP-1 agonist liraglutide. *Mol. Pharm.* 16:2153–2161. https://doi.org/10.1021/acs.molpharmaceut.9b00106.
37. Gade Malmos, K., L. M. Blancas-Mejia, …, D. Otzen. 2017. ThT 101: a primer on the use of thioflavin T to investigate amyloid formation. *Amyloid.* 24:1–16. https://doi.org/10.1080/13506129.2017.1304905.
38. Schuck, P. 2000. Size-distribution analysis of macromolecules by sedimentation velocity ultracentrifugation and Lamm equation modeling. *Biophys. J.* 78:1606–1619. https://doi.org/10.1016/S0006-3495(00)76713-0.
39. Ma, S., X. Cao, …, L. A. Nafie. 2007. Vibrational circular dichroism shows unusual sensitivity to protein fibril formation and development in solution. *J. Am. Chem. Soc.* 129:12364–12365. https://doi.org/10.1021/ja074188z.
40. Measey, T. J., K. B. Smith, …, R. Schweitzer-Stenner. 2009. Self-aggregation of a polyalanine octamer promoted by its C-terminal tyrosine and probed by a strongly enhanced vibrational circular dichroism signal. *J. Am. Chem. Soc.* 131:18218–18219. https://doi.org/10.1021/ja908324m.
41. Measey, T. J., and R. Schweitzer-Stenner. 2011. Vibrational circular dichroism as a probe of fibrillogenesis: the origin of the anomalous intensity enhancement of amyloid-like fibrils. *J. Am. Chem. Soc.* 133:1066–1076. https://doi.org/10.1021/ja1089827.
42. Keiderling, T. A. 2020. Structure of condensed phase peptides : insights from vibrational circular dichroism and Raman optical activity techniques. *Chem. Rev.* 120:3381–3419. https://doi.org/10.1021/acs.chemrev.9b00636.
43. Krupová, M., J. Kessler, and P. Bouř. 2021. Polymorphism of amyloid fibrils induced by catalytic seeding: a vibrational circular dichroism study. *ChemPhysChem.* 22:83–91. https://doi.org/10.1002/cphc.202000797.
44. Van de Vondel, E., P. Baatsen, …, C. Johannessen. 2018. Vibrational circular dichroism sheds new light on the competitive effects of crowding and β-synuclein on the fibrillation process of α-synuclein. *Biochemistry.* 57:5989–5995. https://doi.org/10.1021/acs.biochem.8b00780.
45. Kurouski, D., R. K. Dukor, …, I. K. Lednev. 2012. Normal and reversed supramolecular chirality of insulin fibrils probed by vibrational circular dichroism at the protofilament level of fibril structure. *Biophys. J.* 103:522–531. https://doi.org/10.1016/j.bpj.2012.04.042.
46. Kurouski, D., R. A. Lombardi, …, L. A. Nafie. 2010. Direct observation and pH control of reversed supramolecular chirality in insulin fibrils by vibrational circular dichroism. *Chem. Commun.* 46:7154–7156. https://doi.org/10.1039/c0cc02423f.
47. Pazderková, M., T. Pazderka, …, L. A. Nafie. 2017. Origin of enhanced VCD in amyloid fibril spectra: effect of deuteriation and pH. *Chirality.* 29:469–475. https://doi.org/10.1002/chir.22722.
48. Krupová, M., J. Kessler, and P. Bouř. 2020. Recent trends in chiroptical spectroscopy: theory and applications of vibrational circular dichroism and Raman optical activity. *Chempluschem.* 85:561–575. https://doi.org/10.1002/cplu.202000014.
49. Raynal, B., P. Lenormand, …, P. England. 2014. Quality assessment and optimization of purified protein samples: why and how? *Microb. Cell Fact.* 13:180–210. https://doi.org/10.1186/s12934-014-0180-6.
50. ROBINSON, A. B., and C. J. RUDD. 1974. Deamidation of glutaminyl and asparaginyl residues in peptides and proteins. *In* Current Topics in Cellular Regulation ACADEMIC PRESS, INC., pp. 247–295. https://doi.org/10.1016/B978-0-12-152808-9.50013-4.
51. Robinson, N. E., Z. W. Robinson, …, A. B. Robinson. 2004. Structure-dependent nonenzymatic deamidation of glutaminyl and asparaginyl pentapeptides. *J. Pept. Res.* 63:426–436. https://doi.org/10.1111/j.1399-3011.2004.00151.x.
52. Wright, H. T., and D. W. Urry. 1991. Nonenzymatic deamidation of asparaginyl and glutaminyl residues in protein. *Crit. Rev. Biochem. Mol. Biol.* 26:1–52. https://doi.org/10.3109/10409239109081719.

53. Lau, J., P. Bloch, …, T. Kruse. 2015. Discovery of the once-weekly glucagon-like peptide-1 (GLP-1) analogue semaglutide. *J. Med. Chem.* 58:7370–7380. https://doi.org/10.1021/acs.jmedchem.5b00726.

54. Lau, J. L., and M. K. Dunn. 2018. Therapeutic peptides: historical perspectives, current development trends, and future directions. *Bioorg. Med. Chem.* 26:2700–2707. https://doi.org/10.1016/j.bmc.2017.06.052.

55. Kaspar, A. A., and J. M. Reichert. 2013. Future directions for peptide therapeutics development. *Drug Discov. Today.* 18:807–817. https://doi.org/10.1016/j.drudis.2013.05.011.

56. Gilroy, C. A., K. M. Luginbuhl, and A. Chilkoti. 2016. Controlled release of biologics for the treatment of type 2 diabetes. *J. Control. Release.* 240:151–164. https://doi.org/10.1016/j.jconrel.2015.12.002.

57. D'Addio, S. M., J. R. Bothe, …, A. C. Templeton. 2016. New and evolving techniques for the characterization of peptide therapeutics. *J. Pharm. Sci.* 105:2989–3006. https://doi.org/10.1016/j.xphs.2016.06.011.

58. Becher, F.J. 2020. Studies of the physical stability of GLP-1 & chemically modified forms of GLP-1. https://doi.org/10.17863/CAM.78083.

59. Törnquist, M., T. C. T. Michaels, …, S. Linse. 2018. Secondary nucleation in amyloid formation. *Chem. Commun.* 54:8667–8684. https://doi.org/10.1039/c8cc02204f.

60. Bitan, G., M. D. Kirkitadze, …, D. B. Teplow. 2003. Amyloid -protein (A) assembly: a 40 and A 42 oligomerize through distinct pathways. *Proc. Natl. Acad. Sci. USA.* 100:330–335. https://doi.org/10.1073/pnas.222681699.

61. Dasari, A. K. R., R. M. Hughes, …, K. H. Lim. 2019. Transthyretin aggregation pathway toward the Formation of distinct cytotoxic oligomers. *Sci. Rep.* 9:10–33. https://doi.org/10.1038/s41598-018-37230-1.

62. Cremades, N., S. I. A. Cohen, …, D. Klenerman. 2012. Direct observation of the interconversion of normal and toxic forms of α-synuclein. *Cell.* 149:1048–1059. https://doi.org/10.1016/j.cell.2012.03.037.

63. Fusco, G., S. W. Chen, …, A. De Simone. 2017. Structural basis of membrane disruption and cellular toxicity by α-synuclein oligomers. *Science.* 358:1440–1443. https://doi.org/10.1126/science.aan6160.

64. Wu, J. W., L. Breydo, …, C. Glabe. 2010. Fibrillar oligomers nucleate the oligomerization of monomeric amyloid β but do not seed fibril formation. *J. Biol. Chem.* 285:6071–6079. https://doi.org/10.1074/jbc.M109.069542.

65. Bolognesi, B., J. R. Kumita, …, J. J. Yerbury. 2010. ANS binding reveals common features of cytotoxic amyloid species. *ACS Chem. Biol.* 5:735–740. https://doi.org/10.1021/cb1001203.

66. Hnath, B., and N. V. Dokholyan. 2022. Toxic SOD1 trimers are off-pathway in the formation of amyloid-like fibrils in ALS. *Biophys. J.* 121:2084–2095. https://doi.org/10.1016/j.bpj.2022.04.037.

67. Baskakov, I. V., G. Legname, …, F. E. Cohen. 2002. Pathway complexity of prion protein assembly into amyloid. *J. Biol. Chem.* 277:21140–21148. https://doi.org/10.1074/jbc.M111402200.

68. Sanders, A., C. Jeremy Craven, …, R. A. Staniforth. 2004. Cystatin forms a tetramer through structural rearrangement of domain-swapped dimers prior to amyloidogenesis. *J. Mol. Biol.* 336:165–178. https://doi.org/10.1016/j.jmb.2003.12.011.

69. Staniforth, R. A., S. Giannini, …, J. P. Waltho. 2001. Three-dimensional domain swapping in the folded and molten-globule states of cystatins, an amyloid-forming structural superfamily. *EMBO J.* 20:4774–4781. https://doi.org/10.1093/emboj/20.17.4774.

70. Turner, A. L., M. Watson, …, K. Stott. 2018. Highly disordered histone H1−DNA model complexes and their condensates. *Proc. Natl. Acad. Sci. USA.* 115:11964–11969. https://doi.org/10.1073/pnas.1805943115.

71. Borgia, A., M. B. Borgia, …, B. Schuler. 2018. Extreme disorder in an ultrahigh-affinity protein complex. *Nature.* 555:61–66. https://doi.org/10.1038/nature25762.

72. Maya-Martinez, R., Y. Xu, …, S. E. Radford. 2022. Dimers of D76N-β2-microglobulin display potent antiamyloid aggregation activity. *J. Biol. Chem.* 298:102659. https://doi.org/10.1016/j.jbc.2022.102659.

73. Soreghan, B., J. Kosmoski, and C. Glabe. 1994. Surfactant properties of Alzheimer's A beta peptides and the mechanism of amyloid aggregation. *J. Biol. Chem.* 269:28551–28554. https://doi.org/10.1016/S0021-9258(19)61939-3.

74. Hasecke, F., T. Miti, …, M. Muschol. 2018. Origin of metastable oligomers and their effects on amyloid fibril self-assembly. *Chem. Sci.* 9:5937–5948. https://doi.org/10.1039/C8SC01479E.

75. Hasecke, F., C. Niyangoda, …, W. Hoyer. 2021. Protofibril–fibril interactions inhibit amyloid fibril assembly by obstructing secondary nucleation. *Angew. Chemie.* 133:3053–3058. https://doi.org/10.1002/ange.202010098.

Biophysical Journal, Volume *122*

Supplemental information

Glucagon-like peptide 1 aggregates into low-molecular-weight oligomers off-pathway to fibrillation

Eva Přáda Brichtová, Monika Krupová, Petr Bouř, Viv Lindo, Ana Gomes dos Santos, and Sophie E. Jackson

Supplemental information

Glucagon-like peptide 1 aggregates into low molecular weight oligomers off-pathway to fibrillation

Eva Přáda Brichtová,[1] Monika Krupová,[2,3] Petr Bouř,[2] Viv Lindo,[4] Ana Gomes dos Santos[4] & Sophie E. Jackson[1,*]

[1]Yusuf Hamied Department of Chemistry, University of Cambridge, Cambridge, CB2 1EW, United Kingdom

[2]Institute of Organic Chemistry and Biochemistry, Academy of Sciences, Flemingovo náměstí 2, Prague 6, 166 10, Czech Republic

[3]Hylleraas Centre for Quantum Molecular Sciences, Department of Chemistry, UiT The Arctic University of Norway, Tromsø, N-9037, Norway

[4]AstraZeneca, Aaron Klug Building, Granta Park, Cambridge, CB21 6GH, United Kingdom

*Correspondence: Sophie E. Jackson. sej13@cam.ac.uk

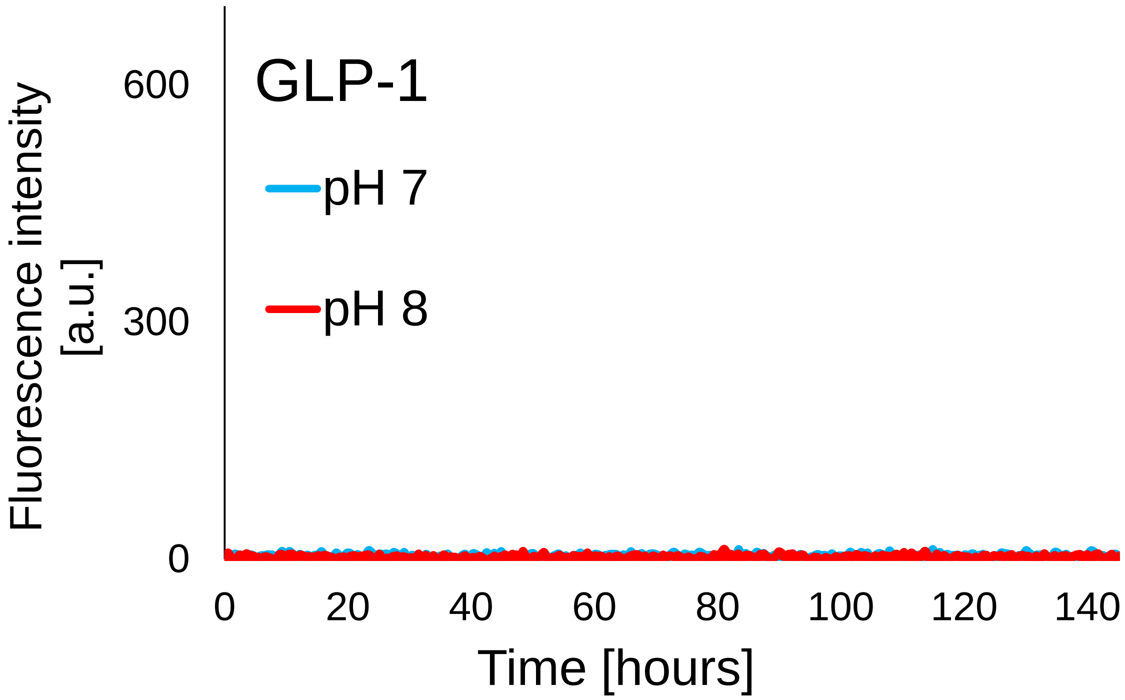

Fig. S1: Fibrillation of GLP-1 at pH 7 and 8 monitored by a ThT assay. GLP-1 at 43 µM concentration was incubated at pH 7 (10 mM sodium phosphate, shown in blue) and pH 8 (10 mM sodium phosphate, shown in red) at 37 °C with agitation over 6 days.

Size exclusion chromatography analysis of low-molecular weight oligomers of GLP-1 and GLP-1-Am

GLP-1 at pH 7, Figure 2A			
Time [days]	Content [%]		
	monomer	oligomers	other
0	99	1	0
3	85	1	14
5	82	3	15
7	59	7	34
10	2	41	57

GLP-1 at pH 8, Figure 2B			
Time [days]	Content [%]		
	monomer	oligomers	other
0	98	2	0
5	81	15	4

GLP-1-Am at pH 8, Figure 2C			
Time [days]	Content [%]		
	monomer	oligomers	other
0	100	0	0
2	61	2	37
3	59	5	36
6	56	14	30
12	39	25	36
16	0	38	62

GLP-1-Am at pH 7, Figure 2D			
Time [days]	Content [%]		
	monomer	oligomers	other
0	100	0	0
3	20	2	78

Table S1: A quantitative estimate of relative amounts of GLP-1 and GLP-1-Am monomer and oligomers present at different time points determined using SEC. Values in this Table are obtained from the analysis of chromatograms shown in Fig. 2. A peak integration was performed in the Unicorn software of the ÄKTA instrument.

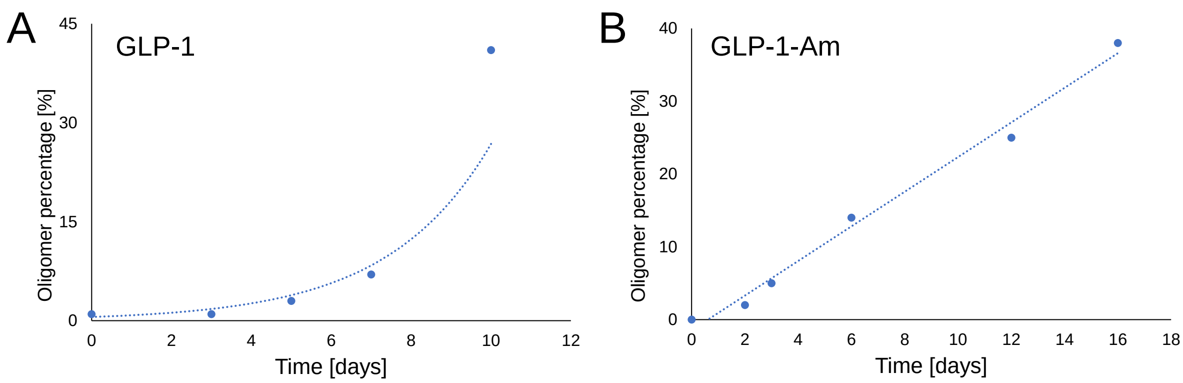

Fig. S2: An increase of relative amounts of GLP-1 and GLP-1-Am oligomers as monitored by SEC. The plots are based on the relative percentage of GLP-1 and GLP-1-Am oligomers at different time points given in **Table S1**. GLP-1 at 85 µM peptide concentration was incubated at 37 °C with agitation in 10 mM phosphate at pH 7 over 10 days (**A**). GLP-1-Am at 85 µM concentration was incubated at 37 °C with agitation in 25 mM phosphate at pH 7 over 16 days (**B**). The blue dotted line is the exponential (**A**) and linear regression (**B**) fit for the data points.

GLP-1 and GLP-1-Am low-molecular weight oligomer formation in different buffers monitored using SEC: ionic strength dependence

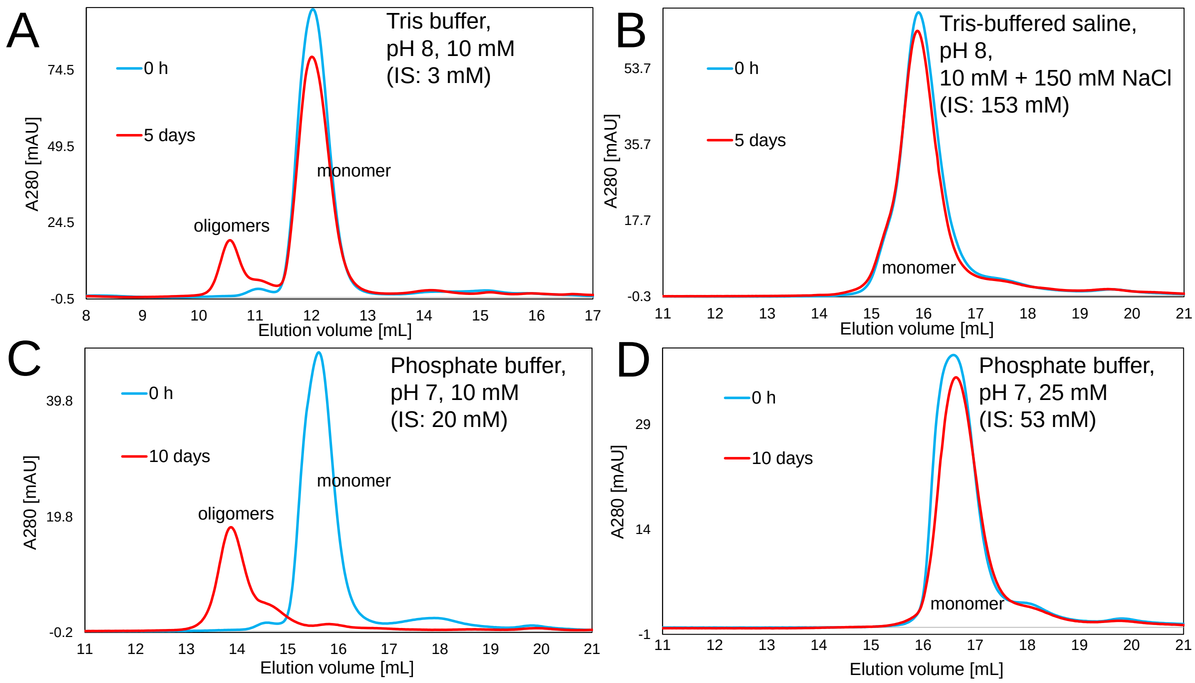

Fig. S3: Formation of low-molecular weight oligomers of GLP-1 in low and high ionic strength (IS) buffers monitored using SEC. (**A**) 150 µM GLP-1 in 10 mM Tris buffer at pH 8 (3 mM ionic strength) at time zero and after 5-day incubation (at 37 °C with shaking) were analysed using a Superdex 75 10/300 size exclusion column. (**B**) 150 µM GLP-1 in Tris-buffered saline (10 mM Tris +150 mM NaCl) at pH 8 (153 mM ionic strength) at time zero and after 5-day incubation (at 37 °C with shaking) were analysed using a Superdex 75 10/300 size exclusion column. (**C**) 85 µM GLP-1 in 10 mM sodium phosphate buffer at pH 7 (20 mM ionic strength) at time zero and after 10-day incubation (at 37 °C with shaking) were analysed using a Superose 12 10/300 size exclusion column. (**D**) 85 µM GLP-1 in 25 mM sodium phosphate buffer at pH 7 (53 mM ionic strength) at time zero and after 10-day incubation (at 37 °C with shaking) were analysed using a Superose 12 10/300 size exclusion column.

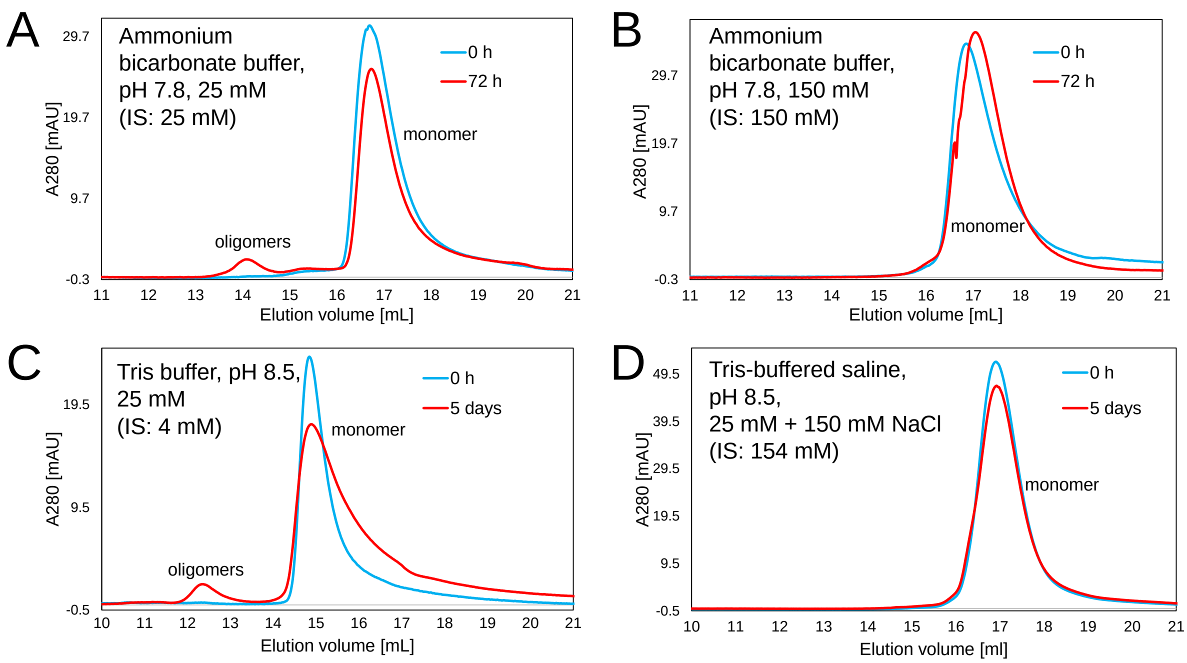

Fig. S4: Formation of low-molecular weight oligomers of GLP-1-Am in low and high ionic strength (IS) buffers. (**A**) 100 µM GLP-1-Am in 25 mM ammonium bicarbonate at pH 7.8 (ionic strength 25 mM) was analysed at time zero and after 72-hour incubation at 37 °C with agitation. (**B**) 100 µM GLP-1-Am in 150 mM ammonium bicarbonate at pH 7.8 (ionic strength 150 mM) was analysed at time zero and after 72-hour incubation at 37 °C with agitation. (**C**) 120 µM GLP-1-Am in 25 mM Tris buffer at pH 8.5 (ionic strength 4 mM) was analysed at time zero and after 5-day incubation at 37 °C with agitation. (**D**) 120 µM GLP-1-Am in Tris-buffered saline at pH 8.5 (ionic strength 154 mM, 25 mM Tris and 150 mM NaCl) was analysed at time zero and after 5-day incubation at 37 °C with agitation. All samples were analysed using a Superdex 75 10/300 size exclusion column.

Batch-to-batch reproducibility of GLP-1-Am oligomerization

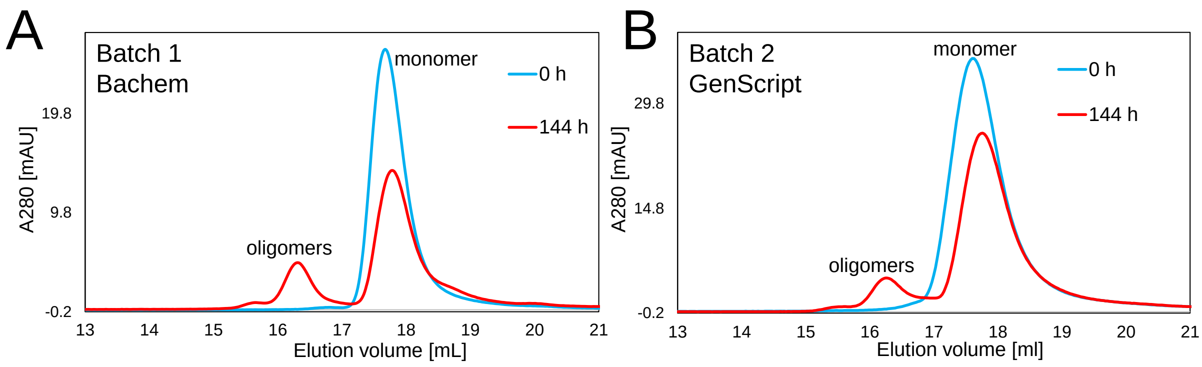

Fig. S5: Batch-to-batch reproducibility of GLP-1-Am oligomerization. 85 µM GLP-1-Am from Bachem (**A**) or GenScript (**B**) was incubated in 25 mM sodium phosphate buffer, pH 8 at 37 °C with agitation. Size-exclusion chromatograms of samples of both peptide batches were analysed on a Superose 12 10/300 column at time zero and after 144 h of incubation.

Estimation of the size of low-molecular weight oligomers of GLP-1 and GLP-1-Am using size-exclusion chromatography

Calibration of Superose 12 10/300 in 25 mM sodium phosphate buffer at pH 8.

Protein standard	MW [Da]	Elution volume [mL]
Aldolase	158,000	11.4
Conalbumin	75,000	12.2
Ovalbumin	44,000	12.6
carbonic anhydrase	29,000	13.9
Trypsinogen	24,000	14.5
ribonuclease A	13,700	15.1
chymotrypsin inhibitor 2	9,265	15.9
GLP-1-Am monomer	3,355	17.7

Table S2: Protein calibration standards and their elution volumes on a Superose 12 10/300 in 25 mM sodium phosphate buffer at pH 8.

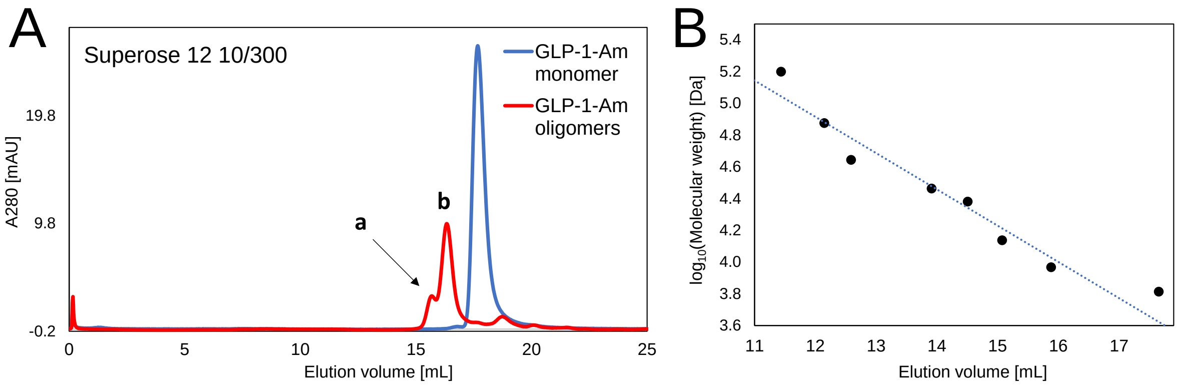

Fig. S6: Elution profiles of the GLP-1-Am monomer and oligomers on a Superose 12 10/300 in 25 mM sodium phosphate buffer at pH 8 and column calibration curve. The GLP-1-Am sample was analysed prior to incubation/shaking to induce aggregation (0 h, blue line) and after 16 days (red line) of incubation at 37 °C with continuous shaking at 180 rpm. Elution conditions: 25 mM sodium phosphate buffer at pH 8, flow rate 0.75 mL min^{-1} (**A**). Calibration performed in 25 mM sodium phosphate buffer at pH 8 on a set of protein standards (Table S2) at room temperature. Elution was performed with a flow rate 0.75 mL min^{-1} and 100 µL of approximately 100 µM of each protein was injected using a 200 µL injection loop. The protein standards elution volumes were plotted against the logarithm of their molecular weight and the correlation is described by a linear regression fit (blue dotted line) and its square of the correlation coefficient, where V_e is the protein elution volume measured from the centre of a peak (**B**), see below.

$$log_{10}(\mathrm{MW/Da}) = -0.2285 \cdot V_e + 7.6579$$

$$R^2 = 0.9565$$

The elution volumes of the GLP-1-Am oligomers (**Fig. S6**) were 15.5 mL for oligomer **a** and 16.2 mL for oligomer **b**. Using the equation above, the size of oligomer **a** was calculated as 13 kDa and 9 kDa for oligomer **b** at pH 8.

Calibration of Superdex 75 10/300 in 25 mM sodium phosphate buffer at pH 8.

Protein standard	MW [Da]	Elution volume [mL]
ovalbumin	44,000	9.8
carbonic anhydrase	29,000	11.4
trypsinogen	24,000	12.1
ribonuclease A	13,700	13.3
chymotrypsin inhibitor 2	9,265	14.4
GLP-1-Am monomer	3,355	17.1

Table S3: Protein standards and their elution volumes on a Superdex 75 10/300 in 25 mM sodium phosphate buffer at pH 8.

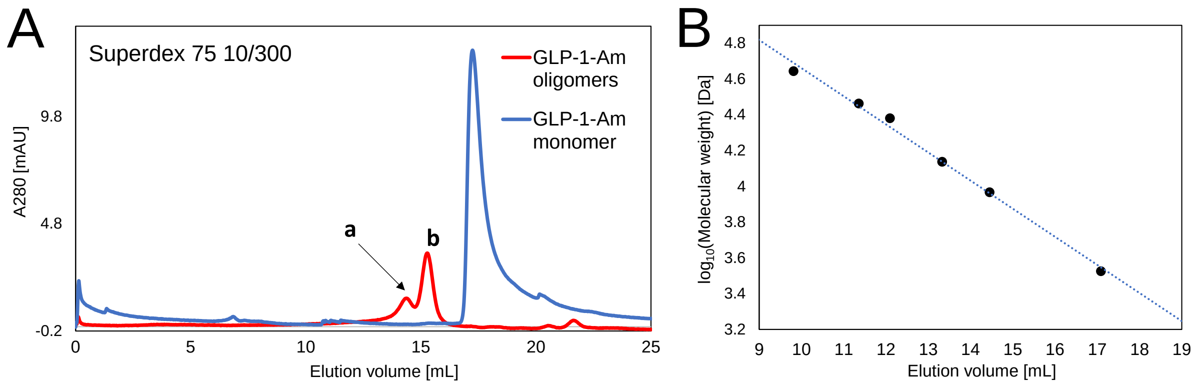

Fig. S7: Elution profiles of GLP-1-Am monomer and oligomers on a Superdex 75 10/300 in 25 mM sodium phosphate buffer at pH 8 and column calibration curve. GLP-1-Am samples were analysed prior to the incubation/shaking (0 h, blue line) and after 7 days (red line) of incubation at 37 °C with continuous shaking at 180 rpm. Elution conditions: 25 mM sodium phosphate buffer at pH 8, flow rate 0.75 mL min^{-1} (**A**). Calibration performed in 25 mM sodium phosphate buffer at pH 8 on a set of protein standards (Table S3) at room temperature. Elution was performed at a flow rate of 0.75 mL·min^{-1} and 100 µL of approximately 100 µM of each protein was injected using a 200 µL injection loop. The blue dotted line is the linear regression fit (**B**), see below.

$$log_{10}(\mathrm{MW/Da}) = -0.1571 \cdot V_e + 6.232$$

$$R^2 = 0.9935$$

The elution volumes of the GLP-1-Am oligomers (**Fig. S7**) were 14.4 mL for oligomer **a** and 15.3 mL for oligomer **b**. Using the equation above, the size of oligomer **a** was calculated as 9.3 kDa and 6.7 kDa for oligomer **b**.

Results of both SEC columns indicate that eluted species are low molecular weight oligomers of a size range from 6.7 kDa to 13 kDa at pH 8. These values correspond to a range of species from peptide dimers (6710 Da) to peptide tetramers (13 420 Da).

Calibration of Superose 12 10/300 in 10 mM sodium phosphate buffer at pH 7.

Protein standard	MW [Da]	Elution volume [mL]
Aldolase	158,000	11.4
Conalbumin	75,000	11.7
Ovalbumin	44,000	10.7
carbonic anhydrase	29,000	13.1
chymotrypsin inhibitor 2	9,265	15.8
GLP-1 monomer	3,355	15.6

Table S4: Protein standards and their elution volumes on a Superose 12 10/300 in 10 mM sodium phosphate buffer at pH 7.

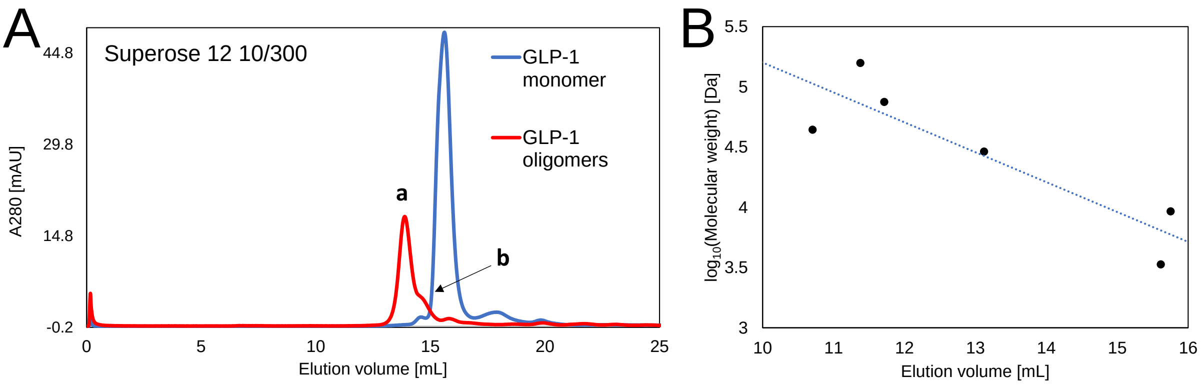

Fig. S8: Elution profiles of GLP-1 monomer and oligomers on a Superose 12 10/300 in 10 mM sodium phosphate buffer at pH 7 and column calibration curve. GLP-1 samples were analysed prior to the incubation/shaking (0 h, blue line) and after 10 days (red line) of incubation at 37 °C with continuous shaking at 180 rpm. Elution conditions: 10 mM sodium phosphate buffer at pH 7, flow rate 0.75 mL min^{-1} (**A**). Calibration performed in 10 mM sodium phosphate buffer at pH 7 on a set of protein standards (Table S4) at room temperature. Elution was performed at a flow rate of 0.75 mL·min^{-1} and 100 µL of approximately 100 µM of each protein was injected using a 200 µL injection loop. The blue dotted line is the linear regression fit (**B**), see below.

$$log_{10}(\mathrm{MW/Da}) = -0.2482 \cdot V_{\mathrm{e}} + 7.684$$

$$R^2 = 0.7909$$

The elution volumes of the GLP-1-Am oligomers (**Fig. S8**) were 13.9 mL for oligomer **a** and 14.6 mL for oligomer **b**. Using the equation above, the size of oligomer **a** was calculated as 17.1 kDa and 11.5 kDa for oligomer **b** at pH 7.

Sedimentation velocity analysis of low-molecular weight oligomers of GLP-1 and GLP-1-Am

GLP-1 fresh samples				GLP-1-Am fresh samples		
$s_{20,w}$ [S]	MW [kDa]	Content [%]		$s_{20,w}$ [S]	MW [kDa]	Content [%]
0.15	N/A* (monomer)	42		0.15	N/A* (monomer)	46
0.75	7.39 (dimer)	45		0.91	6.09 (dimer)	46
1.67	23.9 (7-mer)	10		1.97	18.0 (5-mer)	8
**GLP-1 7-day incubation**				**GLP-1-Am 7-day incubation**		
$s_{20,w}$ [S]	MW [kDa]	Content [%]		$s_{20,w}$ [S]	MW [kDa]	Content [%]
0.15	N/A* (monomer)	20		0.15	N/A* (monomer)	48
0.60	4.73 (dimer)	58		0.77	7.49 (dimer)	34
1.52	16.7 (5-mer)	16		1.24	14.5 (4-mer)	13
2.73	43.6 (13-mer)	6		2.01	27.7 (8-mer)	4

Table S5: Quantitative estimation of mass and relative content of species detected in sedimentation velocity experiments. The data correspond to the sedimentation profiles shown in Fig. 3. The molecular weight and relative content of detected species was calculated in a Sedfit program. * It was not possible to calculate the mass of GLP-1/GLP-1-Am monomer in a Sedfit program due to its limited sedimentation. The molecular weight of the GLP-1/GLP-1-Am monomer is approximately 3.35 kDa.

Secondary structure content of monomeric GLP-1-Am, low-molecular weight oligomers and fibrils of GLP-1-Am determined from far-UV CD spectra

GLP-1-Am	α-helix (%)	β-sheet (%)	turns (%)	disordered (%)
monomer	31	15	23	31
oligomers	25	6	25	44
fibrils	5	39	15	41

Table S6: Secondary structure content of monomeric GLP-1-Am, low molecular weight oligomers and fibrils of GLP-1-Am in 25 mM sodium phosphate buffer at pH 8 estimated from far UV CD spectra. Far-UV CD spectra of GLP-1-Am monomer, low-molecular weight oligomers, and fibrils shown in Figure 4A were analysed using DichroWeb, Contin-LL method, dataset 3 (1–3).

Fibrillation of GLP-1-Am at pH/pD 3 characterised by FT-IR, VCD and TEM

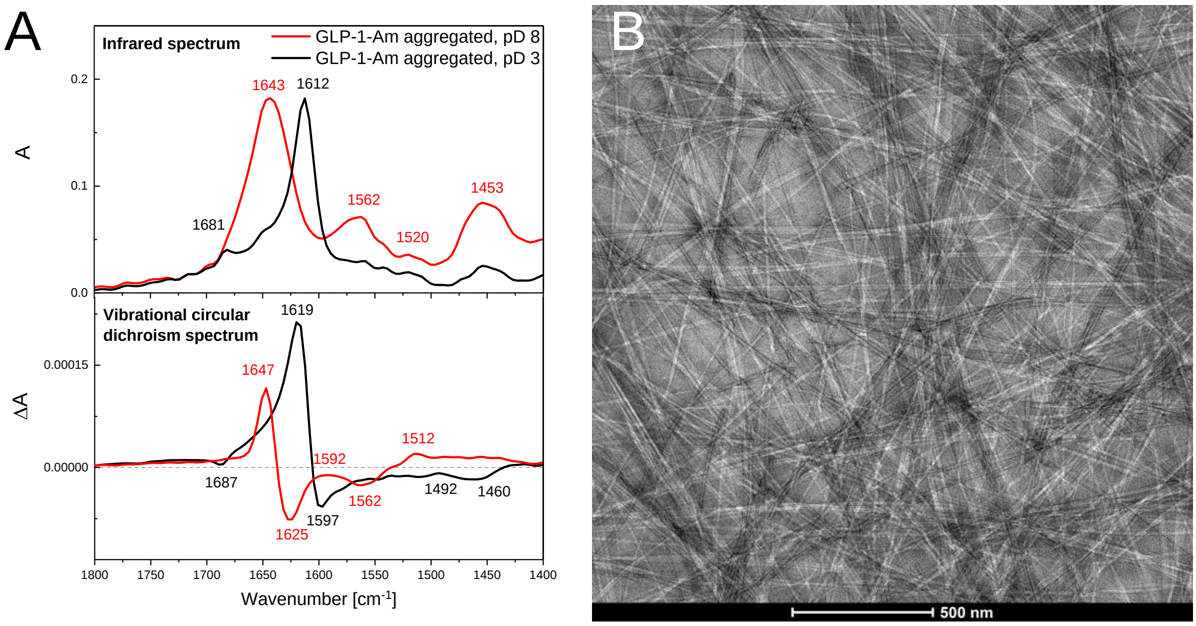

Fig. S9: Aggregation of GLP-1-Am at pD/pH 3. (**A**) FT-IR and VCD spectra of aggregated GLP-1-Am at pD 3 and pD 8. Samples of 1.2 mM GLP-1-Am in 25 mM deuterated sodium phosphate buffer at pD 3 and pD 8 were incubated for 8 days at 37 °C with 180 rpm agitation. The FT-IR and VCD spectra have been normalized to the amide I absorption. (**B**) GLP-1 samples at 85 µM concentration were incubated in 25 mM citrate at pH 3 at 37 °C with agitation for 6 days prior to the imaging. Aged samples were applied onto a carbon coated copper grid and negatively stained using 2 % uranyl acetate.

Characterization of low-molecular weight oligomers of GLP-1 using far-UV CD and intrinsic Trp fluorescence

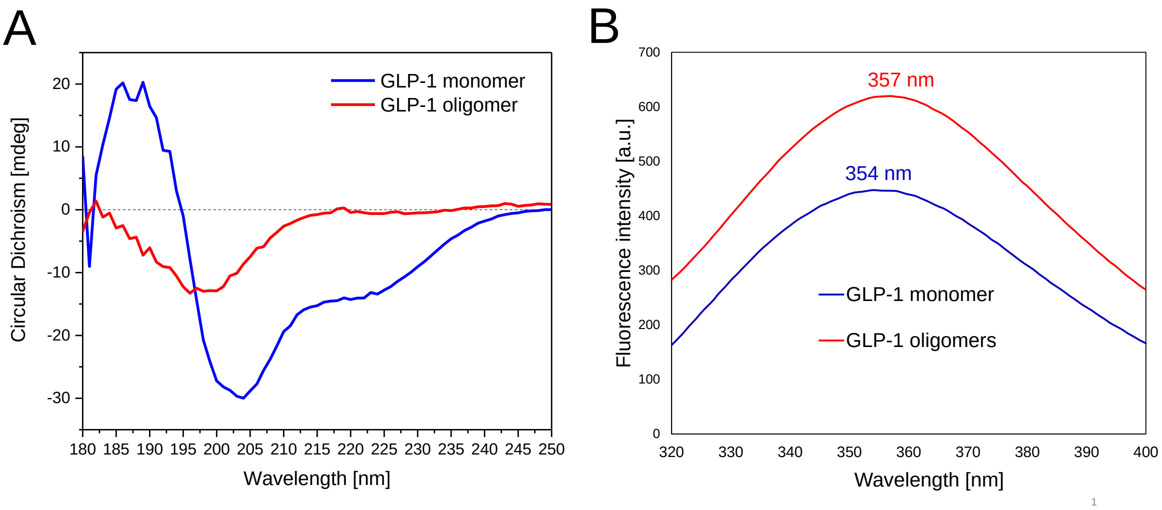

Fig. S10: Far-UV CD spectra (A) and intrinsic tryptophan fluorescence emission spectra (B) of monomeric GLP-1 and low-molecular weight oligomers of GLP-1. Spectra of the monomeric GLP-1 were measured immediately after peptide dissolution in 10 mM sodium phosphate buffer at pH 7. Spectra of GLP-1 oligomers were measured after 10-day incubation at 37 °C with agitation when all monomer was depleted as shown by SEC.

SEC elution profiles and absorption spectra of GLP-1 monomer and low molecular-weight oligomers

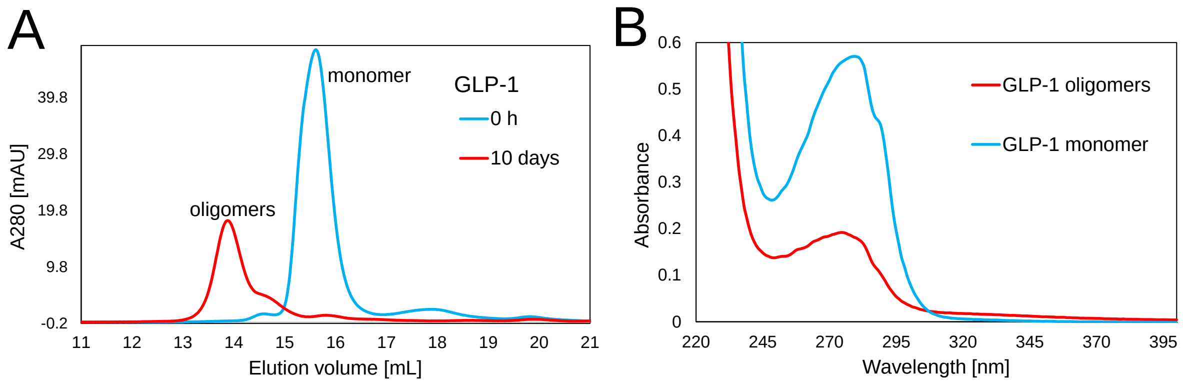

Fig. S11: SEC elution volumes and absorption spectra of GLP-1 monomer and low molecular weight oligomers. (**A**) GLP-1 was incubated in 10 mM sodium phosphate buffer at pH 7, at 37 °C with agitation. This sample was analysed using SEC (Superose 12 10/300) at different time points. Figure A shows the elution profile corresponding to the monomer of a freshly prepared GLP-1 sample along with the elution profile of oligomers of GLP-1 which were formed after 10 days of sample incubation. (**B**) UV absorption spectra of monomer and oligomer as described in (**A**). The oligomeric GLP-1 shows detectable light scattering as apparent from an increased absorption at around 320 nm. The concentration of oligomers is lower than the initial concentration of the peptide due to the competing aggregation/fibrillation processes that resulted in the formation of large insoluble aggregates which were removed by filtration prior to the analysis. Considering the small size of these oligomers, they show a surprisingly high level of light scattering as observed in the UV-Vis absorption spectra, which may suggest their further coagulation in the solution.

Calcein-release assay with low-molecular weight oligomers of GLP-1-Am

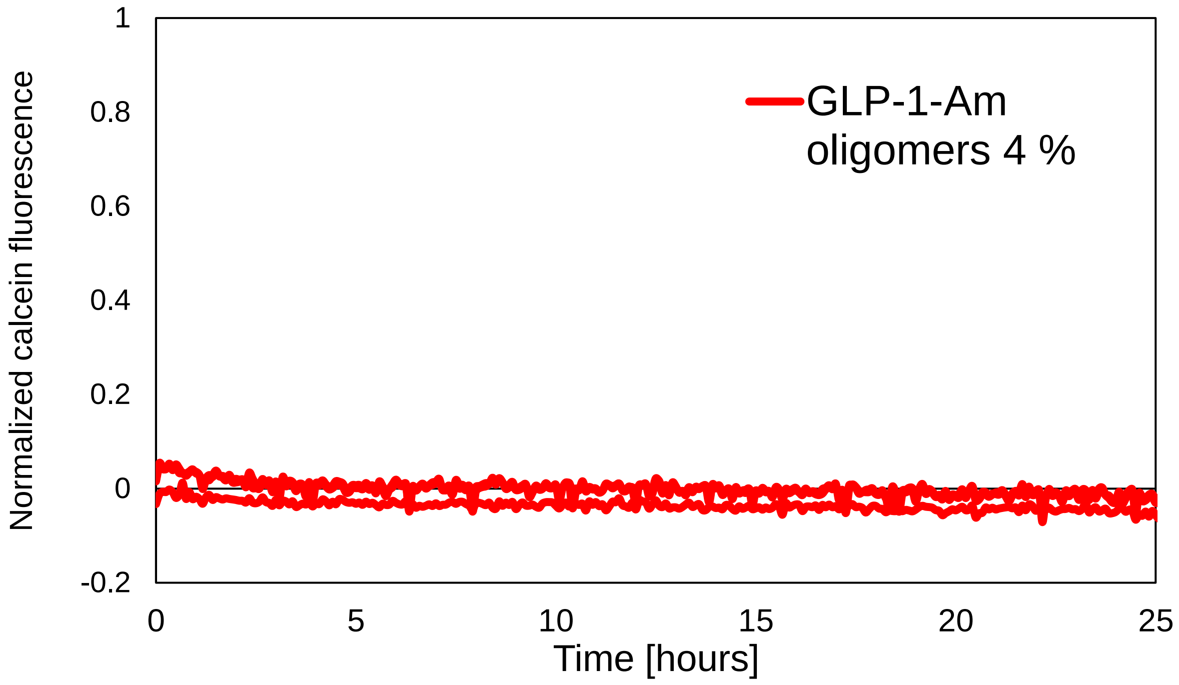

Fig. S12: Calcein-release assay with 4 % of low-molecular weight oligomers of GLP-1-Am. The assay was performed with 50 µM 1,2-dioleoyl-sn-glycero-3-phospho-L-serine (DOPS) vesicles at pH 8 in 37 °C over 25 hours.

Calcein-release assay. The assay was performed with 50 µM of calcein-encapsulated 1,2-dioleoyl-sn-glycero-3-phospho-L-serine (DOPS) vesicles of an average diameter of 125 nm (calcein concentration inside vesicles >70 mM). The assay buffer was 20 mM Hepes, 120 mM NaCl, 0.8 mM EDTA, pH 8.0. Low-molecular weight oligomers of GLP-1-Am were isolated using SEC from aged sample of 200 µM peptide incubated in 25 mM sodium phosphate at pH 8 for 7 days. Isolated oligomers were concentrated using Amicon Ultra Centrifugal Filter Unit with 3 kDa MWCO. The fluorescence emission signal at 520 nm was recorded every 5 minutes upon 484 nm excitation. Samples were incubated at 37 °C with agitation during the assay. The sample was measured in a triplicate. Fluorescence kinetic measurements were carried out using a microplate reader FLUOstar Omega (BMG Labtech). Samples calcein-encapsulated vesicle with 4 % (w/w) of GLP-1-Am oligomers were pipetted into a 96-well half-area plate (Corning 3694) and sealed with transparent tape to prevent samples from evaporation. The total volume of each sample in a well was 100 µL. Fluorescence was measured at a gain of 800 with 8 flashes per well. At the end of the assay 5 % (v/v) Triton X100 to each well which causes instant disruption of vesicles. The fluorescence signal was then normalized, taking the fluorescence value after the addition of 5 % (v/v) Triton X100 (complete disruption of vesicles) as 1.

Assessment of GLP-1-Am oligomer stability using SEC

Re-injection stability

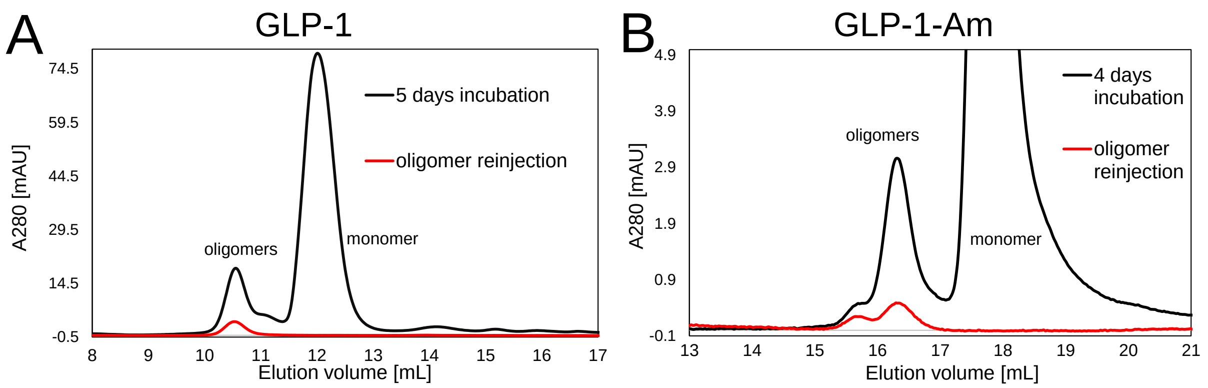

Fig. S13: Stability of low-molecular weight oligomers of GLP-1 and GLP-1-Am upon reinjection onto a size-exclusion column. (**A**) 150 µM GLP-1 was analysed after 5-day incubation at 37 °C with agitation in 10 mM Tris buffer at pH 8, oligomeric fractions were isolated and immediately reinjected onto the Superdex 75 10/300 size-exclusion column. (**B**) 85 µM GLP-1-Am was analysed after 4-day incubation at 37 °C with agitation in 25 mM sodium phosphate buffer at pH 8, oligomeric fractions were isolated and immediately reinjected onto the Superose 12 10/300 size-exclusion column.

Stability with respect to sonication, 20 % isopropanol, 40 % acetonitrile, or heating

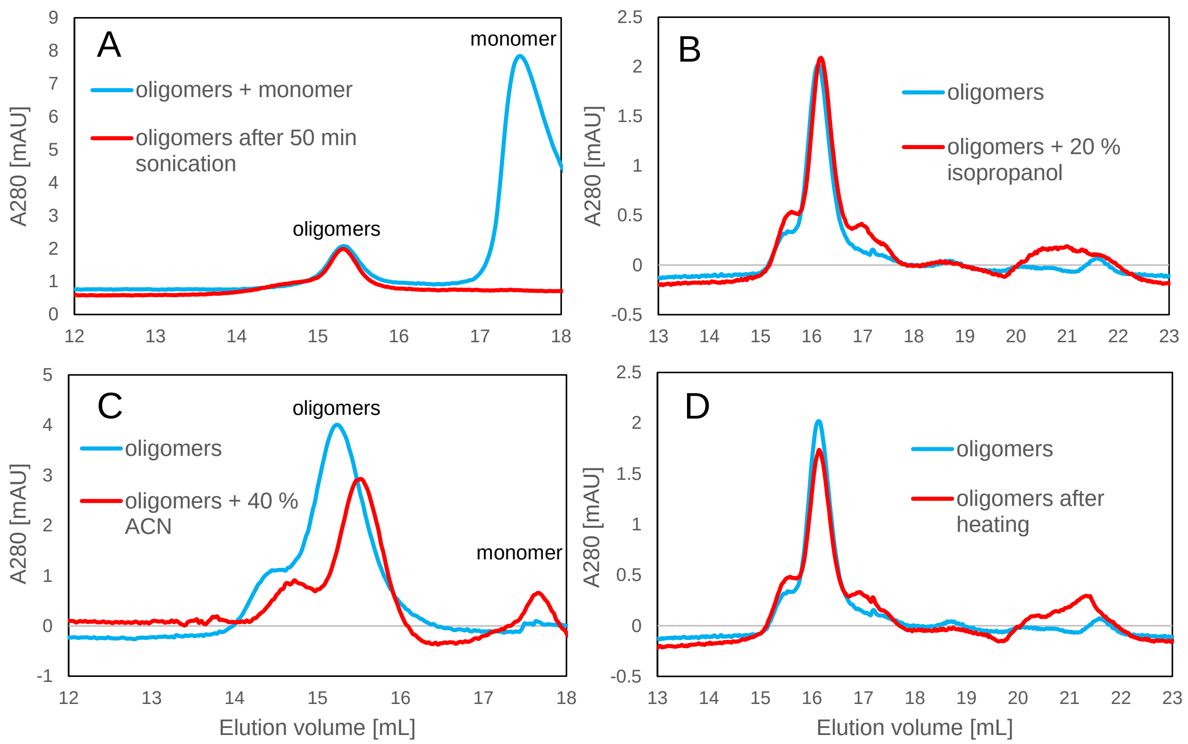

Fig. S14: Assessment of GLP-1-Am oligomer stability using SEC. SEC elution curves before (blue line) and after (red line) a stability test. Samples A and C were analysed on a Superdex 75 10/300 column, and B and D on a Superose 12 10/300 SEC column. After incubation (in 25 mM sodium phosphate buffer at pH 8 with continuous shaking at 180 rpm, for 3–9 days), oligomeric peaks were first separated by SEC (for B, C, D only) and, after the particular stability test, were reinjected onto a SEC column in 25 mM sodium phosphate buffer at pH 8. The samples were: A mixture of the monomer and oligomers sonicated for 50 min (**A**). Isolated oligomers incubated in 20% isopropanol for 1 hour (**B**), incubated in 40% acetonitrile (ACN) for 2 hours (**C**), or heated to 95 °C for 10 min (**D**).

Liquid chromatography-mass spectrometry (LC-MS) analysis of GLP-1-Am oligomers

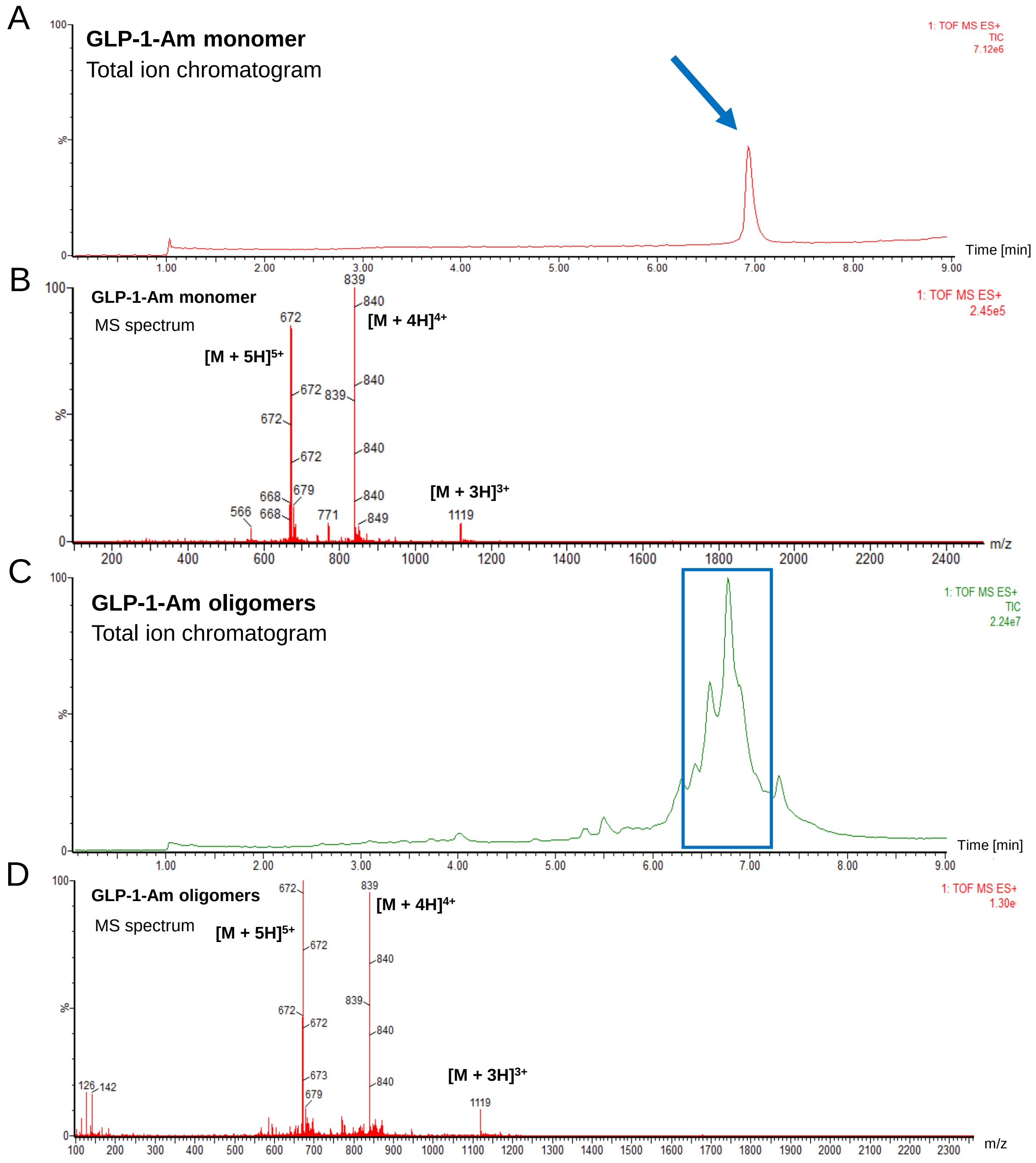

Fig. S15: LC-MS analysis of monomeric and oligomeric GLP-1-Am. Total ion chromatogram of monomeric GLP-1-Am – the blue arrow indicates the peptide peak on the total ion chromatogram (**A**), corresponding mass spectrum is shown in (**B**). LC-MS analysis of GLP-1-Am oligomers (**C, D**). The total ion chromatogram shows multiple overlapping peaks (**C**). All the peaks in the area marked with the blue rectangle correspond to the MS spectrum in (**D**) which is consistent with the spectrum of the GLP-1-Am monomer.

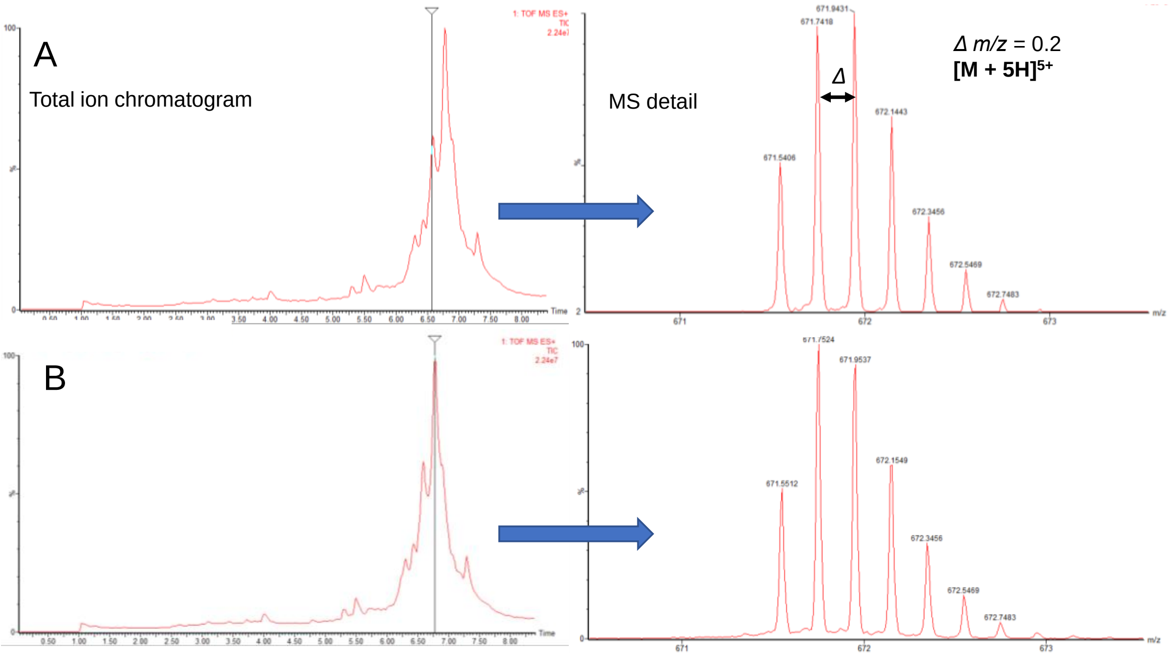

Fig. S16: LC-MS analysis of GLP-1-Am oligomers – isotopic distribution. The isotopic distribution of the peak with *m/z* around 672 was analysed at two different time points in the total ion chromatogram (**A** and **B**). Both isotopic distributions correspond exactly to the five times charged state of the GLP-1-Am monomer.

LC-MS: Liquid chromatography gradient and elution conditions

**LC method**		
**Flow rate**	0.2 mL min^{-1}	
**Run time**	11.55 min	
**Sample injection**	6 µL	
**Solvent system**		
**A**	H_2O + 0.1% formic acid	
**B**	75% acetonitrile, 25% H_2O, 0.075% formic acid	
**Gradient**		
**Time [min]**	**A (%)**	**B (%)**
0.00	95	5
2.00	80	20
9.00	50	50
9.21	0	100
11.55	95	5

Table S7: Waters' Xevo G2-S LC method.

Size exclusion chromatography and sedimentation velocity of freshly prepared samples of GLP-1-Am and βAsp_GLP-1-Am

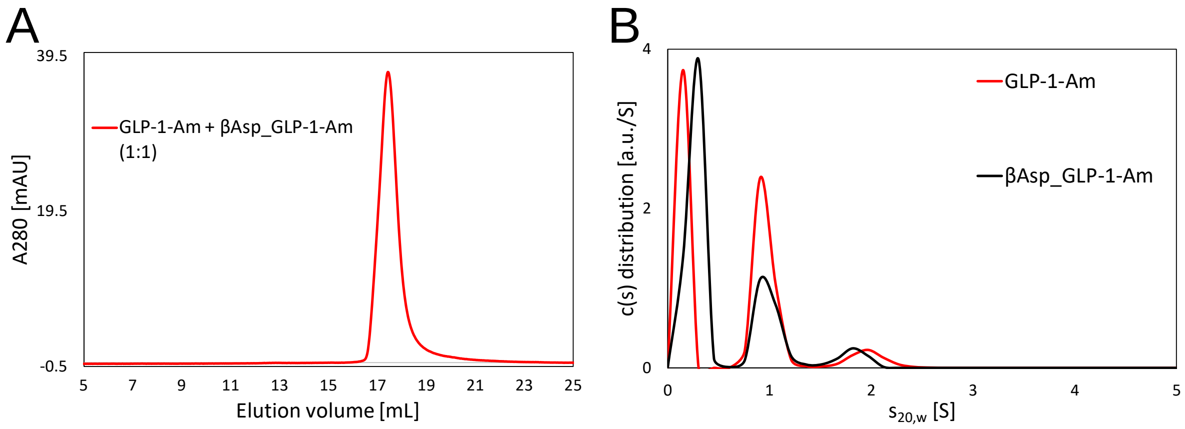

Fig. S17: Size-exclusion chromatography and sedimentation velocity of freshly prepared GLP-1-Am and βAsp_GLP-1-Am. (A) Size-exclusion chromatography of a freshly prepared mixed sample of GLP-1-Am and βAsp_GLP-1-Am in a 1:1 ratio at a total concentration of 85 µM in 25 mM phosphate, pH 8. Size-exclusion chromatography was performed using a Superose12 10/300 column. **(B)** Sedimentation velocity of freshly prepared samples of GLP-1-Am and βAsp_GLP-1-Am at 85 µM in 25 mM phosphate pH 8. After 2-hour temperature equilibration to 20 °C, the experiment was performed at 50 000 rpm using a Beckman Optima XL-I Analytical Ultracentrifuge. The interference sedimentation curves were collected as 300 scans (approx. 24 h run) and fitted to a continuous c(*s*) distribution model implemented in a Sedfit program (4). The sedimentation coefficient was corrected for the standard state of water at 20 °C ($s_{20,w}$).

Isoelectric focusing gel electrophoresis of aged and fresh sample of GLP-1-Am, βAsp_GLP-1-Am and GLP-1

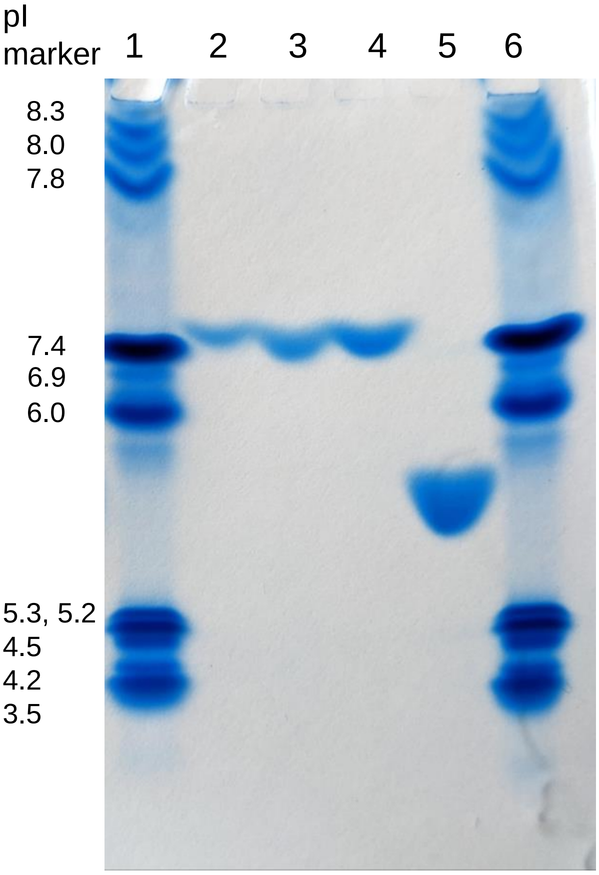

Fig. S18: Isoelectric focusing gel electrophoresis of fresh and aged GLP-1-Am, βAsp_GLP-1-Am and GLP-1. Lanes 1 and 6: pI protein standard markers, lane 2: GLP-1-Am incubated at 85 µM peptide concentration in 25 mM phosphate, pH 8, at 37 °C with agitation for 8 days, lane 3: GLP-1-Am (fresh sample), lane 4: βAsp_GLP-1-Am (fresh sample), lane 5: GLP-1 (fresh sample). Values of the isoelectric point (pI) standards are given on the left side of the gel image. pI value of samples in lanes 2, 3, 4 is at around 7.4; pI of a sample in lane 5 is somewhere in a range of 5.5–5.8 as determined from the gel.

Isoelectric focusing electrophoresis. The isoelectric point (pI) of aged and fresh samples of GLP-1-Am, βAsp_GLP-1-Am and GLP-1 was determined by isoelectric focusing (IEF) gel Novex™ pH 3-10 run in buffers from Novex™ pH 3–10 IEF Buffer Kit at a constant voltage of 200 V for 1 hour. Peptide samples were prepared in a sample buffer (Novex™ pH 3-10 IEF Buffer Kit) and immediately loaded onto the gel. A protein marker for isoelectric focusing SERVA Liquid mix 3–10 was run in parallel for pI reference. Gels were stained with InstantBlue (Expedeon).

Effect of low-molecular weight oligomers on fibrillation kinetics:

Thioflavin T assay of GLP-1-Am with the addition of pre-formed oligomers

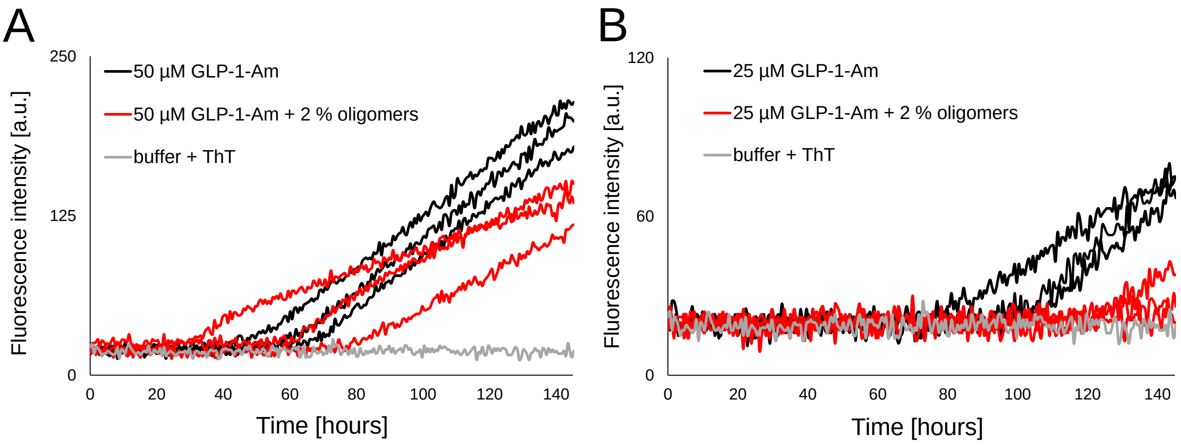

Fig. S19: Thioflavin T assay of GLP-1-Am with the addition of pre-formed oligomers. Samples of 50 (**A**) and 25 µM (**B**) GLP-1-Am in 25 mM phosphate at pH 8 with and without the addition of 2 % (w/w) of pre-formed purified GLP-1-Am oligomers were incubated with 50 µM thioflavin T at 37 °C with agitation for 6 days. All samples were prepared in a triplicate. GLP-1-Am oligomers were pre-incubated in 25 mM phosphate pH 8 for 5 days and then isolated using SEC.

References:

1. Whitmore, L., and B.A. Wallace. 2008. Protein secondary structure analyses from circular dichroism spectroscopy: Methods and reference databases. *Biopolymers*. 89:392–400, doi: 10.1002/bip.20853.

2. Whitmore, L., and B.A. Wallace. 2004. DICHROWEB, an online server for protein secondary structure analyses from circular dichroism spectroscopic data. *Nucleic Acids Res.* 32:668–673, doi: 10.1093/nar/gkh371.

3. Sreerama, N., and R.W. Woody. 2000. Estimation of Protein Secondary Structure from Circular Dichroism Spectra: Comparison of CONTIN, SELCON, and CDSSTR Methods with an Expanded Reference Set. *Anal. Biochem.* 287:252–260, doi: 10.1006/abio.2000.4880.

4. Schuck, P. 2000. Size-distribution analysis of macromolecules by sedimentation velocity ultracentrifugation and Lamm equation modeling. *Biophys. J.* 78:1606–1619, doi: 10.1016/S0006-3495(00)76713-0.
